# Genomic competition for noise reduction shaped evolutionary landscape of mir-4673

**DOI:** 10.1038/s41540-020-0131-2

**Published:** 2020-05-06

**Authors:** Ramin M. Farahani, Saba Rezaei-Lotfi, Neil Hunter

**Affiliations:** 1 IDR/Westmead Institute for Medical Research and Westmead Centre for Oral Health, Sydney, NSW Australia; 2 Faculty of Medicine and Health Sciences, University of Sydney, Sydney, NSW 2006 Australia

**Keywords:** Genetic interaction, Origin of life, Cellular noise

## Abstract

The genomic platform that informs evolution of microRNA cascades remains unknown. Here we capitalised on the recent evolutionary trajectory of hominin-specific miRNA-4673, encoded in intron 4 of notch-1, to uncover the identity of one such precursor genomic element and the selective forces acting upon it. The miRNA targets genes that regulate Wnt/β-catenin signalling cascade. Primary sequence of the microRNA and its target region in Wnt modulating genes evolved from homologous signatures mapped to homotypic *cis*-clusters recognised by TCF3/4 and TFAP2A/B/C families. Integration of homologous TFAP2A/B/C *cis*-clusters (short range inhibitor of β-catenin) into the transcriptional landscape of Wnt cascade genes can reduce noise in gene expression. Probabilistic adoption of miRNA secondary structure by one such *cis*-signature in notch-1 reflected selection for superhelical curvature symmetry of precursor DNA to localise a nucleosome that overlapped the latter *cis*-cluster. By replicating the *cis*-cluster signature, non-random interactions of the miRNA with key Wnt modulator genes expanded the transcriptional noise buffering capacity via a coherent feed-forward loop mechanism. In consequence, an autonomous transcriptional noise dampener (the *cis*-cluster/nucleosome) evolved into a post-transcriptional one (the miRNA). The findings suggest a latent potential for remodelling of transcriptional landscape by miRNAs that capitalise on non-random distribution of genomic *cis*-signatures.

## Introduction

Genetic noise refers to cell–cell variability of gene expression that is measured based on the coefficient of variation of a signal across a population^1^. Buffering of transcriptional noise^2,3^ and its amplification^4^ are evolutionary mechanisms that improve phenotypic stability by canalisation^5^ or induce somatic heterogeneity, respectively. MicroRNAs stabilise the evolutionary interface of genotype and phenotype by canalisation of development^6,7^ and improving the heritability of novel traits^5^. To regulate genetic noise, miRNAs hybridise to transcripts that encode complementary “seed” sequences and uncouple the subsequent translation of the mRNAs or trigger the endonucleolytic cleavage of targeted transcripts^8^. As such, genic distribution of the “seed” sequences is a major factor that determines the potential interactions of miRNAs and the system-level manifestations of the interactions.

Random distribution of the seed region in various genes (1 in 4^6^ bp for 6-mer seed regions) and a large-scale mutational drift subsequent to evolution of an individual miRNA can potentially adjust the specificity of the putative interactome to the estimated ≈100 target sites^9^ per miRNA. The latter view, however, conflicts with several major lines of evidence. Unlike 3′-untranslated regions (3′-UTR), sequences in the coding regions^10^ are highly conserved and not easily removed by mutational drift from the potential interactome of a newly evolved miRNA. Further, small genes such as those encoding olfactory receptors are not negatively biased against microRNA regulation^11^ despite lexical simplicity. Finally, emerging evidence suggests the importance of base pairing beyond the seed region in order to improve the specificity of targeting by miRNAs^12^. A plausible explanation for complementarity between miRNA-targets is that homologous regions evolve independently and yet simultaneously and are eventually co-opted by evolving miRNAs. In the current paper, we probe the former two propositions, co-option versus de novo evolution, by exploring the evolutionary trajectory of miR-4673^13,14^ that is encoded in the notch-1 locus.

Recent work in our laboratory demonstrated that miR-4673 can efficiently reprogramme the population dynamics of proliferating cells by stimulating a synchronised mode of cycling^13,14^ that remarkably reduces cell–cell variability of the population. Mechanistically, this occurs by miR4673-mediated induction of autophagy^13^ that alters the balance of competition between Notch-1 and β-catenin^15,16^ in favour of the latter protein^14^. The competition between Notch-1 and β-catenin codes a bistable developmental switch^15^ that integrates system-level signalling inputs into a binary outcome. Resolution of the proliferation/differentiation dichotomy under instruction from the Wnt/β-Catenin pathway^15^ is an example of the binarization activity of the cascade. It therefore is not surprising that altered binarization threshold of the Wnt/β-catenin signalling cascade leads to profound morphological novelties. In the nervous system, for example, enhanced activity of Wnt cascade propels significant expansion of the cerebral cortex^17^. As such, fluctuations (noise) in the level of free cytoplasmic β-catenin are buffered by multiple parallel post-translational mechanisms^18^. Yet, unless transcriptional noise is controlled, fluctuations in post-translational modulators of β-catenin lead to an altered binarization threshold of the Wnt/β-catenin signalling cascade. Here, we provide evidence for the evolution of miR-4673 and the interacting homologous regions by co-option of transcriptional noise dampeners in notch-1^15,16^ and other post-translational noise modulators of the Wnt/β-catenin signalling cascade.

## Results

### Structural features of miR-4673 were instructed by an intragenic enhancer of notch-1

**Fig. 1:**
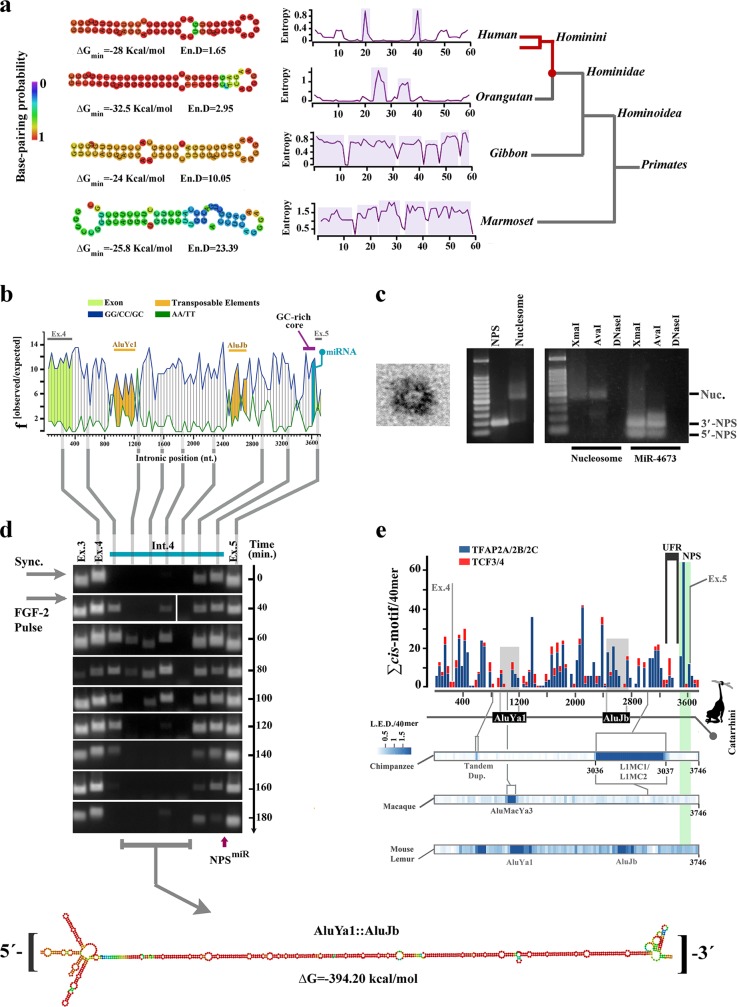
Intronic precursor of miR-4673 in notch-1 codes an active enhancer. **a** Structural analysis shows improved thermodynamic stability of the RNA hairpin in the primate lineage culminating in structural maturation of the miR-4673 hairpin in *Hominins*. **b** Nucleosome-favouring dinucleotide usage map of human notch-1 intron 4. **c** Transmission electron micrograph of a nucleosome formed by NPS^miR^ (left). Gel retardation (middle) and restriction enzyme digestion assays (right) provided further confirmation for nucleosome formation by NPS^miR^. **d** The temporal profile of enhancer-RNAs (eRNAs) that originate from notch-1 intron-4 (full blots are provided in Supplementary Fig. 4). Vertical lines show the location of eRNAs with reference to the dinucleotide usage map. In order to access the eRNA profile, cells were synchronised at G0 and released into G1 by application of a single pulse of FGF-2. Note the temporal stability of 3′-eRNA from a region that corresponds to NPS^miR^ (see Supplementary Fig. 5). This region is positioned at the 3′-terminus of a stable RNA palindrome formed by two Alu elements. **e** Histogram shows cumulative distribution of TCF3/4 and TFAP2A/B/C *cis*-motifs in the intron 4 of human notch-1. At the bottom, Levenshtein distance (L.E.D) as a measure of intronic change is aligned to the enhancer map. Sequences that belong to AluJb and AluYa1 insertions in all Simians are in grey. Species-specific transpositional events are marked in LED heat maps.

Pre-miRNAs are characterised by a secondary structure (imperfect stem loop) that licences processing by dicer^19^ and a primary sequence that instructs the specificity of targeting. We began by addressing evolutionary forces that informed secondary structure of the miRNA in *Hominins* (Fig. 1a). The primary sequence of miR-4673 intronic precursor in notch-1 had a bendable G/C-rich core and inflexible A/T-rich flanking regions (Fig. 1b). This arrangement is consistent with stringent human nucleosome positioning sequences (NPS) that predict well-positioned nucleosomes in vivo^20^. The propensity of miR-4673 intronic precursor to communicate with histones was validated experimentally. In vitro, a synthetic NPS corresponding to miR-4673 and immediate flanking sequences (NPS^miR^: 5′-[A]_4_TGA[T]_2_C[T]_3_…TGA[G]_2_[A]_4_TAT-3′) readily reconstituted a stable nucleosome assembly with recombinant human H2A/H2B/H3.1/H4 histone octamers (Fig. 1c). It is known that an additional signature of a high affinity NPS is the palindromic nature of NPS primary sequence that generates superhelical curvature symmetry and accommodates twofold dyad symmetry of histone octamers^21^. This feature improves the translational (i.e. positional) stability of nucleosomes. We reasoned that evolution of the pre-miRNA hairpin might in part reflect structural adaptation of the NPS^miR^ for stable translational localisation of a nucleosome through palindromic superhelical symmetry. As translational stability is critical for nucleosomes that protect and regulate access to enhancers^22^, we investigated the enhancer activity of intron 4.

Using primary human neural progenitor cells, high-resolution nascent RNA-based temporal fingerprinting of enhancer RNA^22^ was employed to access active intra-genic enhancers^23,24^ in intron 4 of notch-1. An active enhancer was identified in intron 4 of notch-1 (Fig. 1d). The most active 3′-terminus of the enhancer in intron 4 was mapped to the NPS^miR^ and the upstream flanking region of NPS^miR^ (UFR^miR^: 5′-[C]_2_[TG]_2_[GA]_2_CA…AGT[C]_2_TGT[C]_2_-3′). The 3′-enhancer encoded a combinatorial *cis*-module^25^ characterised by TCF3/4 binding clusters in UFR^miR^ and TFAP2A/B/C binding motifs in NPS^miR^ (Fig. 1e, detailed in Supplementary Table 1). TFAP2A/B/C family members act as short-range repressors of flanking TCF3/4^26^. The NPS^miR^, on the other hand, can position a +1 nucleosome^27^ (referenced to the enhancer) that stalls RNA polymerase-II (RNAP-II)^28^ and delays transcriptional elongation. Both these features (short-range repressor and +1 positioned nucleosome) can reduce notch-1 transcriptional noise^29^ by increasing the activation threshold of the enhancer. Corroborating this notion, we observed more stable and consistent activity (Fig. 1d) of the 3′-terminus of the enhancer (corresponding to UFR^miR^ + NPS^miR^) compared to the 5′-terminus (exon4-intron4 junction) of the enhancer (Fig. 1d).

**Fig. 2:**
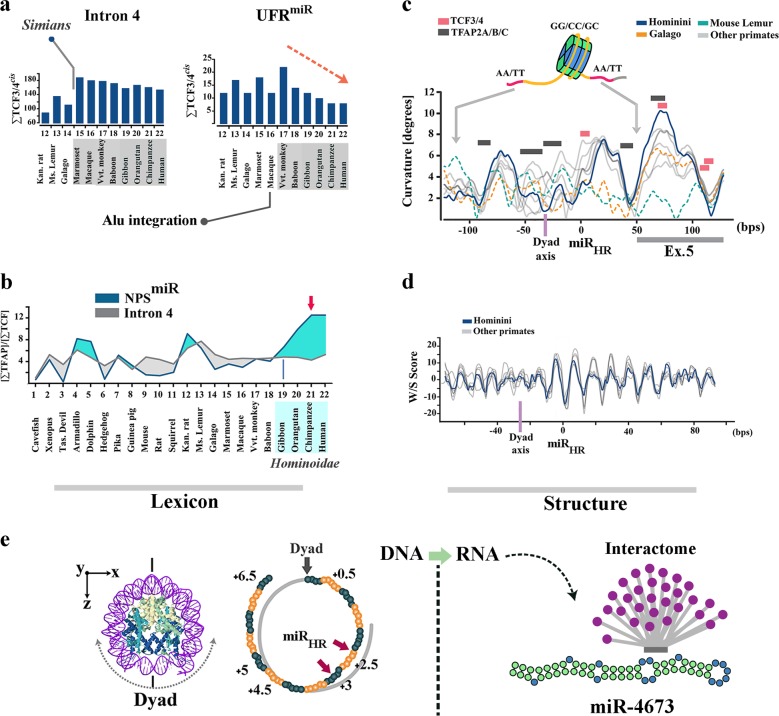
Evolutionary trajectory of the structural features of notch-1 intronic enhancer is aligned to structural maturation of the miR-4673. **a** TCF3/4 recognition motifs in intron 4 were reduced in higher primates. Depletion of TCF3/4 recognition motifs was more obvious in the upstream flanking region of NPS^miR^ (UFR^miR^) (arrow shows the depletion trend of motifs). **b** TFAP2A/B/C binding motifs were enriched in NPS^miR^ toward the higher primates. Lines demonstrate [TFAP2A/B/C]:[TCF3/4] ratio in various mammalian species. **c** In primate lineage, tendency of NPS^miR^ to curve symmetrically gradually increased towards *Hominins* (top diagram, note the increased symmetry of the blue line with reference to the dyad region). This change can improve the translational stability of the nucleosome positioning sequence. **d** DNA anisotropy evidenced by W/S dinucleotide oscillations did not change significantly in the primate lineage. **e** The miR_HR_ occupies superhelical locations +2.5 and +3 in the NPS with the palindromic sequence at superhelical locations +4.5 and +5 (middle). The superhelical symmetry accommodates twofold dyad symmetry of the nucleosome core particle and associated DNA (left, PDB ID: 3REI). In the transcribed RNA, superhelical positions +1 to +6.5 (65 bp) code pre-miR-4673.

**Fig. 3:**
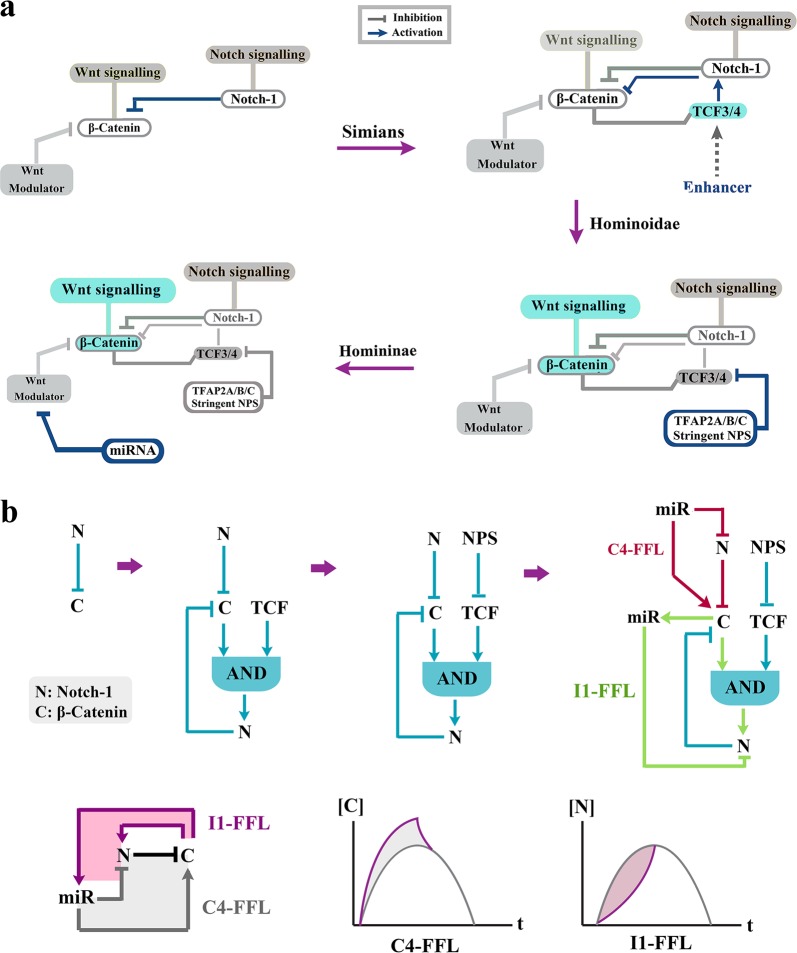
Evolution of network topology of Notch-1/β-catenin in the primate lineage. **a** Schematic diagrams demonstrate gradual evolution of network topology of the Notch-1/β-catenin axis in prosimians, simians, Hominoidae and Homininae. Note that sophistication of the topology parallels enhances signalling activity of the Wnt/β-catenin in higher primates. **b** Schematic diagrams (top) demonstrate gradual evolution of the network topology by recruitment of additional network motifs^76,77^ into the existing circuitry of Notch-1/β-catenin axis. Evolution of the microRNA radically alters^78^ the existing genetic network. Evolution of miRNA-4673 in Homininae generates a type-4 coherent feed-forward loop (C4-FFL) that amplifies activity of the Wnt/β-catenin (bottom). Simultaneously, inclusion of Notch-1 in a type-1 incoherent feed-forward loop (I1-FFL) formed by the miRNA reduces the signalling activity of the notch signalling pathway (bottom).

Enrichment of TCF3/4 *cis*-cluster in the intron 4 of notch-1 occurred prior to divergence of Simians (Fig. 2a). Thereafter, selection of structural features that could instruct noiseless Wnt-mediated activation of Notch-1 gained significant momentum in the common ancestor of *Hominoidae* (Fig. 2b, c). One such feature was significant enrichment of binding motifs for TFAP2A/B/C family members (short-range repressors of β-catenin^26^) (Fig. 2b). In *Hominins*, another structural change in the region that corresponds to NPS^miR^ was selection for more stringent histone-independent translational positioning of +1 nucleosome through enhanced superhelical curvature symmetry^30^ (Fig. 2c). Notably, mutational selection for superhelical symmetry of NPS^miR^ did not compromise the rotational nucleosome positioning that relies upon periodic W/S dinucleotide oscillations of the anisotropic NPS^31^ (Fig. 2d). Based on the evidence provided, we suggest the following plausible scenario for evolution of miRNA-4673. It is likely that structural evolution of the miRNA was as a bystander outcome that reflected selection for the improved superhelical symmetry of the NPS^miR^ with a primary lexicon inherited from the associated *cis*-cluster (Fig. 2e). We also suggest that natural selection of the miRNA could be due to reconfiguration of the competitive interface of notch-1 and β-catenin in favour of the latter protein (Fig. 3) leading to a further reduction of Notch-1 variability. We next probed the putative evolutionary scenarios for selectivity of targeting by miR-4673 (evolution of the interactome).

### Evolutionary trajectory of miR-4673 targets mirrors structural maturation of the miRNA

**Fig. 4:**
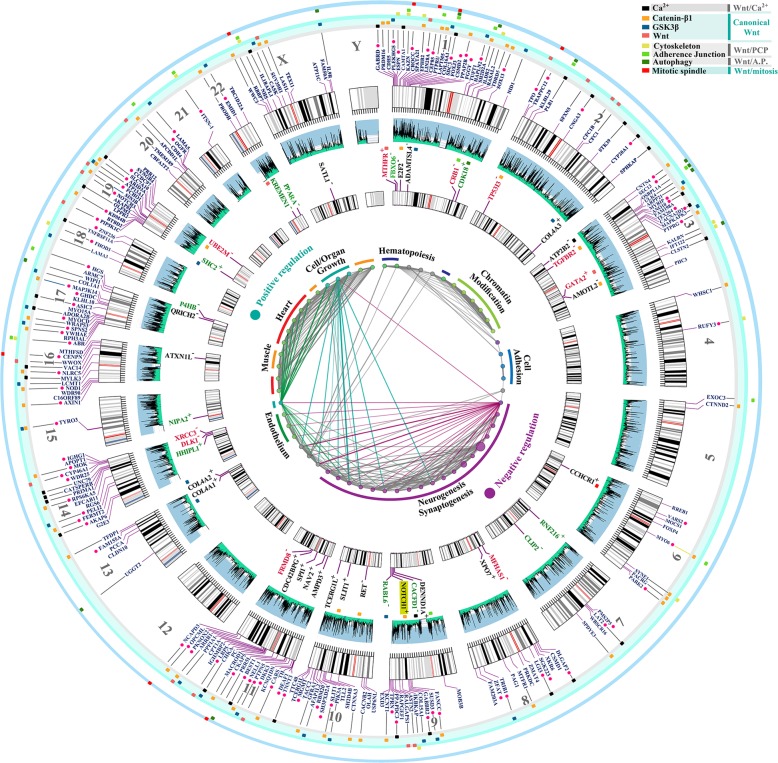
Spatial and functional clustering of homology to miR-4673. Genomic distribution of miR-4673 interactome shows distinct gene clusters localised to H3^+^ isochores (middle circular diagram). The miRNA homology regions (miR_HR_) were scattered in coding sequences (inner karyogram genes in black), 5′e-UTRs (inner karyogram genes in red), 3′e-UTRs (inner karyogram genes in green) and i-UTRs of human genes (outer karyogram). Sense and anti-sense directionality of (miR_HR_) in the inner karyogram is designated as + and −. In the outer karyogram red circles indicate the genes with miR_HR_ in antisense direction to miR-4673. Genes with miR_HR_ signature are involved in regulating development of cardiovascular and nervous systems (inner diagram). These genes act as calibrators (tissue-specific modulators) of Wnt signalling by fine-tuning canonical and non-canonical modules of the cascade (see Supplementary Discussion 1 for detailed elaboration). The outer colour-coded layer designates the interactome genes to canonical Wnt and non-canonical Wnt/Ca^2+^, Wnt/PCP (planar cell polarity), Wnt/Autophagy (A.P.) and Wnt/Mitosis cascades.

**Fig. 5:**
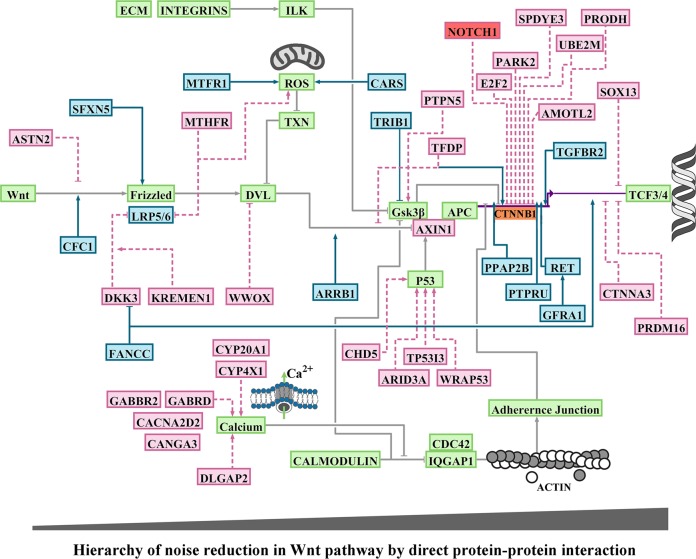
Functional mapping of genes bearing homology to miR-4673. BioTapestry visualisation of the Wnt/β-catenin pathway and the associated modulators. Green boxes demonstrate genes that comprise the backbone of the Wnt/β-catenin pathway and that do not code miR_HR_. The blue and red boxes are miR_HR_-coding positive and negative regulators of β-catenin, respectively (detailed in Supplementary Discussion 1). Genes that code miR_HR_ are mainly involved in direct regulation of β-catenin. Indirect regulation of β-catenin occurs by modulating the stability of Cadherin-based junctions that in turn recruit β-catenin. Another major cluster of genes orchestrate calcium flux and hence the stability of cadherin-based junctions.

To resolve evolutionary trajectory of the miRNA targets, we decided to expand the homology requirement beyond the established 6-mer seed region^12^. We also expanded the homology region criteria to sequences that are positioned within the intronic, coding and the 5′-untranslated regions^10^. The miR-4673 homology regions (miR_HR_) were defined based on sequence homology to miR-4673 (Homology_min_ > 68%), conserved [5′-GGCTCCTGCC-3′] consensus sequence (± strand) and [miR–miR_HR_]^ΔG^ < −36 kcal/mol [see Methods]. The choice of consensus sequence [5′-GGCTCCTGCC-3′] was based on previous experiments that determined high affinity targets of miR-4673^13^. To trace global chromosomal coordinates of putative targets_,_ we used BLAST analysis of miR_HR_ against human genomic sequences (GRCh38). The analysis revealed non-random distribution of miR_HR_ in dense gene clusters associated with GC-rich H3^+^ (GC > 52%) isochores (Fig. 4). Homology regions were identified in coding sequences (CDS), untranslated exonic regions (5′ and 3′e-UTRs) and introns (i-UTR) of human miR_HR_-coding genes in sense (miR_HR_^+^) or anti-sense (miR_HR_^−^) strands (Fig. 4). Genes signified by miR_HR_ modulated canonical and non-canonical Wnt signalling cascades^32^ (Fig. 5) as detailed in Supplementary Discussion 1. We concluded that miR_HR_ is a signature of a genic cluster whose protein products regulate the cytoplasmic availability of β-catenin as the main determinant of noise in the Wnt pathway (Fig. 5).

**Fig. 6:**
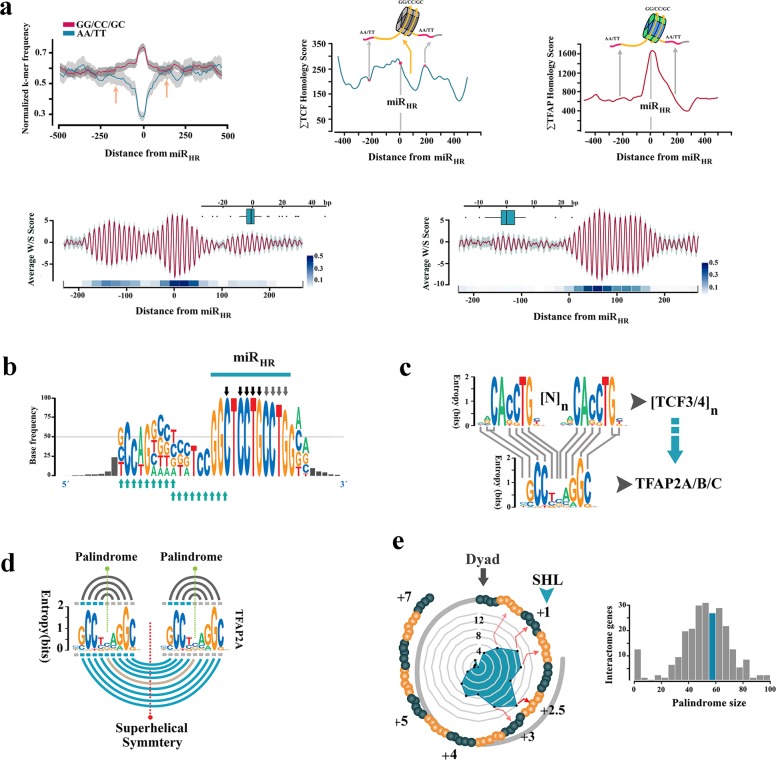
Adaptive evolution of miRHR enhancers in target genes. **a** Average dinucleotide usage map in target genes after symmetrisation to miR_HR_ (left; arrows indicate AA/TT-rich boundaries). Enrichment of TCF3/4 upstream and TFAP2A/B/C downstream to miR_HR_ in symmetrised sequences (middle, right). Background W/S dinucleotide oscillations (bottom) overlapped the TCF3/4 cis-clusters (left; *n* = 101) or TFAP2A/B/C clusters (right, *n* = 86) (red line: mean, grey margin: bootstrapped confidence interval). Box plots show phase offset between oscillations symmetrised to miR_HR_ (linear heat map; see methods). The linear heat maps demonstrate the average value for pair-wise cross-correlation of 20-mer fragments in structurally aligned NPS sequences. **b** Consensus motif for the miRNA hybridisation region (in RNA world) is chimeric with TCF3/4 *cis*-motifs (black and grey arrows) in miR_HR_ and TFAP2A/B/C binding motifs (turquoise arrows) upstream to it. **c** Mutational revision of tandem TCF3/4 *cis*-motifs ([*N*]_*n*_: gap between tandem repeats) can generate TFAP2A/B/C recognition motif. **d** Tandem repeats of palindromic TFAP2A/B/C *cis*-motifs (grey lines) coerce a supersymmetry in the underlying DNA (turquoise lines). **e** SymCurv analysis of targets with peak TFAP2A/B/C values in the miR_HR_ region (*n* = 46 genes) localised the signature to the superhelical locations (SHL) + 2.5 and +3 analogous to the miRNA in Fig. 2e (left). The right diagram shows hairpin size that is formed by miR-4673 target regions in the interactome genes (Supplementary Fig. 3).

**Fig. 7:**
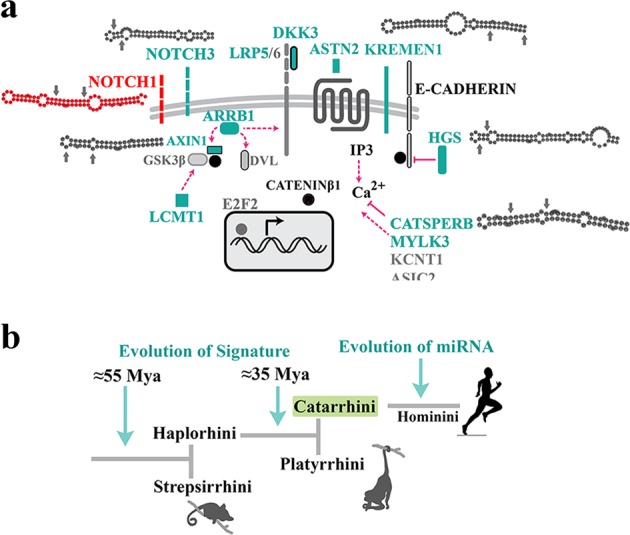
MiR-4673 targets are dormant immature pre-miRNAs. **a** Near-perfect RNA stem-loops were identified in key modulators of Wnt cascade that are targeted by the miRNA. Selection for structural features of DNA that improve nucleosome positioning propels a parallel journey in the RNA world to superimpose a stem loop signature in the miR_HR_ region of most targets similar to miR-4673 secondary structure. Arrows designate miR_HR_ in the hairpins. **b** Co-option of transposable elements into *cis*-clusters expanded the extant *cis*-clusters during the Eocene epoch prior to divergence of Catarrhini. Afterwards, a period of dormancy culminated in the evolution of the miRNA.

To unfold the genomic context of homology regions, all miRNA target regions were symmetrized to miR_HR_ prior to structural analysis (Fig. 6a). The aligned sequences demonstrated a G/C-rich central core and inflexible A/T-rich boundary sequences similar to NPS^miR^ (Fig. 6a, left). Symmetrisation to miR_HR_ also polarised the aligned sequences into homotypic TCF3/4 *cis*-clusters positioned upstream to miR_HR_ and TFAP2A/B/C *cis*-clusters that flanked miR_HR_ downstream to it in a W/S dinucleotide oscillatory background (Fig. 6a). The latter *cis*-anatomy closely replicated that of the NPS^miR^ in notch-1 intron-4 as described previously. Based on this observation, a plausible scenario is that targets and the miRNA embarked upon parallel evolutionary trajectories prior to maturation of a functional miRNA. The journey culminated at establishment of bipartite *cis*-clusters that can coordinate input into Wnt modulator genes by β-catenin (via TCF3/4 module) in a noise-free manner (by TFAP2A/B/C activity and the associated NPS). The miR_HR_ is a chimeric junctional signature of the latter *cis*-modules (Fig. 6b); a contention bolstered by symmetrisation of *cis*-clusters to the miR_HR_ (Fig. 6a). Notably, genomic regions specialised to recognise TCF3/4 clusters with high affinity can also be recognised by TFAP2A/B/C due to the similarity of the *cis*-lexicon (Fig. 6c). The latter observation in part explains diminished randomness in evolution of the described bipartite *cis*-clusters where functional hierarchy in Wnt cascade is aligned to the enrichment of TCF3/4 *cis*-clusters that in turn facilitates evolution of TFAP2A/B/C *cis*-clusters. Enrichment of TFAP2A/B/C *cis*-clusters not only can reduce TCF-dependent transcriptional noise but also improves superhelical symmetry of the underlying NPS (Fig. 6d, e). Therefore, selection for transcriptional noise buffering by enrichment of TFAP2A/B/C *cis*-motifs and superhelical symmetry of NPS in the DNA world could potentially empower a parallel and yet dormant journey towards palindromic symmetry in the RNA world (Figs. 6e and 7a). We interpreted the parallel structural maturation of the miRNA and the targets as a ‘competition’ for transcriptional noise reduction. After final maturation of miRNA structural signature by notch-1 NPS^miR^, homologous regions in CDS, 5′ and 3′ e-UTRs of the competing genes were co-opted by the miRNA as its interactome (Fig. 4, inner karyogram). To that end, autonomous transcriptional noise filtering capacity of NPS in target genes was amplified and temporally tuned to transcriptional activity of the miRNA and its host gene notch-1. However, conservation of NPS^miR^ in the intronic regions that are not targeted prior to evolution of the miRNA indicates the importance of autonomous transcriptional noise filtering activity of NPS in the target genes.

Reduction of noise in the transcriptional landscape of Wnt modulator genes by recruitment of bipartite enhancers occurred during the Eocene epoch and prior to divergence of Platyrrhine (≈56-33.9 Mya) by mutational remodelling of the extant genomic landscape or integration of transposable elements (Fig. 7b, Supplementary Table 2). This phase ceased before divergence of AluY transposable elements^33^ in Simians (Fig. 7b, Supplementary Table 2). Thereafter concurrent with the Eocene–Oligocene extinction event (~34 Mya)^34^ progressive selection of features that enhance translational stability of nucleosomes, e.g. superhelical symmetry and AA/TT boundary constraints, occurred. To uncover the selective advantage that stabilised miR-4673 in hominins after Pliocene, we investigated post-transcriptional reduction of genetic noise at a high temporal resolution.

### Activity of miR-4673 temporally complements the noise-filtering capacity of notch-1

**Fig. 8:**
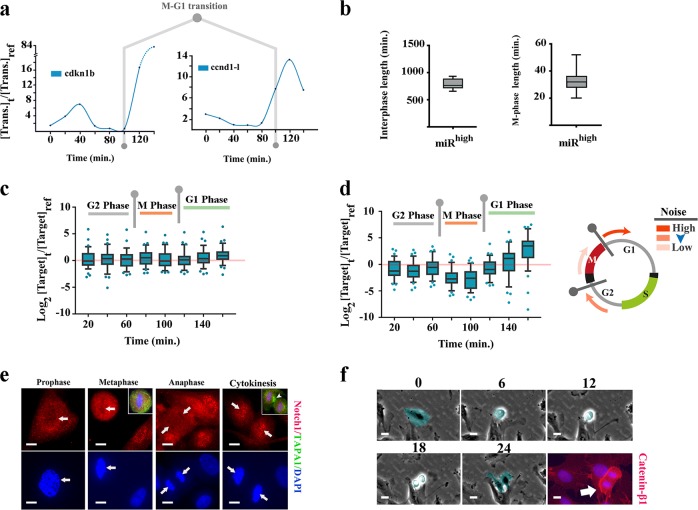
Cell cycle-specific modulation of transcriptional noise by miR-4673. **a** Transcriptional fingerprint of cyclin-dependent kinase-1b (cdkn1b) and cyclin-D1-long isoform (ccnd1-l) was used as a proxy for cell cycle profile of the dividing population. The rising levels of ccnd1-l transcript at *t*_120_ is consistent with M–G1 transitional phase. **b** The length of M phase and interphase was measured using single-cell live imaging analysis of dividing cells (*n* = 20 cells). Based on the average length of M phase, *t*_80_ and *t*_100_ temporal windows accommodate the transcriptional profile of M phase. **c** Each box plot demonstrates the pooled expression levels of miR-4673 targets (*n* = 43 inner karyogram genes of Fig. 4) at specific time points ([Target]_*t*_) normalised to the expression levels of the same genes at time zero ([target]_ref_). Note that in the absence of exogenous miR-4673 the temporal expression profile shows a slight depression at *t*_100_ followed by an increase at *t*_160_. **d** Application of exogenous miR-4673 amplifies the temporal transcriptional signature of miR-4673 targets. Amplified suppression of the transcriptional profile prior to *t*_120_ anticipates the marked increase in the expression level of targets at *t*_140_ and *t*_160_. **e** Notch-1 fingerprinting shows absence of the activated protein in the nuclear compartment during M phase and the re-emergence concurrent with cytokinesis (top left). Cell surface glycoprotein, TAPA-1, demarcates the contractile ring formed during cytokinesis (Scale bars: 5μm). **f** Single-cell tracking of proliferating cells demonstrated increased level of cytoplasmic β-catenin after mitotic cell rounding and prior to cytokinesis (numbers: time points in min.) (Scale bars: phase contrast images 3 μm, bottom right micrograph 5 μm).

We inferred the impact of miR-4673 on transcriptional noise based on relative temporal availability of the targeted transcripts with reference to cell cycle in the control and the transfected cells. The transcriptional activity of genes that encoded miR_HR_ in coding sequences and untranslated 5′ and 3′-exonic regions (genes of inner karyogram in Fig. 4) was measured using real-time quantitative polymerase chain reaction (qPCR). Preliminary experiments demonstrated relative synchronisation of the population-level cell cycle after 20 h at G0 phase of cycle^13^. Based on preliminary experiments, the first measurement (*t*_0_) was carried out 19 h after electroporation of the cells with naked miR-4673 (200 nM/10^6^ cells). Thereafter, transcriptional activity was measured at regular intervals every 20 min for the next three hours. Transcriptional activity of cyclin-dependent kinase-1b (cdkn1b) and cyclin-D1 long isoform (ccnd-1l) was measured to fingerprint the phases of cell cycle precisely (Fig. 8a). Cyclin-D1 is required for progress from G1 to S phase^35^ and cdkn1b (p27) regulates the assembly and activation of CDK4 and Cyclin D1^36^. Therefore the transcriptional activity of these two genes increases immediately after M phase to propel progress in G1 phase. We noted a significant boost in the activity of ccnd1l and cdkn1b at *t*_100_ (fifth time point or *t* = 100 min) and *t*_120_ (*t* = 120 min) (Fig. 8a). This transcriptional fingerprint was consistent with population-level transition from M to G1 phase of cell cycle at *t*_100_. On the other hand, completion of M phase required ≈30 min as measured by high-frequency single cell tracking of proliferating cells (Fig. 8b). Hence, the temporal window from *t*_80_ to *t*_100_ corresponded to an M phase-rich period in the population of cycling cells. The temporal points prior to *t*_80_ and after *t*_100_ were mapped to G2-rich and G1-rich windows, respectively.

Following the amplification of miR-4673 (miR^high^ cells), we noted a bimodal reprogramming of the transcriptional landscape of the targets (Fig. 8c, d). Amplification of the miRNA suppressed the transcript availability of target genes particularly at t_80_ and t_100_ (Fig. 8d, detailed in Supplementary Fig. 1). Hence, maximum transcriptional suppression by miR-4673 of the targets occurred during M phase of cell cycle (*t*_80_ to *t*_100_). The enhanced efficiency of miRNA-mediated suppression during M phase could be a reflection of differential activity of the RNA interference machinery in various phases of cell cycle with an increasing demand during M phase^37,38^. The transcriptional silencing by miR-4673 would reduce the standard deviation of targeted transcripts (i.e., transcriptional noise) by confining the upper limit of transcript variability^3^. For a single gene, standard deviation (S) of a transcript copy number (*x*_*i*_) in a population of cells would have an upper limit if the maximum copy numbers for that gene in individual cells converge towards the central value in population

1 $$S = \sqrt {\frac{1}{{n - 1}}\mathop {\sum}\limits_{i = 1}^n {(x_i - \bar x)^2} }$$

$$\max (x_i) \to \bar x\quad \Rightarrow \quad {\mathrm{S}} \to {\mathrm{S}}_{\min }.$$

**Fig. 9:**
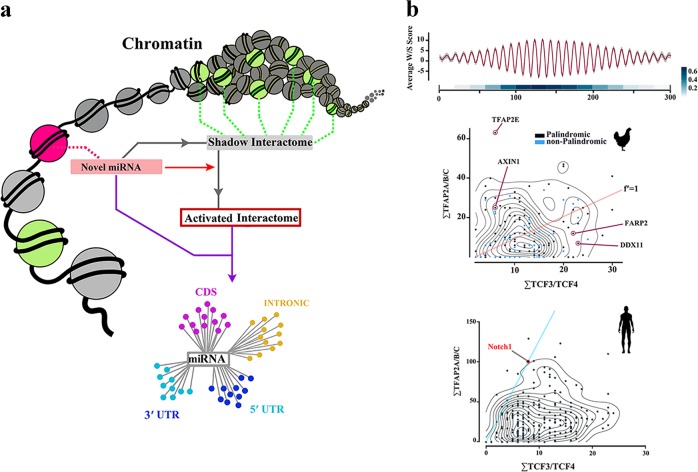
‘Shadow interactome’ and rewiring of genomic connectivity. **a** Schematic demonstration of a shadow interactome evolved in a genomic context and in the absence of a functional miRNA. Members of the shadow interactome may complete their structural maturation towards a hairpin that is recognisable by Dicer and co-opt the other members as an activated interactome. **b** Genomic features of a ‘shadow interactome’ in *Gallus gallus* with sequence homology to human miR-4673. The miR-4673 homology region in *Gallus gallus* (gga-miR_HR_) showed W/S dinucleotide oscillations characteristic of anisotropic NPS (top; red line: mean, grey margin: bootstrapped confidence interval). The linear heat map demonstrates pair-wise cross-correlation of 20-mer fragments in structurally aligned NPS. Bottom left scatterplot/contour map shows distribution of TFAP2A/B/C and TCF3/4 *cis*-motifs in gga-miR_HR_ gene cluster compared to human miR-4673 interactome. Note that enrichment of TFAP2A/B/C parallels generation of palindromic symmetry (black dots as opposed to blue dots clustered in the vicinity of red line). Also the lower TFAP2/TCF3/4 ratio in gga-miR_HR_ compared to human miR_HR_ suggests less stringent requirement for genetic noise reduction in chicken (red line: equal TFAP2/TCF3/4 ratio).

In other words, the upper limit (max *x*_*i*_) is maintained by miRNA-mediated buffering of transcriptional bursts. Therefore, miR-4673 reduces the upper limit of variability of the targeted transcripts by ≈55% at *t*_80_ and ≈65% at *t*_100_ (M phase). Notably, concurrent with entry into early G1, we noted enhanced transcriptional activity of the targets in the miR^high^ cells (Fig. 8d). This finding is consistent with the reported cell cycle-dependent switching of miRNA activity from being a silencer to an activator^39^. The maximum noise buffering by miR-4673 that occurs during M–G1 transition compensates for the absence of noise reduction by notch-1 (miR-4673 host gene) during mitosis (Fig. 8e). Concurrent with mitotic exit, the cytoplasmic level of β-catenin protein suddenly increases (Fig. 8f). This is partly due to suppression of β-TrCP1-Skp1 complex during M–G1 transition^40^ that enhances the level of β-catenin to propel cell cycle progression^41^. If the binary threshold for β-catenin availability is surpassed during the M–G1 transition, the cell commits to the next mitotic cycle by progress into G1. Therefore, noise buffering activity of miR-4673 during this phase could potentially impact upon proliferative capacity of the cycling cells by modulation of the activation threshold of Wnt/β-catenin pathway. We sought experimental proof for this notion by identification of an interactome in other species that encodes the miR_HR_ but is not yet targeted by any known miRNAs. Such a “shadow interactome” would anticipate and become active after structural maturation of a dormant miRNA and may accelerate acquisition of novel adaptive phenotypes at times of environmental crisis (Fig. 9a).

### Activation of a dormant interactome by miR-4673 reduces developmental noise in chicken embryogenesis

**Fig. 10:**
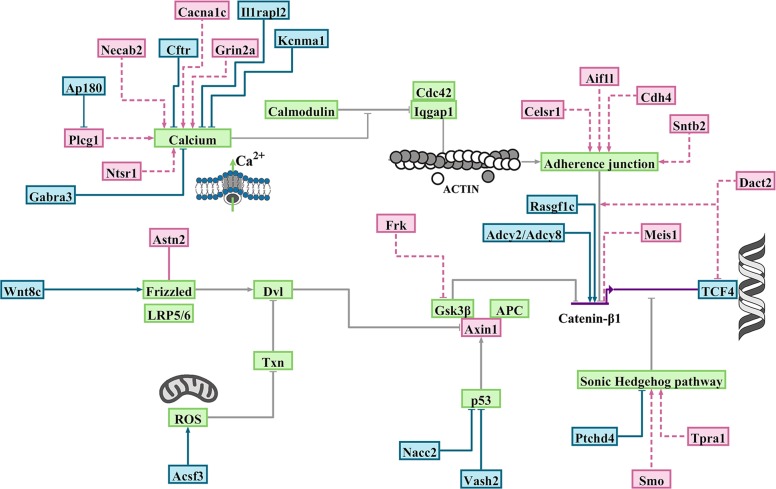
Functional mapping of *Gallus gallus* gene bearing homology to miR-4673. BioTapestry visualisation of Wnt/β-catenin pathway and the associated modulators. Green boxes demonstrate genes that comprise the backbone of the Wnt/Β-catenin pathway and that do not code miR_HR_. The blue and red boxes are miR_HR_-coding positive and negative regulators of β-catenin, respectively (Detailed in Supplementary Discussion 2). Some of the *gga*-genes that code miR_HR_ are involved in direct regulation of β-catenin. Most *gga*-interactome genes are indirect regulators of Β-catenin activity. Indirect regulation of β-catenin occurs by modulating the stability of Cadherin-based junctions that in turn recruit β-catenin. Another major cluster of genes orchestrate calcium flux and hence the stability of cadherin-based junctions. A small cluster of miR_HR_-bearing *gga*-genes communicate with sonic hedgehog cascade, a major antagonist of Wnt/β-catenin, to regulate the activity of the latter pathway.

**Fig. 11:**
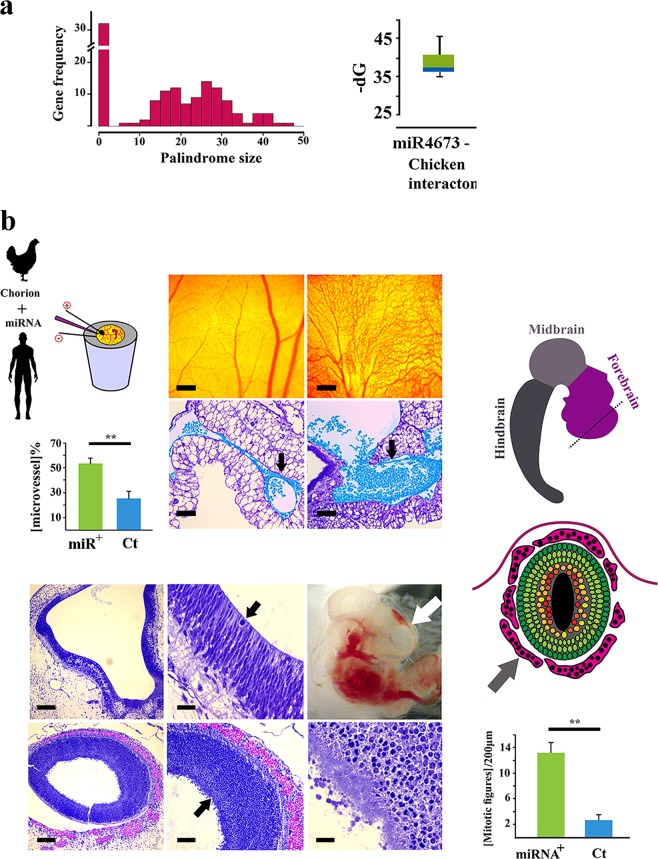
Experimental activation of “shadow interactome” in *Gallus gallus*. **a** Histogram (top left) shows the distribution of palindrome size in gga-miR_HR_ region. Box plot (top right) shows ΔG for miR-4673/*gga*-miR_HR_ hybridisation. The gga-miR_HR_ genes are central to several developmental signalling networks interfacing at Wnt cascade. Amongst gga-miR_HR_ genes several NPS from the Wnt cascade nearly achieved structural features of functional miRNAs. **b** Application of miR-4673 and activation of the identified gga-miR_HR_ shadow interactome boosted angiogenic activity in chorio-allantoic membrane (top). Blue pseudo-colourised elements demonstrate expansion of microvessels (black arrow) along with increased hematopoiesis. The miRNA also amplified hematopoiesis (red pseudo-colour) and neurogenesis (black arrows) in the explanted embryo (frontal cortex: white arrow) (error bars: s.d; ** indicates *p* < 0.01).

The miR_HR_ was targeted in *Mus musculus* by *mmu*-miR-3104-5p (Supplementary Fig. 2). Hence, *Gallus gallus* (*gga*) was chosen as an accessible, distant endothermic species with distinct GC-rich isochores. An orthologous chicken-specific shadow interactome with miR_HR_ NPS signature embedded in a simple W/S dinucleotide oscillatory background was identified that was not targeted by any known gga-miRNAs (Fig. 9b). An interactome map was generated to analyse targets of miR-4673 in chicken (Fig. 10, Supplementary Discussion 2). The gga-specific targets were also identified as modulators of Wnt cascade noise (Fig. 10, Supplementary Discussion 2). In ovo electroporation of human miR-4673 into chorio-allantoic membrane (CAM) of chicken embryo (HH16) triggered remarkable expansion of CAM vasculature (Fig. 11). Similarly, experimental application of miR-4673 to lateral ventricles of chicken embryo HH 23–25 (E4–4.5) followed by in ovo electroporation, triggered extensive synchronised and yet aberrant expansion of neural and hematopoietic precursors (Fig. 11). The enhanced mitotic activity was consistent with altered activity of Wnt/β-catenin pathway that is known to increase proliferative activity during organogenesis^17^. The cycle-independent application of the miR-4673 suffers from a lack of temporal fine-tuning due to the constant availability of the miRNA. The experiment, therefore, demonstrates the critical role of microRNA host genes in regulating the temporal dimension of miRNA-target interactions. We concluded that the dormant capacity of a shadow interactome can be accessed at times of evolutionary crisis by evolving miRNAs to propel remarkable evolutionary adaptations.

## Discussion

Our findings provide evidence for the evolutionary trajectory of miR-4673 by co-option (as opposed to de novo evolution) of genomic elements that operate to reduce transcriptional noise in the Wnt pathway. The proposed model suggests two major steps for the evolution of miR-4673. In the first stage and in the absence of the mature miRNA, selection of intragenic enhancers in Wnt cascade modulator genes seeded homologous primary sequences in a functional cluster of related genes. Throughout the next stage, competition for the reduction of transcriptional noise in Wnt cascade modulator genes (enrichment of TFAP2-binding motifs and palindromic symmetry) propelled structural maturation of miR-4673 in notch-1 while the other competing enhancers partially completed the journey.

Our findings regarding the evolution of pre-miR-4673 genomic sequence from a nucleosome positioning sequence are in agreement demonstrated enrichment of a positioned nucleosome on pre-miRNA genomic sequences^42,43^. Also in the light of the evidence for co-evolution of miRNA-target pairs^44^, it is surprising that the targets of miR-4673 demonstrate similar nucleosome positioning features. The co-evolution of miRNA-target pairs from homologous genomic structures predicts a degree of sequence homology that exceeds the reported 6-mer seed region criterion in animals. In a drastic contrast, plant miRNAs often demonstrate near-perfect complementarity to the targeted mRNAs^45,46^. While evidence is emerging regarding the importance of miRNA base-pairing beyond the conventional seed region in animal cells^12,47^, we believe that the dichotomy (seed region length) may in part result from the dynamic evolutionary landscape of plants compared to animals. Unlike animals, plants are stationary and hence rely on constant evolutionary adaptations by genomic innovations^48^. The rapid evolutionary rate of plant genes could provide a more accurate overview of miRNA-target homology for several reasons. The newly evolving microRNAs may exhibit near-perfect homology to the targets due to insufficient time required for mutational drift of the homology region. On the other hand, the redundant drifting miRNA-target interactions that generate the illusion of ‘seed region’ will be eliminated due to the rapid turnover rate of the genetic pool. This line of reasoning fits our observation of the near-perfect complementarity between miR-4673 and some of the targets that leads to the degradation of the targeted transcript, e.g., cdk-18^13^. We suggest that the recent evolutionary history of miR-4673 leaves insufficient time for mutational drift of the targets thereby replicating the hybridisation dynamics of plant microRNAs. In this scenario, the conventional 6-mer-based interactions are less important and belong to the drifting targets under instruction from other genomic forces. The latter hypothesis remains to be validated.

The findings reported herein attest to a remarkable potential for accelerated reprogramming of genomic connectivity through cis-regulatory elements concealed in non-coding DNA. Major evolutionary adaptations may be generated by subtle modulation of the transcriptional temporal domains (enhancers) that propel evolution of microRNAs. Findings of the present study may have broader implications for understanding the role of non-coding DNA in the evolution of complex metazoans.

## Methods

### Nucleosome positioning signature identification

An R script was written to calculate NPS of genomic sequences based on flexible GG/CC/GC-rich sequences and inflexible AA/TT-rich regions^20^. In order to construct the diagram shown in Fig. 1b of the main text, changes in NPS-favouring dinucleotide usage frequency in consecutive 40 mers of miR-4673 intronic precursor (5′-[A]_4_TGA[T]_2_C[T]_3_…[A]_2_CTGTGAGG[A]_4_TAT-3′] in Notch-1) were plotted according to the formula

2 $$f^{[{\rm{observed/expected}}]} = \frac{{f_{ob.}^\phi }}{{f_{ex.}^\phi }},$$

3 $$\begin{array}{l}f_{o\,b.}^\phi = \mathop {\sum}\limits_{i = l}^{20} {\phi _i/20} \\ \phi _i = \left\{ {\begin{array}{*{20}{c}} {1\quad {\mathrm{if}}\quad \varphi _i\; \in \left\{{\rm {G}}\,{\rm {G}},{\rm {C}}\,{\rm {C}},{\rm {C}}\,{\rm {G}},{\rm {A}}\,{\rm {A}},{\rm {T}}\,{\rm {T}} \right\}} \\ {0\quad {\mathrm{if}}\quad \varphi _i \notin \left\{ {\rm{G}}\,{\rm {G,C}}\,{\rm {C,C}}\,{\rm {G,A}}\,{\rm {A,T}}\,{\rm {T}} \right\}} \end{array}} \right.\\ f_{e\,x.}^\phi = \frac{1}{{\left( {\begin{array}{*{20}{c}} 4 \\ 1 \end{array}} \right)\left( {\begin{array}{*{20}{c}} 4 \\ 1 \end{array}} \right)}} = 0\,.\,0\,6\,2,\,5\end{array},$$

where, *f*^ϕ^_*ex*._ is the expected frequency of each dinucleotide and *i* is the dinucleotide position in each 40-mer (*i*_max_ = 20). The top line in Fig. 1b represents ∑(*f*^ϕ^_*ob.*_/*f*^ϕ^_*ex.*_)|ϕ∈(GG,CC,CG) and the bottom line is ∑(*f*^ϕ^_*ob*._/*f*^ϕ^_*ex*._)|ϕ∈(AA,TT).

### SymCurv analysis

SymCurv prediction of nucleosome positioning was performed as detailed elsewhere^30,49^. Briefly, curvature values of the sequence were calculated using BENDS^50^, through a sliding (1 bp) window of 30 bp, centred on each nucleotide. From the putative dyads defined as a local curvature minimum, highest SymCurv score, calculated based on the following equations, was selected as the putative dyad of choice

4 $$S_{\rm{sym}}(n,m) = \mathop {\sum}\limits_{i = 1}^m {\frac{1}{{\left| {{\rm{Curv}}_{n - i} - {\rm{Curv}}_{n + i}} \right|}}} ,{m}\; = 25,$$

5 $$S_{\min }(n) = \frac{1}{{({\rm{Curv}}_{n - 1} - {\rm{Curv}}_n) - ({\rm{Curv}}_{n + 1} - {\rm{Curv}}_n)}},$$

6 $${\rm{SymCurv}}_{(n,m)} = S_{{\rm{sym}}(n,m)}S_{\min (n)}.$$

Distance from the putative dyad region (*n* for SymCurv_(*n*,*m*)_ = Max) to miR_HR_ was calculated and the two flanking superhelical regions (SHL) accommodating the miR_HR_ were given a score of 1. All other SHL for that particular sequence were given a score of 0. Final score for each SHL was calculated as a sum of individual scores and shown as a radial line graph in Fig. 6e.

### Analysis of DNA anisotropy

Analysis of DNA anisotropy was accomplished using nuMap software^51^. The analysis is based on periodic occurrence of AT-containing (WW) and GC-containing dinucleotides (SS) in minor and major grooves and shown to favour rotational nucleosome positioning^31,52,53^. Briefly, W/S score is calculated as a moving average score centred at position *n* based on the following equation:

7 $$S_{W/S}(n) = \mathop {\sum}\limits_{{\rm{micor}}\_{\rm{site}} = 1}^{14} {C_{WW}} + \mathop {\sum}\limits_{{\rm{major}}\_{\rm{site}} = 1}^{12} {C_{SS} - \mathop {\sum}\limits_{\min {\rm{or}}\_{\rm{site}} = 1}^{14} {C_{SS}} } - \mathop {\sum}\limits_{{\rm{major}}\_{\rm{site}} = 1}^{12} {C_{WW}}.$$

The W/S dinucleotide oscillation plots were generated as sliding window of 1 bp.

### Structural alignment of DNA anisotropy plots

Structural alignment of the W/S dinucleotide oscillations was performed in two steps. Pairwise cross-correlation analysis (see next subheading) of sequences symmetrised to miR_HR_ provided a measure of phase offset which was subsequently curated manually for maximal cross-correlation of oscillations. The distribution of offset values (bps) was presented as a box plot in Fig. 6a. The aligned oscillations were subsequently presented as an averaged value (red line, Fig. 6a) and bootstrapped confidence interval (grey margin) using ggplot2 library of R platform.

### Cross-correlation analysis

To analyse the cross-correlation of W/S dinucleotide oscillations, structurally aligned W/S oscillations (see previous section) were divided into 20-mers using R platform. Pairwise normalised cross-correlation of the 20-mer was then carried out using the R platform based on the following formula:

8 $${\rm{norm}}\_{\rm{corr}}(x,y) = \frac{{\mathop {\sum}\nolimits_{n = 0}^{n - 1} {x(n) \ast y(n)} }}{{\sqrt {\mathop {\sum}\nolimits_{n = 0}^{n - 1} {x(n)^2 \ast \mathop {\sum}\nolimits_{n = 0}^{n - 1} {y(n)^2} } } }}.$$

Calculated pair-wise cross-correlation values in each position (each 20-mer) were presented as a linear heat map using ggplot2 library of R.

### PCR amplification of NPS^miR^

Human genomic DNA was isolated from primary human brain progenitors using Qiagen® DNeasy^TM^ Blood & Tissue kit. The DNA fragment corresponding to miR-4673 intronic precursor was amplified using the forward primer TCTTTCAAGCAGGGCGTGTCC and the reverse primer CTCACAGTTCTGGCCGGTGAA and Phusion^TM^ High-Fidelity DNA Polymerase (New England BioLabs®). PCR reaction comprised 2 μL of gDNA, 1 μL of 5 μM forward/reverse primers, 4 μL of 5× Phusion HF Buffer and 1 μL of 10 mM dNTPs, DMSO (final concentration: 2%), 0.25 μL of Phusion DNA Polymerase, and 10.25 μL of PCR-grade water.

### Nucleosome reconstitution

Recombinant human histone H2A/H2B dimer and recombinant human histone H3.1/H4 tetramer were purchased from NEB. Nucleosome was assembled with the salt dilution method as described elsewhere^54^. Briefly, recombinant histones (100 pmol of 20 μM H2A/H2B dimer, 50 pmol of 10 μM H3.1/H4 tetramer) were mixed with the amplified NPS^miR^ (50 pmol, 1:1 octamer to DNA ratio) and adjusted to 2 M NaCl and incubated at room temperature for 30 min. The reaction was serially diluted to 1.48, 1, 0.6 and 0.25 M NaCl by adding 10 mM Tris EDTA with 30-min incubations at room temperature in each dilution step.

### Gel mobility shift assay

The mobility shift assay was performed as described elsewhere^55^. Briefly, 10 µl of each sample (reconstituted nucleosome and DNA alone) was mixed with 2 µl of 100% glycerol. A 1% agarose gel was prepared using TB buffer (45 mM Tris, 45 mM boric acid). The agarose gel was run at 10 V/cm for 30 min at room temperature. All gels derive from the same experiment and they were processed in parallel.

### Restriction enzyme protection assay

A restriction map of the NPS^miR^ was prepared using NEB cutter. Two single cutter restriction enzymes (XmaI and AvaI from NEB) and DNAse-I (Life technologies, AM2222) were used to assess the protection of NPS^miR^ by histone in reconstituted nucleosome. The enzymatic digestion was accomplished in a solution comprised of 4 µl of reconstituted nucleosome, 1 µl of restriction enzyme, 1 µl of CutSmart® Buffer and 4 µl of Milli-Q water for 45 min at 37 °C. Digestion with DNAase-I was carried out in a solution comprised of 4 µl of reconstituted nucleosome, 1 μL DNase I (2 U), 1 μL DNase I Buffer, 4 µl of Milli-Q water for 30 min at 37 °C.

### Electron microscopy

Ultrastructural validation of nucleosome reconstitution was achieved by using transmission electron microscopy^56^. Special grids were prepared by carbon evaporation onto a collodion film supported by a carbon film. The solution containing reconstituted nucleosomes was applied to the positively charged collodion-carbon coated grids for 3 min and then stained with 2% uranyl acetate (in water), rinsed 3–4 times in milli-Q water, and dried on filter paper. The grids were then shadowed with platinum from 2 perpendicular directions under an angle of 7–10°. Samples were visualised using a Philips CM120 BioTWIN electron microscope.

### Cell culture

Human neural progenitors^57^ were purchased from ScienCell® (Carlsbad, CA). DMEM/F12 supplemented with 10% foetal calf serum (FCS), recombinant human FGF-2 (20 ng/ml, R&D Systems) and Antibiotic-Antimycotic® (100×, Life Technologies) was used for culturing the cells.

### Enhancer RNA fingerprinting

Several specific short primers were designed to fingerprint the enhancer activity based on the nascent RNA as per Supplementary Table 3 and purchased from IDTDNA®. The primers avoided two Alu transposable elements in the intron 4 of notch-1 to achieve maximum stringency and specificity. The enhancer region was analysed by mfold platform^58^ to confirm lack of significant secondary structure that could hinder binding of designed primers. Post-synchronisation at G0, cells were pulsed with FGF-2 (20 ng/ml) in fresh media and RNA was isolated to fingerprint the enhancer^59^.

### Reverse transcription and PCR

After DNase treatment, reverse transcription of extracted RNA was carried out using SuperScript-III reverse transcriptase and T4 gene 32 protein^60^ (Roche). PCR reactions (35 cycles) were then performed using the primers listed in Supplementary Table 4.

### *Cis*-motif profiling

PWMs from JASPAR 2016^61^ were used to identify the TFAP2A/B/C and TCF3/4 *cis*-motifs. A custom R script based on matchPWM function of Biostrings library was written to extract the *cis*-motifs of interest (homology threshold = 80%). A histogram was next generated to summarise the frequency of *cis*-motifs. Resolution of the histogram was optimised by adjusting bin width based on the method proposed by Shimazaki and Shinomoto^62^.

### Identification of transposable elements

Two platforms were used to identify and cross-check primate-specific transposable elements in sequences of interest; RAPBASE (Genetic Information Research Institute) and Dfam 2.0^63^.

### Palindromic symmetry

CoFold platform^64^ was utilised to simulate co-transcriptional folding of sequences of interest. In this platform, the global algorithm of choice for simulating RNA secondary structure was based on thermodynamic parameters proposed by Turner et al.^65^.

### Karyogram

Circos platform^66^ was used to generate the circular diagram of Fig. 4 of the main text. Isochore map corresponding to each chromosome was also generated using Circos platform by importing the results from GC-profile platform as explained in the following section.

### Isochore mapping of human chromosomes

GC composition of chromosomal segments was calculated using GC-profile platform^67^. For segmentation of DNA^68^ the halting parameter (t0) of 100 and minimum length of 3000 were selected. The output files were subsequently imported into Circos platform to generate the circular isochore maps of Fig. 4 in the main text.

### Identification of miR_HR_ signature

To identify the miR_HR_ signature, pre-miR-4673 sequence was blasted against the reference library (Homo sapiens all assemblies [GCF_000001405.33 GCF_000306695.2] chromosomes plus unplaced and unlocalized scaffolds in Annotation Release 108). The results were filtered based on defined homology criteria of sequence homology to mir-4673 (Homology_min_ > 68%, no gaps allowed), conserved [5′-GGCTCCTG-3′] consensus sequence in ±strand and [miR4673- miR_HR_]^**Δ**G^ < −36 kcal/mol [miR4673- miR_HR_]^ΔG^ was determined using RNAhybrid platform^69^ (BiBiServ2, Bielefeld University Bioinformatics Server).

### Cytoscape for functional analysis

Cytoscape 3.3.0 was used for gene ontology and functional analysis of miR-4673 interactome genes. ClueGo application was utilised to analyse the identified interactome genes (Fig. 4 in the main text, inner circle).

### Analysis of shadow *gga*-interactome

Shadow interactome of *Gallus gallus* were identified based on the same algorithms, criteria and stringency detailed for human genome. Go analysis of chicken interactome was performed using String 10.0 database^70^, The Gene Ontology Consortium Database^71^, KEGG database^72^ and PANTHER database^73^.

### Ex ovo cultivation of chicken embryos

Fertilised Rhode Island Red eggs were obtained from a local hatchery. The method for ex ovo cultivation of chicken embryos is described elsewhere with slight modifications^74^. Briefly, fertilised eggs were incubated at 37 °C with constant rotation for three days before explantation. After this period the eggs were transferred to a shell-less culture system comprised of transparent plastic cup, clear polyethylene film and rubber bands to fix the film. The eggs were cracked and contents transferred to the shell-less system and covered by a bacterial agar plate. The system was then incubated at a temperature of 37 °C and relative humidity of 70%. Experiments were performed on cultivated embryos at stages HH-16 (51–56 h)^75^.

### Ex ovo electroporation of chorioallantoic membrane

ECM 830 electroporator (Harvard Apparatus®) was used to generate square-wave electric pulses. The solution (20 mM HEPES, 135 mM KCl, 2 mM MgCl2, 0.5% Ficoll 400, 2 mM ATP/5 mM glutathione) containing the pGeneClip-miRNA plasmid (20 ng/μl) was loaded on top of the chorioallantoic membrane and gold-plated Genetrodes (3 mm L-Shape, Harvard Apparatus®) were placed on either side of the folded membrane. Electroporation was carried out at 62.5 V/cm, 50 ms, 2 pulses at 1-s intervals. The electroporated embryos were then incubated for 24 h at 37 °C before capturing the photograph followed by harvesting the membrane for histological processing.

### Ex ovo electroporation of embryos

ECM 830 electroporator (Harvard Apparatus®) was used to generate square-wave electric pulses. The head of the embryo was exposed by cutting the chorionic membrane. A solution (20 mM HEPES, 135 mM KCl, 2 mM MgCl_2_, 0.5% Ficoll 400, 2 mM ATP/5 mM glutathione) containing the pGeneClip-miRNA plasmid (20 ng/μl) was injected into the canal of the neural tube under illumination in a surgical microscope (Leica M320). Platinum Tweezertrodes (5 mm, Harvard Apparatus®) were carefully positioned bilaterally around the embryo’s head and 4 mm apart. Parameters for ex ovo electroporation of chicken embryos were adapted from Sauka–Spengler et al. Electroporation was carried out at 62.5 V/cm, 50 ms, 5 pulses at 1-s intervals. The electroporated embryos were then incubated for 24 h at 37 °C before harvesting for histological processing.

## Supplementary information

Supplementary file

Reporting Sum

## Data Availability

The authors declare that all data generated or analysed during this study are included within the article and its Supplementary information files.
