# Supplementary material for: Genomic competition for noise reduction shaped evolutionary landscape of mir-4673
Source: NPJ Syst Biol Appl. 2020 May 6;6:12. doi: 10.1038/s41540-020-0131-2 (PMC7203229; doi:10.1038/s41540-020-0131-2)
Supplement: Supplementary file 1 — Supplementary file [file 41540_2020_131_MOESM1_ESM.pdf]

Supplementary Table 1. TCF3/4 binding sites in the Intron 4 of Notch-1.

TFs	Score	Normalised score	Position	motif
TCF4	3.484	0.840985041420513	36	AACCCCTGCG
TCF3	4.639	0.847851638642541	114	GGCAGGTGGG
TCF3	11.242	0.958861693088783	114	CCCACCTGCC
TCF4	2.869	0.831579360683257	114	GGCAGGTGGG
TCF4	11.770	0.9677093838415	114	CCCACCTGCC
TCF3	4.661	0.848221504036728	174	GGCAGGTGCC
TCF3	11.284	0.959567799750413	174	GGCACCTGCC
TCF4	3.074	0.834714587595675	174	GGCAGGTGCC
TCF4	11.779	0.96784702794985	174	GGCACCTGCC
TCF3	1.983	0.803198798326136	201	CGCAGCGGTA
TCF3	2.199	0.806830204014519	201	TACCGCTGCG
TCF4	1.384	0.808868082805491	201	CGCAGCGGTA
TCF3	3.373	0.826567566413411	234	CCCAACTGCG
TCF4	1.258	0.80694106528859	234	CGCAGTTGGG
TCF4	2.634	0.827985320076338	234	CCCAACTGCG
TCF3	4.661	0.848221504036728	288	GGCAGGTGCC
TCF3	11.284	0.959567799750413	288	GGCACCTGCC
TCF4	3.074	0.834714587595675	288	GGCAGGTGCC
TCF4	11.779	0.96784702794985	288	GGCACCTGCC
TCF3	2.598	0.813538217300003	335	ACTACCTGGC
TCF4	2.056	0.819145509562298	335	ACTACCTGGC
TCF3	3.469	0.828181524497137	390	ACCCCCTGGG
TCF4	1.620	0.812477417202227	390	ACCCCCTGGG
TCF3	2.915	0.818867641388971	408	GGCGCCTGGT
TCF4	2.582	0.82719004300587	408	GGCGCCTGGT
TCF3	4.891	0.85208827861232	411	TACACCAGGC
TCF4	5.479	0.871496152104784	411	TACACCAGGC

TCF3	11.243	0.958878505152156	548	GGCACCTGGA
TCF3	4.414	0.84406892438381	548	TCCAGGTGCC
TCF4	11.705	0.966715287503416	548	GGCACCTGGA
TCF4	2.393	0.824299516730518	548	TCCAGGTGCC
TCF3	2.242	0.807553122739521	592	TACAGCTCGA
TCF3	2.221	0.807200069408706	691	CGCAGCTCCC
TCF4	1.651	0.812951524686544	691	CGCAGCTCCC
TCF3	2.087	0.804947252916839	740	ACCAGCAGGA
TCF3	6.071	0.871926513391446	754	CACACCCGCA
TCF4	4.418	0.85526944110929	754	CACACCCGCA
TCF3	2.556	0.812832110638373	767	CCTACCTGTC
TCF4	2.853	0.83133466004619	767	CCTACCTGTC
TCF3	2.625	0.813992143011051	777	CGCAGCCGCC
TCF4	1.463	0.810076292201009	777	CGCAGCCGCC
TCF3	3.122	0.822347738507004	866	AGCTGCTGCA
TCF3	3.289	0.825155353090152	866	TGCAGCAGCT
TCF4	3.037	0.834148717372458	866	TGCAGCAGCT
TCF3	2.009	0.803635911973812	869	TAGAGCTGCT
TCF3	2.953	0.819506499797113	869	AGCAGCTCTA
TCF3	4.476	0.845111272312882	930	CACGCCTGTA
TCF4	4.199	0.851920101139437	930	CACGCCTGTA
TCF3	3.725	0.832485412720405	959	CCCACCTCGG
TCF4	2.608	0.827587681541104	959	CCCACCTCGG
TCF3	4.210	0.840639263455893	1064	CGCGCCTGTA
TCF4	4.500	0.856523531874257	1064	CGCGCCTGTA
TCF3	1.997	0.803434167213346	1076	CCCAGCTACT
TCF4	0.901	0.801481182324036	1076	AGTAGCTGGG
TCF3	6.627	0.881274020626356	1239	TGCAGCTGAG
TCF3	6.671	0.88201375141473	1239	CTCAGCTGCA

TCF4	5.774	0.876007820100704	1239	TGCAGCTGAG
TCF4	6.304	0.884113528703543	1239	CTCAGCTGCA
TCF3	4.443	0.844556474221602	1288	CACCCCTGCC
TCF4	4.278	0.853128310534955	1288	CACCCCTGCC
TCF3	3.444	0.827761222912833	1330	GCCACCCGTG
TCF3	3.688	0.831863366375636	1381	GGCCCCTGCA
TCF4	2.775	0.830141744440489	1381	GGCCCCTGCA
TCF3	2.647	0.814362008405238	1415	CTCAGCTGAC
TCF3	2.177	0.806460338620332	1415	GTCAGCTGAG
TCF4	1.996	0.818227882173297	1415	CTCAGCTGAC
TCF3	3.041	0.820985961373861	1429	GCCACCTCTG
TCF3	4.901	0.852256399246042	1551	CCCACCAGCA
TCF4	4.853	0.861922239679544	1551	CCCACCAGCA
TCF3	3.875	0.835007222226226	1563	GGCATCTGAG
TCF4	3.724	0.844655550976516	1563	GGCATCTGAG
TCF3	11.242	0.958861693088783	1651	CCCACCTGCC
TCF3	4.639	0.847851638642541	1651	GGCAGGTGGG
TCF4	11.770	0.9677093838415	1651	CCCACCTGCC
TCF4	2.869	0.831579360683257	1651	GGCAGGTGGG
TCF3	5.375	0.860225317284436	1682	CACAGGTGGC
TCF3	10.535	0.94697556428468	1682	GCCACCTGTG
TCF4	4.503	0.856569413243707	1682	CACAGGTGGC
TCF4	9.616	0.934766560576378	1682	GCCACCTGTG
TCF4	0.988	0.802811742038087	1697	GGCATCAGCT
TCF3	4.151	0.839647351716937	1705	CCCACCCGCC
TCF4	2.626	0.827862969757804	1705	CCCACCCGCC
TCF3	4.166	0.839899532667519	1734	ACCTCCTGGT
TCF4	1.772	0.814802073254362	1734	ACCTCCTGGT
TCF4	2.149	0.820567832015249	1737	CTCACCAGGA

TCF3	3.841	0.834435612071573	1763	CACCCCTGGC
TCF4	4.065	0.849870733304003	1763	CACCCCTGGC
TCF3	4.533	0.846069559925094	1799	TGCCCCTGCA
TCF4	4.046	0.849580151297486	1799	TGCCCCTGCA
TCF3	4.339	0.842808019630899	1849	CCCACCAGCC
TCF4	4.713	0.859781109105209	1849	CCCACCAGCC
TCF3	2.477	0.811503957631974	1942	CACAGCAGGC
TCF4	3.036	0.834133423582642	1942	CACAGCAGGC
TCF4	0.808	0.800058859871085	2019	TGAAGCTGGG
TCF3	3.688	0.831863366375636	2042	GGCCCCTGCA
TCF4	2.775	0.830141744440489	2042	GGCCCCTGCA
TCF3	3.041	0.820985961373861	2076	GCCACCTCTG
TCF3	9.745	0.933694034220689	2105	CACAGCTGTC
TCF3	9.275	0.925792364435783	2105	GACAGCTGTG
TCF4	10.088	0.941985229369849	2105	CACAGCTGTC
TCF4	8.153	0.912391746074579	2105	GACAGCTGTG
TCF3	8.582	0.91414160451889	2259	CCCAGCTGGA
TCF3	8.396	0.911014560731672	2259	TCCAGCTGGG
TCF4	8.581	0.918937488116117	2259	CCCAGCTGGA
TCF4	7.777	0.906641281103508	2259	TCCAGCTGGG
TCF3	2.524	0.812294124610464	2359	GGCCCCTGGC
TCF4	2.422	0.824743036635202	2359	GGCCCCTGGC
TCF3	6.715	0.882753482203104	2368	CCCATCTGCC
TCF4	7.875	0.908140072505543	2368	CCCATCTGCC
TCF4	1.470	0.810183348729725	2400	CACACATGAG
TCF3	2.865	0.818027038220364	2459	GAGACCTGTC
TCF4	2.117	0.820078430741115	2459	GAGACCTGTC
TCF3	5.423	0.861032296326299	2659	AACTCCTGGG
TCF4	3.271	0.837727464189561	2659	AACTCCTGGG

TCF3	4.111	0.838974869182051	2711	CACGCCTGGA
TCF4	4.205	0.852011863878337	2711	CACGCCTGGA
TCF3	11.923	0.970310708245211	2882	CCCACCTGCT
TCF3	6.096	0.87234681497575	2882	AGCAGGTGGG
TCF4	11.929	0.970141096422352	2882	CCCACCTGCT
TCF4	4.010	0.849029574864085	2882	AGCAGGTGGG
TCF3	2.499	0.811873823026161	2945	TGCAGCCGGT
TCF3	4.713	0.849095731332079	2951	CAAACCTGCA
TCF4	4.640	0.858664662448592	2951	CAAACCTGCA
TCF3	4.429	0.844321105334392	3107	CCCACCTCCT
TCF4	2.974	0.833185208614008	3107	CCCACCTCCT
TCF3	2.764	0.816329019819778	3145	GCCATCTGAA
TCF4	1.904	0.816820853510163	3145	GCCATCTGAA
TCF4	1.551	0.811422145704876	3159	TGCAGCTTCC
TCF3	3.560	0.829711422264002	3222	TCCCCCTGCT
TCF4	2.114	0.820032549371665	3222	TCCCCCTGCT
TCF3	3.198	0.823625455323287	3225	AGCAGCAGGG
TCF4	2.543	0.82659358520302	3225	AGCAGCAGGG
TCF3	3.427	0.827475417835507	3280	CCCCCCTGTG
TCF4	2.416	0.824651273896302	3280	CCCCCCTGTG
TCF3	2.305	0.808612282731966	3315	GCCGCCTGCA
TCF4	0.823	0.800288266718335	3315	GCCGCCTGCA
TCF3	12.107	0.973404127905685	3326	TGCACCTGGG
TCF3	5.180	0.856946964926869	3326	CCCAGGTGCA
TCF4	12.844	0.984134914104612	3326	TGCACCTGGG
TCF4	3.205	0.83671807406166	3326	CCCAGGTGCA
TCF3	5.740	0.866361720415268	3332	AGCTCCTGCA
TCF4	3.917	0.847607252411134	3332	AGCTCCTGCA
TCF3	1.853	0.801013230087758	3335	GGCAGCTCCT

TCF3	3.722	0.832434976530288	3568	GGCTCCTGCC
TCF4	2.635	0.828000613866154	3568	GGCTCCTGCC
TCF3	5.120	0.855938241124541	3671	AACAACTGCA
TCF4	3.616	0.843003821676314	3671	AACAACTGCA
TCF3	6.262	0.875137617495525	3710	AACACCTACA
TCF4	4.423	0.855345910058373	3710	AACACCTACA
TCF3	3.947	0.83621769078902	3716	TACAACTGCC
TCF4	3.606	0.842850883778148	3716	TACAACTGCC

Supplementary Table 2

Summary of the contribution of primate-specific transposable elements to miR_{HR} signature.

Chromosome	Gene	T.E.	Family	Homology
2	CYP20A1	AluSq2	SINE1	0.8865
3	OSBPL1A	MER52C	ERV1	0.8083
3	MYRIP	L2	CR1(LINE2)	0.6617
3	KALRN	L2	CR1(LINE2)	0.7478
6	RREB1	L2B	CR1(LINE2)	0.6774
6	MOCS1	LTR33	ERV3	0.6348
7	SPDYE3	LTR45B	ERV3	0.8146
7	WBSCR16	AluJb	SINE1	0.7812
8	XKR6	AluSx	SINE1	0.8564
8	PRKDC	MER76	ERV3	0.7192
8	FAM135B	AluJb	SINE1	0.8169
8	CSMD2	L2	CR1(LINE2)	0.6357
8	MTFR1	AluSz6	SINE1	0.8786
8	LACTB2	AluSx1	SINE1	0.8797
9	SUSD3	MIR	SINE2	0.7619
9	GABBR2	LX5c	L1	0.7564
9	ASTN2	L1PBA_5	L1	0.8182
9	IKBKAP	L2	CR1(LINE2)	0.6514
10	MGMT	MLT1N2	ERV3	0.6705
10	CTNNA3	LTR12E	ERV1	0.85
10	CACNB2	AluJo	SINE1	0.7872
11	ARRB1	L2B	CR1(LINE2)	0.8267
11	PPFIA1	AluJb	SINE1	0.7751
11	IGHMBP2	MER52C	ERV1	0.8444
11	CKAP5	AluJb	SINE1	0.8611
13	FAM155A	MSTB	ERV3	0.8515
13	PCCA	AluSx	SINE1	0.8841

13	CLDN10	AluSz6	SINE1	0.8808
14	UNC79	LTR7A	ERV3	0.872
14	AKAP6	MER11A	ERV2	0.8929
14	CATSPERB	LTR1B	ERV1	0.8158
14	RGS6	MER115	DNA/hAT	0.7372
14	FERMT2	L2	CR1(LINE2)	0.7164
16	CENPN	AluSz	SINE1	0.8387
16	PHLPP2	LTR7A	ERV3	0.8277
16	LCMT1	L2	CR1(LINE2)	0.6948
16	C16ORF89	LTR16A1	ERV3	0.7004
17	MYOCD	Charlie19a	DNA/hAT	0.6564
17	GAS7	MLT1K	ERV3	0.6814
19	PRR12	AluJb	SINE1	0.7857
19	ATP13A1	AluJb	SINE1	0.7325
19	OLFM2	MIR	SINE2	0.7917
19	CCDC97	L2B	CR1(LINE2)	0.7143
20	TMEM189	L1-2_TS	L1	0.7266
X	ATP11C	L2	CR1(LINE2)	0.6385
X	IL1RAPL1	AluSq	SINE1	0.8767
X	NHS	MLT1J2	ERV3	0.8018
X	RBBP7	AluJb	SINE1	0.8627
X	TEX11	L1P4d_5end	L1	0.7954

Supplementary Table 3. Primers designed for nascent RNA-based fingerprinting of enhancer activity.

NPS Primers	Sequence	M.T.	Biotype
eRNA1-F	5'-GCCTGACACCCCTGGACAA-3'	60 ºC	Exonic (Ex.3)
eRNA1-R	5'-GCACGATTTCCCTGACCAGC-3'	58.9 ºC	Exonic (Ex.3-4)
eRNA2-F	5'-CACAACGAGGTCGGCTCCTAC-3'	59.7 ºC	Exonic (Ex.4)
eRNA2-R	5'-CAGGCACACTCGTGGGTGA-3'	60 ºC	Exonic (Ex.4)
eRNA3-F	5'-CAGCCCGTGTCTCTGGAGTT-3'	59.3 ºC	Intronic (Int.4)
eRNA3-R	5'-GCTTGGGAAGTGATGGTGCC-3'	59 ºC	Intronic (Int.4)
eRNA4-F	5'-TGTTAGGTGGGAATGGGGAGAGG-3'	60.3 ºC	Intronic (Int.4)
eRNA4-R	5'-GGGAGCTTCCCCTTTGCAGG-3'	61 ºC	Intronic (Int.4)
eRNA5-F	5'-CCCTACCTCCAGTCACACGGA-3'	60.5 ºC	Intronic (Int.4)
eRNA5-R	5'-TGGGCATCAGCTCTGCACAC-3'	60.5 ºC	Intronic (Int.4)
eRNA6-F	5'-GAGTGTGGGTAGATCTCAGAGGCT-3'	59.5 ºC	Intronic (Int.4)
eRNA6-R	5'-GGGACAGTCTGAAGCTGGGGT-3'	61.4 ºC	Intronic (Int.4)
eRNA7-F	5'-CACTGTGTGCCTCAGCCAGT-3'	60.1 ºC	Intronic (Int.4)
eRNA7-R	5'-CCGGTCCCACTCATGTGTGG-3'	60.5 ºC	Intronic (Int.4)
eRNA8-F	5'-GGGTTCAAAGGCATCTCGGGA-3'	59.6 ºC	Intronic (Int.4)
eRNA8-R	5'-CCTCCAAGGCAGCCAGTTAGC-3'	60.6 ºC	Intronic (Int.4)
eRNA9-F	5'-CCTGTGGAGACATTGTAGGGAGC-3'	59.2 ºC	Intronic (Int.4)
eRNA9-R	5'-GGACAGGACTGGCTAAACAGGG-3'	59.6 ºC	Intronic (Int.4)
eRNA10-F	5'-GCCAGAGTGGACAGGTCAGTACT-3'	60.2 ºC	Exonic (Ex.5-6)
eRNA10-R	5'-GACACACACGCAGTTGTAGCCAC-3'	60.6 ºC	Exonic (Ex.6)

Supplementary Table 4. Primers designed for temporal fingerprinting of miR-4673 interactome.

Gene	Primer	Sequence
MTHFR	Forward<br>Reverse	AAGGAGGAGCTGCTGAAGATGTGG<br>GCAGGCAAGTCACTTTGTGACCA
FBXO6	Forward<br>Reverse	GCCACATGGACAGAGGTCTCCT<br>CCATACCAGCCTGCCCAGTAC
E2F2	Forward<br>Reverse	ACAGGACTGAGGACAACCTGCA<br>GAGAGGCTCCTCGGAAGGACT
ADAMTSL4	Forward<br>Reverse	GGGAACCTCACTGACCGAGG<br>CGAAGTGCCAGGTAGTTGGAGC
CRB1	Forward<br>Reverse	CGAGGTGGACTTGGCAGATGAC<br>GGAGGTGACAACAGAAGCAACAATGG
CDK18	Forward<br>Reverse	CCGAGAGGTGTCTCTGCTGAAG<br>CAGGTACTCAAACACCAGGGTGAG
TP53I3	Forward<br>Reverse	GCGGACTTAATGCAGAGACAAGGC<br>GCTGTGTCCCCGATCTTCCAG
COL4A3	Forward<br>Reverse	CCACGGACAAGACCTTGGAACTC<br>AGCTGGTGTTGACAGCCAGTATGA
ATP2B2	Forward<br>Reverse	GCAGCTCTTCAACGAGATCAACGC<br>CTGCACGATCACTATCTGGATGGC
TGBR2	Forward<br>Reverse	AGCCTCCATTTGGTTCCAAGGTG<br>CATCTGGATGCCCTGGTGGT
GATA2	Forward<br>Reverse	GGCTCGTTCCTGTTCAGAAGGC<br>GCTTGATGAGTGGTCGGTTCTGC
AMOTL2	Forward<br>Reverse	CTGTGTCTTGCTTGCCAGGC<br>CTGCTGTTCGTAGCTCTGAGCAAG
CCHCR1	Forward<br>Reverse	GCCTTGAGTTGCAGCAGTTGC<br>CTCTGCCTCCCCTTGCTCC
RNF216	Forward<br>Reverse	GGACCGATCCCACTGAAGATGATG<br>CTTCTCCACAGGCTTCTCCAGC
CLIP2	Forward<br>Reverse	TCAAGGCACAGCATGAGCAGTATG<br>TCCTCCACCTTCCTCCTCTCC
MFHAS1	Forward<br>Reverse	TCCAGGGGAGTTGCTGAGTCA<br>GTGGGTGGGTAAACCAAGGCAA
XPO7	Forward<br>Reverse	GCAGTTCATGCTGCCACTCAC<br>CTAGAGTTCGCTTTGCCTCCTGC
DENND1A	Forward<br>Reverse	GAGTACGCTGGCAGTGACAAACTG<br>GCGAACTTGTAGACAGTCTTCATGGC
CACFD1	Forward<br>Reverse	AGGCTGGTTCCTTCGCAGC<br>GGAGATCGCATCGCCCCTG
NOTCH1	Forward<br>Reverse	GACGCCACTGATCCTGGCT<br>CGGACTTGCCCAGGTCATCTAC
RABL6	Forward<br>Reverse	GGAGACAGGAACACGGGCAAG<br>CATCCGTGGTCTTGTAGCTCCAG
RET	Forward<br>Reverse	AAGAGGAGCCAGGGTCGGAT<br>ACGATCTCCCACAGCAGGACA
SLIT1	Forward<br>Reverse	GAGATTGCACCCGACGCCTT<br>GGCATTCAGGAGCAGGAGCTG
TCERG1L	Forward<br>Reverse	CCTGTCTGTCTGGGAGAAGCC<br>TCGCTGTTGTCAGTTGCTGGTG
AMPD3	Forward<br>Reverse	CAGTTCCACTACACGAAGGAAGCAC<br>CACGCTGTTCCTGGCGATCT
NAV2	Forward<br>Reverse	GCTCTTCCTGTCATGCCCCATC<br>GCCTTCCATAGAGCTGGAGTCC
SPI1	Forward<br>Reverse	CCATCAGAAGACCTGGTGCCCTA<br>CCAGTAATGGTCGCTATGGCTCTC

CDC42BPG	Forward<br>Reverse	GAGACGCAGACCCCATGAAGAG<br>GCCGTTCTGAGACCTGCATTAGG
FRMD8	Forward<br>Reverse	GCTCCTCACTGGCTCCTGTT<br>CTTGCCGTCCTCCACGTAGT
COL4A1	Forward<br>Reverse	CCAGCAAGGTGTTACAGGATTGGT<br>GGCAGGTCCCATCTCTCCTTTC
HHIPL1	Forward<br>Reverse	GGTTGGCAAGTCGGTCACAGG<br>GACATCAGACGCCCGCTCAT
DLK1	Forward<br>Reverse	GACAATGTTTGCAGGTGCCAGC<br>GGGTTCTCCACAGAGTCC
XRCC3	Forward<br>Reverse	GCATCAACCAGGTGACAGAGGC<br>GGTCAGCCAGCAGTCTCACC
NIPA2	Forward<br>Reverse	CTCAGCGTGCTAGTAAGTGCCATTC<br>GTCTCAATCTCCTCTTCCTTTGGAGCA
ATXN1L	Forward<br>Reverse	GCACCACTGGAAACTCAGATGTGG<br>TTCCTGGAGCCTGGAAGGAGTC
QRICH2	Forward<br>Reverse	TGCCATGAAGCATGATGAGGTGG<br>GCTTTGAGGTCCCTGAGCTGTC
P4HB	Forward<br>Reverse	TGCCGACAGGACGGTCATTG<br>GAGATCGTCATCATCCCCTGCC
SHC2	Forward<br>Reverse	CCACCTGATCGACCACCAC<br>GAGAACGGTCACCTGGCTCAG
UBE2M	Forward<br>Reverse	GTCATCTGTCCTGATGAGGGCTTC<br>CTCTGAGGATGTTGAGGCAGACG
KREMEN1	Forward<br>Reverse	CCCACCTCTGTCCTTCAACGTC<br>CAGTTCTTCCTTGACGGCTTGGT
PPARA	Forward<br>Reverse	TTGCTGTGGAGATCGTCCTGG<br>GCAGGTGGAGTCTGAGCACA
SATL1	Forward<br>Reverse	TGGCTGCCTGTGAAAACATGCTAG<br>TCCAACAGTCAGTTTGCCTGATGG

Supplementary Discussion 1:

A functional appraisal of the miR$_{HR}$-bearing Wnt modulator genes.

Targets identified as miR-4673 interactome are involved in regulation of the canonical and non-canonical Wnt cascades [1] as follows.

SDC-3 (syndecan-3) can calibrate catenin-β1 by two direct and indirect mechanisms as follows. Cell surface syndecans concentrate and enhance FGF signalling [2]. Fgf signalling inhibits autophagy [3] via PI3k/Akt cascade and activation of mechanistic target of rapamycin (mTOR). Functional autophagy can effectively decrease the level of free cytoplasmic catenin-β1 [4]. Activation of PI3K also contributes to increased cytosolic calcium level by increasing membrane-bound voltage-gated calcium channels[5] and also activating IP3/DAG cascade [6]. Syndecans also enhance membrane representation of transient receptor potential canonical channels and subsequently, the level of intracellular calcium [7]. In a dose-dependent manner, increased cytosolic Ca^{2+} propels disassembly of Calmodulin/IQGAP1 complex to facilitate interaction of IQGAP1 with CDC42 [8]. Calcium influx also activates Lis1 that in turn promotes activation of Cdc42 through interaction with IQGAP1 [9]. The net result is as stated above; catenin-β1 can bind to cadherin in adherence junctions [10] leading to reduction of the level of free cytoplasmic catenin-β1. Inhibition of IQGAP1/catenin-β1 association is known to reduce nuclear localization of catenin-β1 via importin-β5 following active Wnt signalling [11]. On the other hand, inhibitory phosphorylation of GSK-3 by Ca^{2+}/calmodulin-dependent protein kinase II (CaMKII) [12] counterbalances the previous activity by replacing the cytosolic catenin-β1. Further, syndecan co-localizes with E-cadherin, catenin-β1 and syntenin at cell-cell contacts [13] enforced by availability of cytoplasmic calcium and hence reduces free cytoplasmic catenin-β1.

ESPN (Espin) cross-links and stabilizes the actin cytoskeleton in a radial arrangement [15, 16] which in turn bolsters organisation of adherence junctions [17]. Adherence junctions recruit catenin-β1 [18, 19] and thus reduce the available pool of free cytoplasmic catenin-β1. Actin cytoskeleton also stimulates calcium flux from L-type calcium channels [20]. As stated above, increased cytosolic Ca^{2+} propels disassembly of Calmodulin/IQGAP1 complex to facilitate interaction of IQGAP1 with CDC42. Calcium influx also activates Lis1 that in turn promotes activation of Cdc42 through interaction with IQGAP1. As a consequence, association of catenin-β1 with adherence junctions is reinforced to reduce the available cytoplasmic pool of the protein.

Kazrin partners with p120 family members (ARVCF-, delta-, and p0071-catenin) [21] to stabilize ARVCF [22] and enhance signalling by Rac GTPase [22, 23] (see below). Enhanced Rac/CDC42 signalling inhibits binding of IQGAP1 to catenin-β1 [10] with two important consequences; catenin-β1 binds to cadherin in adherence junctions [10] reducing the level of free cytoplasmic catenin-β1. Also, inhibition of IQGAP1/catenin-β1 association reduces nuclear localization of catenin-β1 via importin-β5 following active Wnt signalling [11].

CHD5 positively regulates p53 cascade [24]. Activated p53 cascade increases degradation of catenin-β1 [25] by stabilizing the association this protein with axin1 leading to subsequent phosphorylation and degradation of catenin-β1 [26].

LAPTM5 (Lysosomal-Associated Multi-transmembrane Protein 5) is a retinoic acid-inducible [27] protein that in partnership with protein ligase Nedd4 regulates cargo trafficking to the lysosome [28]. Nedd-4 in combination with Rac1 mediates ubiquitination and degradation of the adaptor protein dishevelled-1 (Dvl1) [29]. Dvl1 is central to stabilization of catenin-β1 downstream of Wnt signalling [30]. HECT-containing Nedd4-like ubiquitin E3 ligase also ubiquitinates the phosphorylated form of Dvl1 leading to its degradation [31]. Therefore, LAPTM5 functions as an adaptor that facilitates ubiquitination of Dvl1 en route to the final endosome.

CEP85 binds to Nek2 (NIMA Related Kinase-2), upon which the kinase activity of Nek2 is abrogated to prevent centrosome disjunction during interphase [32]. In M phase of the cell cycle, phosphorylation by Nek2 stabilizes and prevents ubiquitination and degradation of catenin-β1 [33] in alignment with Wnt-mediated programming of mitotic spindle orientation and subsequent cell fate [34].

GABRD Tonic GABA-A receptor signalling increases cytosolic Ca^{2+} level through voltage-gated calcium channels [35]. In a dose-dependent manner, increased cytosolic Ca^{2+} propels disassembly of calmodulin/IQGAP1 complex to facilitate interaction of IQGAP1 with CDC42 [8]. Calcium influx also activates Lis1 that in turn promotes activation of Cdc42 through interaction with IQGAP1 [9]. The net result is as stated above; catenin-β1 can bind to cadherin in adherence junctions [10] leading to reduction of the level of free cytoplasmic catenin-β1. Also, inhibition of IQGAP1/catenin-β1 association reduces nuclear localization of catenin-β1 via importin-β5 following active Wnt signalling [11]. On the other hand, inhibitory phosphorylation of GSK-3 by Ca^{2+}/calmodulin-dependent protein kinase II (CaMKII) [12] counterbalances the previous activity by replenishing the cytosolic catenin-β1.

CYP4X1 is a monooxygenase that catalyse olefin epoxidation (epoxygenase reaction) of arachidonic acid that generates epoxyeicosatrienoic acids [36]. 5,6-epoxyeicosatrienoic acid mediates [37] capacitative entry of calcium [38] into cells. The cascade of events after enhanced concentration of cytosolic Ca^{2+} is as described for GABRD above.

PRDM16 (MEL1) is a potent inhibitor of smad3 downstream of the TGF-β signalling cascade [39, 40]. TGF-β signalling induces rapid nuclear translocation of catenin-β1 via smad-3 [41]. Inhibition by PRDM16 of smad-3 significantly reduces shuttling of cytoplasmic catenin-β1 into the nucleus.

PLEKHG5 (alias: Syx) activates RhoA and promotes selective coupling to Dia1 [42]. ERBB2 (Her2) signals via an assembled Memo-RhoA-mDia1 complex to inhibit GSK3β locally to

increase the concentration of catenin-β1 and also to relocate APC, CLASP2 and ACF7 to the cell membrane to stabilize microtubules [43].

EphB2 (EphrinB2) can activate β-catenin independently of Wnt signalling [44]. Wnt signalling activates the small GTPase Rho. This activation follows the assembly of formin homology protein Daam1, dishevelled and Rho [45]. Dvl forms a Wnt-induced complex with Rac independent of the Wnt-induced Dvl–Rho complex [46]. Eph receptors and ephrin-B ligands complex with dishevelled and RhoA/Rho kinase to regulate forward and reverse ephrin signalling and cell sorting [39, 47, 48].

Col16A1 is a member of the FACIT collagen family (fibril-associated collagens with interrupted helices). It binds to cell surface integrin-α1β1 [49] in focal adhesions that in turn activate integrin-linked kinase [50]. Integrin-linked kinase inhibits GSK3β by phosphorylation of serin-9 [51]. Inhibition of GSK3β increases the level of free cytoplasmic catenin-β1. This effect is partially mediated by recruitment of IQGAP1 and dia1 to locally stabilize microtubules and inhibit GSK3β [52].

NID1: Nidogen-1 also binds to integrin-α3β1 to activate downstream cascades as per Col16A1 [53].

Col4A1/Col4A3/Col4A2/Col4A1/Col15A1/Col1A1/LAMA5/LAMA3: The same role as NID1 in engaging integrins.

ADAMTSL4 accelerates microfibril formation [54] and assembly of basement membrane that in turn activates the integrin-linked cascade mentioned above.

HAPLN2 strengthens the ECM formed by hyaluronic acid [55] and amplifies outside-in signalling by integrins through integrin-linked kinase [56].

RASAL2 activates Rac1 [57]. Rac1 activation is required for phosphorylation of Ser191 and Ser605 of catenin-β1 to propel its nuclear shuttling [58]. Rac1 at the same time stimulates incorporation of catenin-β1 into the junctional complexes at the cell membrane [59] by activating the Wave complex [60] and stabilizing the actin cytoskeleton [61].

E2F2 transcription factors antagonize Wnt activity by inhibiting transcription of catenin-β1 [62]. Further, they activate the transcription of β-catenin interacting protein 1 [63] that in turn interferes with Wnt/catenin-β1 signalling.

SOX13 The SOX family of transcription factors are central to modulation of β-catenin and TCF transcription factors [64]. SOX13 antagonizes Wnt signalling by competing for the catenin-β1-binding domain of TCF1 [65].

TDRD5 is required for silencing transposable elements [66] that are mobilized following Wnt signalling via TCF-based promoters [67].

SH2D2A (alias: Tsad) activates c-Src kinase that in turn phosphorylates IQGAP1 [68, 69]. Phosphorylated IQGAP1 binds preferentially to CDC42 [70] and releases catenin-β1 to associate with adherence junctions. However, Src kinase weakens adherence junctions by downregulating cadherins [71] while increasing the transcription [71] and translation [72] of catenin-β1. The net result is an increased cytoplasmic pool of free catenin-β1.

MTHFR catalyses the conversion of 5,10-methylenetetrahydrofolate to 5-methyltetrahydrofolate. Folate antagonizes LRP6, a co-receptor of Wnt, and calibrates intracellular catenin-β1 [73, 74]. Further, depletion of homocysteine as a result of conversion to methionine downstream of MTHFR catalytic activity, reduces Ca^{2+} influx by homocysteine-dependent overstimulation of N-methyl-D-aspartate receptors [75].

CRB1: crumbs homologue-1 is involved in the formation and maintenance of adherence junctions [76-78] and hence recruitment of intracellular catenin-β1 to these junctions.

PLB1 is a phospholipase A2 that hydrolyzes the sn-2ester bond of membrane phospholipids and generates arachidonic acid [79]. Arachidonic acid activates mammalian target of rapamycin (mTOR) signalling pathway (both mTORC1 and mTORC2) [80] reducing autophagy-dependent reduction of free cytoplasmic catenin-β1. Further, arachidonic acid activates CDC42 leading to recruitment of free cytoplasmic catenin-β1 in junctional complexes [81]. Arachidonic acid also activates calcium entry into the cytoplasm [82-84] triggering calcium-dependent enhancement of Wnt signalling.

TP53I3 amplifies p53 signalling [85]. Activated p53 cascade increases degradation of catenin-β1[25] by stabilizing axin/catenin-β1 assembly with subsequent phosphorylation and degradation of catenin-β1 [26].

CFC1, CFC1B: Cripto1 enhances binding of Wnt3a to the co-receptors low-density lipoprotein receptor-related protein-5 (LRP5) and LRP6 with consequent stabilization of cytoplasmic catenin-β1 [86].

CYP20A1 is a monooxygenase that catalyse olefin epoxidation (epoxygenase reaction) of arachidonic acid generating epoxyeicosatrienoic acids [36]. 5,6-epoxyeicosatrienoic acid mediates [37] capacitative entry of calcium [38] into cells. The cascade of events after enhanced concentration of cytosolic Ca^{2+} is as described for GABRD above.

SPHKAP: Sphyngosine-1-phosphate generate by activity of SPHKAP is an activator of cdc42 [87]. Sphyngosine-1-phosphateincreases intracellular Ca^{2+} by muscarinic acetylcholine receptors[88-90] in response to IP3-induced activation of SPHKAP [90].

CNGA3: Activation of cyclic nucleotide-gated channels leads to depolarization of the membrane voltage concomitant with increased Ca^{2+}influx [91].

STK39: Wnk kinases, as positive regulators of Wnt signalling [92], stimulate SPAK and OSR1 kinases that phosphorylate and stimulate Cl^- -importing or inhibit Cl^--extruding co-transporters [93]. Chloride influx hyperpolarizes the cell membrane and transiently inhibits voltage-gated calcium channels [94].

SFXN5 is a brain-specific mitochondrial citrate transporter [95]. Acetyl-CoA is exported in the form of citrate that in turn activates lipogenesis and synthesis of cholesterol [96, 97]. Enrichment of cholesterol in the cell membrane around Wnt-activated Frizzled and low-density lipoprotein receptor-related protein 5/6 receptors facilitates canonical Wnt signalling [98].

TPO is involved in generation of thyroxine and triiodothyronine which act ssynergistically with Wnt signalling [99]. Also thyroid hormones enhance the activity of 3-hydroxy-3-methylglutaryl-coenzyme-Areductase as the first step in cholesterol biosynthesis [100].

TRAPPC12 is involved in regulation of kinetochore stability and CENP-E recruitment [101]. This is aligned to the activity of catenin-β1 during mitosis to regulate mitotic spindle orientation and subsequent tissue architecture [102].

CNTN4 (alias BIG-2) forms a scaffold for the assembly of PKA with other substrates [103] such as catenin-β1. Interaction with catenin-β1 enhances stability and activity of the latter protein [104].

SEC13 is a functional component of GATOR2 complex [105]. Release of GATOR2-mediated inhibition of GAPTOR1 leads to mTORC1 suppression [106] with resultant amplification of autophagy and degradation of catenin-β1 [4].

OSBPL1A acts as a cholesterol sensor [107, 108] and in low-cholesterol conditions induces the formation of endoplasmic reticulum (ER)-LE membrane contact sites where p150 and associated motor apparatus is removed and the vesicle moves towards microtubule plus ends [107] that are in contact with adherence junctions [109]. This is critical to transfer cadherin/catenin-β1 complex to adherence junctions [110]. Rab7, the receptor of OSBPL1A on late endosomes/lysosomes, acts as an inhibitor of mTORC1 by suppressing S6K phosphorylation [111]. Exclusion of Rab7 from

endo-phagosomes will decrease autophagy and degradation of catenin-β1 to support the formation of junctional complexes.

ULK4 is involved in regulation of cortical neurogenesis [112] by modulating Wnt signalling [113]. Involvement of Ulk proteins in inhibition of autophagy may explain the latter phenomenon [114].

MYRIP belongs to protein kinase A-anchoring family of proteins and anchors PKA, involved in phosphorylation and stabilization of catenin-β1 [104], into specific subcellular compartments [115].

CACNA2D2 acts as a regulatory subunit of multiple voltage-dependent calcium channel complexes by amplifying the response of these channels to voltage change [116].

MAPKAPK3 acts downstream of p38 mitogen-activated protein kinasewhich stabilizes catenin-β1 by inhibiting GSK3β [117].

ARPP21: Phosphorylation by PKA of this protein (alias: RCS) enhances its affinity to calmodulin and hence competitive inhibition of calmodulin-dependent signalling [118]. As a consequence inhibitory phosphorylation of GSK-3β by Ca^{2+}/calmodulin-dependent protein kinase II (CaMKII) [12] will decrease leading to destabilized free cytoplasmic catenin-β1.

ANO10 forms a Ca^{2+}-activated chloride channel [119] that is also modulated by calmodulin [120]. Chloride influx leads to hyperpolarization of the cell membrane and transient inactivation of voltage-gated calcium channels.

ATP2B2 (alias: PMCA2) is a calcium transporter that decreases cytosolic Ca^{2+}concentration [121, 122]. Further, PMCA2 inhibits Her2 (ERBB2)[121] that acts via Memo-RhoA-mDia1 complex to inhibit GSK3β locally and increase the concentration of catenin-β1 [43].

TGFBR2: TGF-β signalling induces rapid nuclear translocation of catenin-β1 via smad-3 [41].

GATA2 inhibits canonical Wnt signalling [123].

AMOTL2 acts as an inhibitor of Wnt signalling by sequestering catenin-β1 in the Rab11-positive recycling endosomes [124].

PARK2: Parkin induces catenin-β1 ubiquitination and degradation [125].

MYO6 stabilizes association of E-cadherin and catenin-β1 [126].

CCHCR1 localizes to the centrosome and also involved in the regulation of cytoskeleton [127]. Both these activities are central to signalling by catenin-β1 as explained before.

LAT2 (alias: NTAL): Upon phosphorylation NTAL recruits the Grb2 linker that relieves inhibition of Ca^{2+} influx from extracellular milieu [128]. It is noteworthy that Grb2 also acts synergistically with Wnt cascade and integrin-dependent focal adhesion kinase [129].

SPDYE3 binds to and activates CDK2 [130] that in turn phosphorylates and promotes rapid degradation of catenin-β1 [131].

CLIP2 (alias: clip-115) is required for modulation of microtubule plus end [132, 133]. This effect is partially mediated by partnering with GSK3β [134].

DLGAP2 co-localizes withPSD-95, NMDAR and APC [135]. The assembly is central to clustering and activity of NMDAR and calcium influx [136].

LGI3 associates with and modulates, the activity of syntaxin-1 [137] that is required for delaying activation of calcium-activated potassium channel [138].

TRIB1 enhances MEK1 binding and ERK phosphorylation [139]. ERK associates with and inactivates GSK3-β by phosphorylation [140]. Independent of the latter, MEK1/ERK and Wnt cascades act synergistically [141].

SGK223 (alias: Pragmin) binds to Rnd2 (Rho Family GTPase 2) and stimulates RhoA activity [142]. RhoA activity inhibits Wnt signalling cascade [143]. At the same time, as part of Memo-RhoA-mDia1 complex inhibit GSK3β locally to increase the concentration of catenin-β1 close to junctional complexes for further sequestration.

MTFR1 regulates mitochondrial respiration and hence generation of reactive oxygen species. Calcium-dependent mitochondrial production of reactive oxygen species modulates amplitude of Wnt signalling output [144]. Thioredoxin maintains an inactive pool of Dvl and upon redox-dependent dissociation of thioredoxin and Dvl, Wnt signalling can stabilize catenin-β1 [145, 146].

LACTB2 is an endoribonuclease that is essential for turnover of mitochondrial RNA and functionality of mitochondria [147]. Calcium-dependent mitochondrial production of reactive oxygen species modulates the extent of the Wnt signalling output [144].

FANCC acts as transcriptional repressor of DKK1 (Wnt antagonist) [148]. FANCC also forms a complex with catenin-β1 and facilitates its nuclear shuttling [148]. FANCC also binds to STAT-1 and facilitates activation of the latter protein [149]. JAK-STAT pathway acts synergistically with PI3k and MAPK pathways [150, 151].

SUSD3 enhances cytoskeletal organisation downstream of estrogen receptor-α [152] by increasing the activity of Rho and FAK. Enhanced FAK activity stabilizes catenin-β1 by phosphorylating GSK3β [153].

GABBR2 increases intracellular calcium by coupling with calcium channel, voltage-dependent, L type, alpha 1D subunit [154].

RALGPS1 interacts with and activates RalA [155]. RalA in turn is a key determinant of integrin-dependent lipid raft trafficking to plasma membrane [156]. Lipid rafts are essential for Wnt signalling through interaction with co-receptor LRP6 [157, 158].

PPAPDC3: This nuclear envelope protein (alias: NET39) negatively regulates mTOR activity [159] with resultant increase in autophagy and degradation of catenin-β1.

KCNT1 is a calcium-activated potassium channel [160].

RAPGEF1 (alias: C3G) binds to CRK-like proto-oncogene (crkl) [161, 162] and activates Raf/ERK pathway [161] to fine-tune Wnt signalling by inactivating GSK3β [117].

DENND1A regulates adherence junction formation [163] that in turn titrates catenin-β1 as explained before.

ASTN2: In combination with frizzled-6, a negative regulator of Wnt signalling [164], ASTN2 regulates planar cell polarity [165].

IKBKAP combines with filamin-A to modulate cytoskeletal remodelling, cell adhesion and migration [166].

Notch1 antagonizes Wnt signalling by interaction at various levels [167-169].

RABL6 (alias: RBEL1) inhibits Rb-1 [170] that in turn disrupts Axin/GSK3β/catenin-β1 complex leading to increased availability of catenin-β1[171].

CACFD1 is involved in regulation of intracellular calcium level [172] that in turn modulated catenin-β1 as explained before.

RET binds to and phosphorylates catenin-β1 that in turn stabilizes the latter [173].

GFRA1: GDNF Family Receptor Alpha 1 activates Ret [174] that in turn stabilizes catenin-β1 [173].

SLIT1 in combination with Robo and Abl tyrosine kinase, SLIT1 triggers phosphorylation of catenin-β1 leading to dissociation from N-cadherin and shuttling into the nucleus [175].

CTNNA3 antagonizes Wnt signalling by inhibiting assembly of TCF and catenin-β1 [176].

CACNB2: The regulatory subunit of high voltage-activated calcium channels that slows calcium current inactivation [177].

AFAP1L2 (alias: XB130) links RET/PTC signalling to PI3-kinase activation and up-regulation of Akt cascade. Akt phosphorylates and inactivates GSK3β leading to increased cytosolic catenin-β1 [178].

ARRB1: β-arrestin is essential for Wnt signalling by linking Dvl and axin1 [179]. Further, it is required for phosphorylation and activity of LRP6.

LRP5: the Wnt co-receptor [180].

DKK3 inhibits Wnt signalling pathway by binding to LRP5/6 [181, 182].

PTPN5 inactivates MAPK[183] that in turn inhibits GSK3β by phosphorylation [117].

AXIN1 is a negative regulator of Wnt cascade by destabilizing catenin-β1 [184, 185].

ITSN1 functions as guanine nucleotide exchange factor (GEF) for Cdc42 and accelerates actin assembly via N-WASP and the Arp2/3 complex [186].

TYRO3 activates the PI3K cascade [187]. Interaction with Wnt occurs via Akt phosphorylation of GSK3β and TSC1/2-dependent modulation of mTOR signalling and inhibition of autophagy.

CTNND2 enhances Wnt signalling by increasing translocation of catenin-β1 into the nucleus [188].

WHSC1: a histone methyl-transferase involved in regulating the Wnt pathway [189].

NHS: Nance-Horan syndrome protein contains a Wave domain and is involved in the regulation of cell-cell contact and focal adhesions [190, 191].

IL1RAPL1 associates with NCS-1 and inhibits N-type voltage-gated calcium channels (N-VGCC) [192].

CASK: Wnt signalling stimulates Ca2+/calmodulin-dependent protein kinase II (CamKII) [193] and IQGAP1-linked cell junction-associated[194] CASK suppresses CaMKII activity [195] downstream of the Wnt pathway. CaMKII inhibits GSK3β by phosphorylation [12].

PRODH is a proline oxidase that is induced by p53 and down-regulates the Wnt pathway by hyper-phosphorylation of catenin-β1 [196].

EMID1 enforces the extracellular matrix [197] and the subsequent events are as explained for collagens and laminins.

KREMEN1 is a high-affinity Dkk1 receptor that associates with Dkk1 and LRP6 to antagonize the Wnt cascade by triggering rapid endocytosis of LRP6 [198, 199].

TMEM189 decreases Hrs-dependent endosomal trafficking [200] essential for Wnt signalling [201].

APCDD1L: The closest paralogue of this gene, APCDD1, antagonizes Wnt signalling by associating with catenin-β1 [202, 203].

CDH4: Calcium-dependent cell adhesion protein that in combination with cadherin-2 sequesters cytoplasmic catenin-β1 [204].

PIP5K1C catalyses conversion of phosphatidylinositol 4-phosphate to phosphatidylinositol 4,5-bisphosphate that in turn recruits clathrin-AP2 to LRP6 [205] to form a Wnt signalosome [30].

BEST1 is a calcium-activated chloride channel [206].

CHKA is required for the biosynthesis of phosphatidylcholine [130]. Inhibition of this enzyme reduces MAPK and PI3K/AKT signalling by depleting the cytoplasmic pool of phosphatidyl choline [207].

PPFIA1 (alias: liprin-α1) is essential for stabilization of surface integrins [208, 209] and is regulated by calcium/calmodulin-dependent protein kinase II [210].

CKAP5 is required for stabilization of a centrosome [211].

NELL1 enhances Wnt signalling by stabilizing catenin-β1 via an integrin-mediated mechanism [212].

TFDP1 promotes Wnt signalling in a biphasic noiseless manner [213]. Prior to Wnt signalling it acts by inhibiting Dvl-axin interaction that enhances ubiquitination of β-catenin. Upon Wnt stimulation, it acts via increasing cytosolic β-catenin and antagonizing the kinase activity of NLK.

CYP46A1 is involved in conversion of cholesterol to 24S-hydroxycholesterol [214]. Cholesterol density in the cell membrane around Wnt-activated Frizzled and low-density lipoprotein receptor-related protein 5/6 receptors facilitates canonical Wnt signalling [98].

UNC79 a regulatory component of NALCN channel that is responsible for Na+ leak current [215] and hence determines the membrane potential and excitability of voltage-gated calcium channels [216].

PRIMA1 anchors acetylcholinesterase into the membrane [217].

CATSPERB encodes alkalinization-activated Ca^{2+}-selective channel [218].

WWOX acts as an inhibitor of Wnt signalling by sequestering Dvl proteins [219].

LCMT1 is essential for carboxyl methylation of the PP2A catalytic subunit and formation of the holo-enzyme [220]. While the holo-enzyme reduces Wnt signalling [221] partially by activating GSK-3β [222], PP2A catalytic subunit increases Wnt signalling [223].

ADORA2B: Adenosine receptor that amplifies increased cytosolic calcium level by an IP3-independent mechanism [224-226]. It is also involved in activation of MAPK and modulation of cAMP production or phospholipase C activity [227].

WRAP53 increases the level of p53 level by acting as an endogenous antisense RNA [228]. Activated p53 cascade increases degradation of catenin-β1 [25] by stabilizing association of the latter with axin and hence phosphorylation and degradation of catenin-β1 [26].

SPNS2: This transporter controls the level of sphingosine-1-phosphate [229], which is shown to enhance catenin-β1 level byPI3K/Akt-mediated inhibition of GSK3β [230].

WIPI1 acts downstream of Ca^{2+}/calmodulin-dependent kinase and enhances autophagy [231].

CAMTA1 binds to calmodulin and regulates expression of genes involved in calcium metabolism [232, 233].

CROCC (alias: rootletin) is involved in centrosome cohesion during mitosis [234]. This activity is aligned to calibration by catenin-β1 of centrosomes [33, 235].

LIN28A cooperates with Wnt signalling by suppressing let-7 RNA [236, 237].

PTPRU forms a complex with catenin-β1 and dephosphorylates and stabilizes the latter while enhancing adherence junctions [238, 239].

ILDR2 is located in the endoplasmic reticulum. Overexpression enhances endoplasmic reticulum stress [240] with concomitant autophagy [241] that in turn negatively regulates Wnt signalling [242, 243].

EXOC3 (alias: Sec6) is a component of the exocyst complex [244] that modulates E-cadherin and catenin-β1 availability by regulating the expression of α-catenin [245].

RUFY3 strengthens F-actin-enriched protrusive structures at the cell periphery [246].

SUV39H1 is a histone methyltransferase that binds to parafibromin and suppresses Wnt target genes such as cyclin D1 and c-myc [247]. SHP2-dependent dephosphorylation of parafibromin enhances its affinity to catenin-β1 to form a complex that induces expression of Wnt target genes.

NOTCH3 acts as an antagonist of the Wnt cascade [248].

SLC7A10 forms a high-affinity D-serine receptor [249] that modulates activity of NMDA glutamate receptor and hence calcium influx [250].

KDM4B is a histone de-methylase that forms a complex with TCF4 and catenin-β1 to meditate transcriptional activation of downstream genes [251].

ATP13A1 regulates Mn^{2+} transport into endoplasmic reticulum and hence availability of this ion in the cytosol [252]. Mn2 is critical for functionality of protein phosphatase-1 [253] that controls stability of catenin-β1 [254]. $Mn^{2+}$is essential for functionality of integrin-linked kinase that inactivates GSK3β [255].

ARID3A enhances stability of its partner p53 [256]. Activated p53 cascade increases degradation of catenin-β1 [25] by stabilizing association of the latter with axin and hence phosphorylation and degradation of catenin-β1 [26].

FHOD3is involved in remodelling the actin cytoskeleton during cell migration [257].

TNFRSF11A (alias: RANK) amplifies Wnt responsiveness [258]. Further, RANKL signalling increases cytosolic Ca^{2+} via extracellular influx [259-261].

ABR acts as GTPase-activating protein for RAC and CDC42 [262].

GAS7 regulates actin polymerisation by interacting with N-WASP [263].

ASIC2 is a proton-gate cation channel that increases cytosolic calcium level [264, 265].

MYLK3 is a myosin light chain kinase that activates receptor-activated non-selective calcium permeant cation channel (TRPC5) to increase cytosolic Ca^{2+} [266, 267].

VAC14 is essential for generation of signalling lipid phosphatidylinositol 3,5-bisphosphate [268, 269]. The latter activates plasma membrane and endo-lysosomal TRPLM1 channel that triggers voltage-independent calcium flux [270, 271].

FERMT2 (alias: kindlin-2) complexes with active β-catenin and TCF4 and promotes transcriptional activity of Wnt cascade [272].

PELI2 activates MAPK pathway [273] that in turn synergizes with Wnt cascade by Akt-dependent inactivation of GSK3β [274].

PPAP2B (alias:Lipid phosphate phosphatase 3) stabilizes catenin-β1 [275].

PTPRG belongs to protein tyrosine phosphatase family that bind to and modulate phosphorylation level of catenin-β1 [276].

KALRN interacts with Rac1 [277] that in turn controls nuclear shuttling of catenin-β1 [58].

IFT122: The C. elegans homologue is involved in regulation of CDC42[278] and also unfolded protein response and autophagy [279].

CLSTN2: CLSTN1 is involved in the transport of NMDA receptors [280].

SYNE-1: Nesprin family are involved in regulation of cytoskeleton/nucleoskeleton [281] and also regulate signalling by catenin-β1 [282].

IL9R: Signalling by IL9/IL9R activates JAK/STAT signalling cascade [283] that in turn positively regulates MAPK and PI3K cascades [150, 151].

LAS1L is required for ribosomal biogenesis [284]. Inhibition of ribosomal biogenesis elicits biogenesis stress response characterised by activation of p53 [284-286]. The antagonistic interaction of p53 and catenin-β1 was foreshadowed previously in the text.

RBBP7 is involved in regulation of centromeres by binding and stabilizing CENP-A [287].

WWC3: WWC family of proteins negatively regulate hippo cascade by antagonizing Yap [288]. Yap/Taz are essential for inactivation of β-catenin through recruitment of β-TrCP [289].

UBE2M activates NEDD8 that in turn triggers β-TrCP-dependent ubiquitination and degradation of catenin-β1 [290, 291].

SHC3 binds to trk family of receptor tyrosine kinases and enhances the activation of MAPK downstream of trk kinases [292].

FCHO1 acts as a nucleator for clathrin-mediated endocytosis [293] that is central to Wnt-mediated stabilization of catenin-β1 [294].

SIN3B: Sin3 family of transcriptional repressors oppose the activity of wnt signalling by suppressing the transcription of protein phosphatase-1β [295].

OLFM2 interacts with GluR2 subunit of AMPAR [296]. Presence of GluR2 subunit makes AMPAR impermeable to calcium [297].

HGS regulates recycling and degradation of membrane receptors. HGS alters trafficking of E-cadherin from membrane to lysosomal degradation[298] and hence increases the cytoplasmic pool of catenin-β1 [299].

MAP3K14 (alias: NIK) activates NF-κβ [300]. NF-κβ in turn physically binds to and decreases the level of free catenin-β1 [301].

YWHAE: Post-phosphorylation by Akt, this protein (alias: 14-3-3ε) antagonizes Wnt signalling by sequestering catenin-β1 in the cytoplasm in a complex composed of 14-3-3ε, catenin-β1 and chibby [302].

CENPN is critical for centromere assembly [303].

NLRC5 synergizes with Wnt/catenin signalling cascade[304]. Inhibition of NF-κβ by NLRC5 [305] may partially explain this observation.

NOD2 activates NF-κβ [306] that in turn physically binds to and decreases the level of free catenin-β1 [301].

MOK (alias: RAGE) stabilizes catenin-β1 in the cytosol [307].

RPS6KA5 (alias: MSK1) phosphorylates and promoted nuclear translocation of catenin-β1 [308].

AKAP6 co-localizes with and regulates the activity of sarcoplasmic reticulum Ca^{2+} release channel or ryanodine receptor [309].

UGGT2 regulates unfolded protein response in the endoplasmic reticulum [310]. Unfolded protein response leads to autophagy [311] with concomitant degradation of catenin-β1.

NCAPD3 is critical for chromosome condensation during mitosis [312].

OPCML acts as a membrane-bound suppressor of EPHA2, FGFR1, FGFR3, ERBB2 [313]. These receptor tyrosine kinases stabilize catenin-β1 as documented previously.

MACROD1 (alias: LRP16) partners with and increases transcriptional activity of estrogen receptor alpha [314] and androgen receptor[315]. While the estrogen receptor synergizes with catenin-β1 [316], interplay between androgen receptor and catenin-β1 is antagonistic [317].

PRR5L, a structural component of mTORC2 complex [318] that regulates autophagy and availability of catenin-β1.

CARS: Glutathione is a reducing agent for reactive oxygen species (ROS)-detoxifying enzymes.Glutathione is synthesized from cysteine as follows [319]:

1. L-glutamate + L-cysteine + ATP → γ-glutamyl-L-cysteine + ADP + Pi
2. γ-glutamyl-L-cysteine + L-glycine + ATP → GSH + ADP + Pi

Inhibition of CARS (cysteinyl-TRNA synthetase) leads to elevation of intracellular cysteine level that is used as substrate in the first rate-limiting step of glutathione synthesis [320]. Availability of glutathione regulates redox status of cell. Redox status in turn controls thioredoxin activity. Thioredoxin maintains an inactive pool of Dvl and upon redox-dependent dissociation of thioredoxin and Dvl, Wnt signalling can stabilize catenin-β1 [145, 146].

TNNT3 regulates calcium channel Cavβ1a [321].

References

1. Grumolato L, Liu G, Mong P, Mudbhary R, Biswas R, Arroyave R, Vijayakumar S, Economides AN, Aaronson SA: **Canonical and noncanonical Wnts use a common mechanism to activate completely unrelated coreceptors.** *Genes Dev* 2010, **24:**2517-2530.
2. Steinfeld R, Van Den Berghe H, David G: **Stimulation of fibroblast growth factor receptor-1 occupancy and signaling by cell surface-associated syndecans and glypican.** *J Cell Biol* 1996, **133:**405-416.

3. Lin X, Zhang Y, Liu L, McKeehan WL, Shen Y, Song S, Wang F: **FRS2alpha is essential for the fibroblast growth factor to regulate the mTOR pathway and autophagy in mouse embryonic fibroblasts.** *Int J Biol Sci* 2011, **7:**1114-1121.
4. Kuhn K, Cott C, Bohler S, Aigal S, Zheng S, Villringer S, Imberty A, Claudinon J, Romer W: **The interplay of autophagy and beta-Catenin signaling regulates differentiation in acute myeloid leukemia.** *Cell Death Discov* 2015, **1:**15031.
5. Viard P, Butcher AJ, Halet G, Davies A, Nurnberg B, Heblich F, Dolphin AC: **PI3K promotes voltage-dependent calcium channel trafficking to the plasma membrane.** *Nat Neurosci* 2004, **7:**939-946.
6. Berridge MJ: **Inositol trisphosphate and calcium signalling mechanisms.** *Biochim Biophys Acta* 2009, **1793:**933-940.
7. Gopal S, Sogaard P, Multhaupt HA, Pataki C, Okina E, Xian X, Pedersen ME, Stevens T, Griesbeck O, Park PW, et al: **Transmembrane proteoglycans control stretch-activated channels to set cytosolic calcium levels.** *J Cell Biol* 2015, **210:**1199-1211.
8. Li Q, Stuenkel EL: **Calcium negatively modulates calmodulin interaction with IQGAP1.** *Biochem Biophys Res Commun* 2004, **317:**787-795.
9. Kholmanskikh SS, Koeller HB, Wynshaw-Boris A, Gomez T, Letourneau PC, Ross ME: **Calcium-dependent interaction of Lis1 with IQGAP1 and Cdc42 promotes neuronal motility.** *Nat Neurosci* 2006, **9:**50-57.
10. Fukata M, Kuroda S, Nakagawa M, Kawajiri A, Itoh N, Shoji I, Matsuura Y, Yonehara S, Fujisawa H, Kikuchi A, Kaibuchi K: **Cdc42 and Rac1 regulate the interaction of IQGAP1 with beta-catenin.** *J Biol Chem* 1999, **274:**26044-26050.
11. Goto T, Sato A, Adachi S, Iemura S, Natsume T, Shibuya H: **IQGAP1 protein regulates nuclear localization of beta-catenin via importin-beta5 protein in Wnt signaling.** *J Biol Chem* 2013, **288:**36351-36360.
12. Song B, Lai B, Zheng Z, Zhang Y, Luo J, Wang C, Chen Y, Woodgett JR, Li M: **Inhibitory phosphorylation of GSK-3 by CaMKII couples depolarization to neuronal survival.** *J Biol Chem* 2010, **285:**41122-41134.
13. Zimmermann P, Tomatis D, Rosas M, Grootjans J, Leenaerts I, Degeest G, Reekmans G, Coomans C, David G: **Characterization of syntenin, a syndecan-binding PDZ protein, as a component of cell adhesion sites and microfilaments.** *Mol Biol Cell* 2001, **12:**339-350.
14. Dejima K, Kang S, Mitani S, Cosman PC, Chisholm AD: **Syndecan defines precise spindle orientation by modulating Wnt signaling in C. elegans.** *Development* 2014, **141:**4354-4365.
15. Loomis PA, Zheng L, Sekerkova G, Changyaleket B, Mugnaini E, Bartles JR: **Espin cross-links cause the elongation of microvillus-type parallel actin bundles in vivo.** *J Cell Biol* 2003, **163:**1045-1055.
16. Sekerkova G, Richter CP, Bartles JR: **Roles of the espin actin-bundling proteins in the morphogenesis and stabilization of hair cell stereocilia revealed in CBA/CaJ congenic jerker mice.** *PLoS Genet* 2011, **7:**e1002032.
17. Ivanov AI: **Actin motors that drive formation and disassembly of epithelial apical junctions.** *Front Biosci* 2008, **13:**6662-6681.
18. Schlessinger K, Hall A, Tolwinski N: **Wnt signaling pathways meet Rho GTPases.** *Genes Dev* 2009, **23:**265-277.
19. Biswas KH, Hartman KL, Yu CH, Harrison OJ, Song H, Smith AW, Huang WY, Lin WC, Guo Z, Padmanabhan A, et al: **E-cadherin junction formation involves an active kinetic nucleation process.** *Proc Natl Acad Sci U S A* 2015, **112:**10932-10937.
20. Stolting G, de Oliveira RC, Guzman RE, Miranda-Laferte E, Conrad R, Jordan N, Schmidt S, Hendriks J, Gensch T, Hidalgo P: **Direct interaction of CaVbeta with actin up-regulates L-type calcium currents in HL-1 cardiomyocytes.** *J Biol Chem* 2015, **290:**4561-4572.
21. Cho K, Lee M, Gu D, Munoz WA, Ji H, Kloc M, McCrea PD: **Kazrin, and its binding partners ARVCF- and delta-catenin, are required for Xenopus laevis craniofacial development.** *Dev Dyn* 2011, **240:**2601-2612.
22. Cho K, Vaught TG, Ji H, Gu D, Papasakelariou-Yared C, Horstmann N, Jennings JM, Lee M, Sevilla LM, Kloc M, et al: **Xenopus Kazrin interacts with ARVCF-catenin, spectrin and**

p190B RhoGAP, and modulates RhoA activity and epithelial integrity. *J Cell Sci* 2010, **123:**4128-4144.
23. Fang X, Ji H, Kim SW, Park JI, Vaught TG, Anastasiadis PZ, Ciesiolka M, McCrea PD: **Vertebrate development requires ARVCF and p120 catenins and their interplay with RhoA and Rac.** *J Cell Biol* 2004, **165:**87-98.
24. Bagchi A, Papazoglu C, Wu Y, Capurso D, Brodt M, Francis D, Bredel M, Vogel H, Mills AA: **CHD5 is a tumor suppressor at human 1p36.** *Cell* 2007, **128:**459-475.
25. Sadot E, Geiger B, Oren M, Ben-Ze'ev A: **Down-regulation of beta-catenin by activated p53.** *Mol Cell Biol* 2001, **21:**6768-6781.
26. Levina E, Oren M, Ben-Ze'ev A: **Downregulation of beta-catenin by p53 involves changes in the rate of beta-catenin phosphorylation and Axin dynamics.** *Oncogene* 2004, **23:**4444-4453.
27. Scott LM, Mueller L, Collins SJ: **E3, a hematopoietic-specific transcript directly regulated by the retinoic acid receptor alpha.** *Blood* 1996, **88:**2517-2530.
28. Pak Y, Glowacka WK, Bruce MC, Pham N, Rotin D: **Transport of LAPTM5 to lysosomes requires association with the ubiquitin ligase Nedd4, but not LAPTM5 ubiquitination.** *J Cell Biol* 2006, **175:**631-645.
29. Nethe M, de Kreuk BJ, Tauriello DV, Anthony EC, Snoek B, Stumpel T, Salinas PC, Maurice MM, Geerts D, Deelder AM, et al: **Rac1 acts in conjunction with Nedd4 and dishevelled-1 to promote maturation of cell-cell contacts.** *J Cell Sci* 2012, **125:**3430-3442.
30. Bilic J, Huang YL, Davidson G, Zimmermann T, Cruciat CM, Bienz M, Niehrs C: **Wnt induces LRP6 signalosomes and promotes dishevelled-dependent LRP6 phosphorylation.** *Science* 2007, **316:**1619-1622.
31. Wei W, Li M, Wang J, Nie F, Li L: **The E3 ubiquitin ligase ITCH negatively regulates canonical Wnt signaling by targeting dishevelled protein.** *Mol Cell Biol* 2012, **32:**3903-3912.
32. Chen C, Tian F, Lu L, Wang Y, Xiao Z, Yu C, Yu X: **Characterization of Cep85 - a new antagonist of Nek2A that is involved in the regulation of centrosome disjunction.** *J Cell Sci* 2015, **128:**3290-3303.
33. Mbom BC, Siemers KA, Ostrowski MA, Nelson WJ, Barth AI: **Nek2 phosphorylates and stabilizes beta-catenin at mitotic centrosomes downstream of Plk1.** *Mol Biol Cell* 2014, **25:**977-991.
34. Schlesinger A, Shelton CA, Maloof JN, Meneghini M, Bowerman B: **Wnt pathway components orient a mitotic spindle in the early Caenorhabditis elegans embryo without requiring gene transcription in the responding cell.** *Genes Dev* 1999, **13:**2028-2038.
35. Young SZ, Platel JC, Nielsen JV, Jensen NA, Bordey A: **GABA(A) Increases Calcium in Subventricular Zone Astrocyte-Like Cells Through L- and T-Type Voltage-Gated Calcium Channels.** *Front Cell Neurosci* 2010, **4:**8.
36. Capdevila JH, Falck JR, Harris RC: **Cytochrome P450 and arachidonic acid bioactivation. Molecular and functional properties of the arachidonate monooxygenase.** *J Lipid Res* 2000, **41:**163-181.
37. Rzigalinski BA, Willoughby KA, Hoffman SW, Falck JR, Ellis EF: **Calcium influx factor, further evidence it is 5, 6-epoxyeicosatrienoic acid.** *J Biol Chem* 1999, **274:**175-182.
38. Putney JW, Jr., Bird GS: **The inositol phosphate-calcium signaling system in nonexcitable cells.** *Endocr Rev* 1993, **14:**610-631.
39. Takahata M, Inoue Y, Tsuda H, Imoto I, Koinuma D, Hayashi M, Ichikura T, Yamori T, Nagasaki K, Yoshida M, et al: **SKI and MEL1 cooperate to inhibit transforming growth factor-beta signal in gastric cancer cells.** *J Biol Chem* 2009, **284:**3334-3344.
40. Warner DR, Horn KH, Mudd L, Webb CL, Greene RM, Pisano MM: **PRDM16/MEL1: a novel Smad binding protein expressed in murine embryonic orofacial tissue.** *Biochim Biophys Acta* 2007, **1773:**814-820.
41. Jian H, Shen X, Liu I, Semenov M, He X, Wang XF: **Smad3-dependent nuclear translocation of beta-catenin is required for TGF-beta1-induced proliferation of bone marrow-derived adult human mesenchymal stem cells.** *Genes Dev* 2006, **20:**666-674.

42. Dachsel JC, Ngok SP, Lewis-Tuffin LJ, Kourtidis A, Geyer R, Johnston L, Feathers R, Anastasiadis PZ: **The Rho guanine nucleotide exchange factor Syx regulates the balance of dia and ROCK activities to promote polarized-cancer-cell migration.** *Mol Cell Biol* 2013, **33:**4909-4918.
43. Zaoui K, Benseddik K, Daou P, Salaun D, Badache A: **ErbB2 receptor controls microtubule capture by recruiting ACF7 to the plasma membrane of migrating cells.** *Proc Natl Acad Sci U S A* 2010, **107:**18517-18522.
44. Ashton RS, Conway A, Pangarkar C, Bergen J, Lim KI, Shah P, Bissell M, Schaffer DV: **Astrocytes regulate adult hippocampal neurogenesis through ephrin-B signaling.** *Nat Neurosci* 2012, **15:**1399-1406.
45. Habas R, Kato Y, He X: **Wnt/Frizzled activation of Rho regulates vertebrate gastrulation and requires a novel Formin homology protein Daam1.** *Cell* 2001, **107:**843-854.
46. Habas R, Dawid IB, He X: **Coactivation of Rac and Rho by Wnt/Frizzled signaling is required for vertebrate gastrulation.** *Genes Dev* 2003, **17:**295-309.
47. Takeuchi S, Katoh H, Negishi M: **Eph/ephrin reverse signalling induces axonal retraction through RhoA/ROCK pathway.** *J Biochem* 2015, **158:**245-252.
48. Tanaka M, Kamo T, Ota S, Sugimura H: **Association of Dishevelled with Eph tyrosine kinase receptor and ephrin mediates cell repulsion.** *EMBO J* 2003, **22:**847-858.
49. Eble JA, Kassner A, Niland S, Morgelin M, Grifka J, Grassel S: **Collagen XVI harbors an integrin alpha1 beta1 recognition site in its C-terminal domains.** *J Biol Chem* 2006, **281:**25745-25756.
50. Wu C, Dedhar S: **Integrin-linked kinase (ILK) and its interactors: a new paradigm for the coupling of extracellular matrix to actin cytoskeleton and signaling complexes.** *J Cell Biol* 2001, **155:**505-510.
51. Troussard AA, Mawji NM, Ong C, Mui A, St -Arnaud R, Dedhar S: **Conditional knock-out of integrin-linked kinase demonstrates an essential role in protein kinase B/Akt activation.** *J Biol Chem* 2003, **278:**22374-22378.
52. Wickstrom SA, Lange A, Hess MW, Polleux J, Spatz JP, Kruger M, Pfaller K, Lambacher A, Bloch W, Mann M, et al: **Integrin-linked kinase controls microtubule dynamics required for plasma membrane targeting of caveolae.** *Dev Cell* 2010, **19:**574-588.
53. Dedhar S, Jewell K, Rojiani M, Gray V: **The receptor for the basement membrane glycoprotein entactin is the integrin alpha 3/beta 1.** *J Biol Chem* 1992, **267:**18908-18914.
54. Gabriel LA, Wang LW, Bader H, Ho JC, Majors AK, Hollyfield JG, Traboulsi EI, Apte SS: **ADAMTSL4, a secreted glycoprotein widely distributed in the eye, binds fibrillin-1 microfibrils and accelerates microfibril biogenesis.** *Invest Ophthalmol Vis Sci* 2012, **53:**461-469.
55. Cicanic M, Sykova E, Vargova L: **Bral1: "Superglue" for the extracellular matrix in the brain white matter.** *Int J Biochem Cell Biol* 2012, **44:**596-599.
56. Chopra A, Murray ME, Byfield FJ, Mendez MG, Halleluyan R, Restle DJ, Raz-Ben Aroush D, Galie PA, Pogoda K, Bucki R, et al: **Augmentation of integrin-mediated mechanotransduction by hyaluronic acid.** *Biomaterials* 2014, **35:**71-82.
57. Feng M, Bao Y, Li Z, Li J, Gong M, Lam S, Wang J, Marzese DM, Donovan N, Tan EY, et al: **RASAL2 activates RAC1 to promote triple-negative breast cancer progression.** *J Clin Invest* 2014, **124:**5291-5304.
58. Wu X, Tu X, Joeng KS, Hilton MJ, Williams DA, Long F: **Rac1 activation controls nuclear localization of beta-catenin during canonical Wnt signaling.** *Cell* 2008, **133:**340-353.
59. Ray RM, Vaidya RJ, Johnson LR: **MEK/ERK regulates adherens junctions and migration through Rac1.** *Cell Motil Cytoskeleton* 2007, **64:**143-156.
60. Koronakis V, Hume PJ, Humphreys D, Liu T, Horning O, Jensen ON, McGhie EJ: **WAVE regulatory complex activation by cooperating GTPases Arf and Rac1.** *Proc Natl Acad Sci U S A* 2011, **108:**14449-14454.
61. Guo F, Debidda M, Yang L, Williams DA, Zheng Y: **Genetic deletion of Rac1 GTPase reveals its critical role in actin stress fiber formation and focal adhesion complex assembly.** *J Biol Chem* 2006, **281:**18652-18659.

62. Morris EJ, Ji JY, Yang F, Di Stefano L, Herr A, Moon NS, Kwon EJ, Haigis KM, Naar AM, Dyson NJ: **E2F1 represses beta-catenin transcription and is antagonized by both pRB and CDK8.** *Nature* 2008, **455:**552-556.
63. Wu Z, Zheng S, Li Z, Tan J, Yu Q: **E2F1 suppresses Wnt/beta-catenin activity through transactivation of beta-catenin interacting protein ICAT.** *Oncogene* 2011, **30:**3979-3984.
64. Kormish JD, Sinner D, Zorn AM: **Interactions between SOX factors and Wnt/beta-catenin signaling in development and disease.** *Dev Dyn* 2010, **239:**56-68.
65. Melichar HJ, Narayan K, Der SD, Hiraoka Y, Gardiol N, Jeannet G, Held W, Chambers CA, Kang J: **Regulation of gammadelta versus alphabeta T lymphocyte differentiation by the transcription factor SOX13.** *Science* 2007, **315:**230-233.
66. Yabuta Y, Ohta H, Abe T, Kurimoto K, Chuma S, Saitou M: **TDRD5 is required for retrotransposon silencing, chromatoid body assembly, and spermiogenesis in mice.** *J Cell Biol* 2011, **192:**781-795.
67. Kuwabara T, Hsieh J, Muotri A, Yeo G, Warashina M, Lie DC, Moore L, Nakashima K, Asashima M, Gage FH: **Wnt-mediated activation of NeuroD1 and retro-elements during adult neurogenesis.** *Nat Neurosci* 2009, **12:**1097-1105.
68. Meyer RD, Sacks DB, Rahimi N: **IQGAP1-dependent signaling pathway regulates endothelial cell proliferation and angiogenesis.** *PLoS One* 2008, **3:**e3848.
69. Sun Z, Li X, Massena S, Kutschera S, Padhan N, Gualandi L, Sundvold-Gjerstad V, Gustafsson K, Choy WW, Zang G, et al: **VEGFR2 induces c-Src signaling and vascular permeability in vivo via the adaptor protein TSAd.** *J Exp Med* 2012, **209:**1363-1377.
70. Grohmanova K, Schlaepfer D, Hess D, Gutierrez P, Beck M, Kroschewski R: **Phosphorylation of IQGAP1 modulates its binding to Cdc42, revealing a new type of rho-GTPase regulator.** *J Biol Chem* 2004, **279:**48495-48504.
71. Shindo M, Wada H, Kaido M, Tateno M, Aigaki T, Tsuda L, Hayashi S: **Dual function of Src in the maintenance of adherens junctions during tracheal epithelial morphogenesis.** *Development* 2008, **135:**1355-1364.
72. Karni R, Gus Y, Dor Y, Meyuhas O, Levitzki A: **Active Src elevates the expression of beta-catenin by enhancement of cap-dependent translation.** *Mol Cell Biol* 2005, **25:**5031-5039.
73. Carter M, Chen X, Slowinska B, Minnerath S, Glickstein S, Shi L, Campagne F, Weinstein H, Ross ME: **Crooked tail (Cd) model of human folate-responsive neural tube defects is mutated in Wnt coreceptor lipoprotein receptor-related protein 6.** *Proc Natl Acad Sci U S A* 2005, **102:**12843-12848.
74. Gray JD, Nakouzi G, Slowinska-Castaldo B, Dazard JE, Rao JS, Nadeau JH, Ross ME: **Functional interactions between the LRP6 WNT co-receptor and folate supplementation.** *Hum Mol Genet* 2010, **19:**4560-4572.
75. Lipton SA, Kim WK, Choi YB, Kumar S, D'Emilia DM, Rayudu PV, Arnelle DR, Stamler JS: **Neurotoxicity associated with dual actions of homocysteine at the N-methyl-D-aspartate receptor.** *Proc Natl Acad Sci U S A* 1997, **94:**5923-5928.
76. Alves CH, Pellissier LP, Wijnholds J: **The CRB1 and adherens junction complex proteins in retinal development and maintenance.** *Prog Retin Eye Res* 2014, **40:**35-52.
77. van de Pavert SA, Kantardzhieva A, Malysheva A, Meuleman J, Versteeg I, Levelt C, Klooster J, Geiger S, Seeliger MW, Rashbass P, et al: **Crumbs homologue 1 is required for maintenance of photoreceptor cell polarization and adhesion during light exposure.** *J Cell Sci* 2004, **117:**4169-4177.
78. Harris KP, Tepass U: **Cdc42 and Par proteins stabilize dynamic adherens junctions in the Drosophila neuroectoderm through regulation of apical endocytosis.** *J Cell Biol* 2008, **183:**1129-1143.
79. Balsinde J, Winstead MV, Dennis EA: **Phospholipase A(2) regulation of arachidonic acid mobilization.** *FEBS Lett* 2002, **531:**2-6.
80. Wen ZH, Su YC, Lai PL, Zhang Y, Xu YF, Zhao A, Yao GY, Jia CH, Lin J, Xu S, et al: **Critical role of arachidonic acid-activated mTOR signaling in breast carcinogenesis and angiogenesis.** *Oncogene* 2013, **32:**160-170.
81. Roberts LA, Glenn H, Hahn CS, Jacobson BS: **Cdc42 and RhoA are differentially regulated during arachidonate-mediated HeLa cell adhesion.** *J Cell Physiol* 2003, **196:**196-205.

82. Mignen O, Thompson JL, Shuttleworth TJ: **Both Orai1 and Orai3 are essential components of the arachidonate-regulated Ca2+-selective (ARC) channels.** *J Physiol* 2008, **586:**185-195.
83. Mottola A, Antoniotti S, Lovisolo D, Munaron L: **Regulation of noncapacitative calcium entry by arachidonic acid and nitric oxide in endothelial cells.** *FASEB J* 2005, **19:**2075-2077.
84. Shuttleworth TJ: **Arachidonic acid, ARC channels, and Orai proteins.** *Cell Calcium* 2009, **45:**602-610.
85. Lee JH, Kang Y, Khare V, Jin ZY, Kang MY, Yoon Y, Hyun JW, Chung MH, Cho SI, Jun JY, et al: **The p53-inducible gene 3 (PIG3) contributes to early cellular response to DNA damage.** *Oncogene* 2010, **29:**1431-1450.
86. Nagaoka T, Karasawa H, Turbyville T, Rangel MC, Castro NP, Gonzales M, Baker A, Seno M, Lockett S, Greer YE, et al: **Cripto-1 enhances the canonical Wnt/beta-catenin signaling pathway by binding to LRP5 and LRP6 co-receptors.** *Cell Signal* 2013, **25:**178-189.
87. Kohno T, Matsuyuki H, Inagaki Y, Igarashi Y: **Sphingosine 1-phosphate promotes cell migration through the activation of Cdc42 in Edg-6/S1P4-expressing cells.** *Genes Cells* 2003, **8:**685-697.
88. Mattie M, Brooker G, Spiegel S: **Sphingosine-1-phosphate, a putative second messenger, mobilizes calcium from internal stores via an inositol trisphosphate-independent pathway.** *J Biol Chem* 1994, **269:**3181-3188.
89. Meyer zu Heringdorf D, Lass H, Alemany R, Laser KT, Neumann E, Zhang C, Schmidt M, Rauen U, Jakobs KH, van Koppen CJ: **Sphingosine kinase-mediated Ca2+ signalling by G-protein-coupled receptors.** *EMBO J* 1998, **17:**2830-2837.
90. van Koppen CJ, Meyer zu Heringdorf D, Alemany R, Jakobs KH: **Sphingosine kinase-mediated calcium signaling by muscarinic acetylcholine receptors.** *Life Sci* 2001, **68:**2535-2540.
91. Dzeja C, Hagen V, Kaupp UB, Frings S: **Ca2+ permeation in cyclic nucleotide-gated channels.** *EMBO J* 1999, **18:**131-144.
92. Serysheva E, Berhane H, Grumolato L, Demir K, Balmer S, Bodak M, Boutros M, Aaronson S, Mlodzik M, Jenny A: **Wnk kinases are positive regulators of canonical Wnt/beta-catenin signalling.** *EMBO Rep* 2013, **14:**718-725.
93. Alessi DR, Zhang J, Khanna A, Hochdorfer T, Shang Y, Kahle KT: **The WNK-SPAK/OSR1 pathway: master regulator of cation-chloride cotransporters.** *Sci Signal* 2014, **7:**re3.
94. Langosch D, Becker CM, Betz H: **The inhibitory glycine receptor: a ligand-gated chloride channel of the central nervous system.** *Eur J Biochem* 1990, **194:**1-8.
95. Miyake S, Yamashita T, Taniguchi M, Tamatani M, Sato K, Tohyama M: **Identification and characterization of a novel mitochondrial tricarboxylate carrier.** *Biochem Biophys Res Commun* 2002, **295:**463-468.
96. Catalina-Rodriguez O, Kolukula VK, Tomita Y, Preet A, Palmieri F, Wellstein A, Byers S, Giaccia AJ, Glasgow E, Albanese C, Avantaggiati ML: **The mitochondrial citrate transporter, CIC, is essential for mitochondrial homeostasis.** *Oncotarget* 2012, **3:**1220-1235.
97. Gnoni GV, Priore P, Geelen MJ, Siculella L: **The mitochondrial citrate carrier: metabolic role and regulation of its activity and expression.** *IUBMB Life* 2009, **61:**987-994.
98. Sheng R, Kim H, Lee H, Xin Y, Chen Y, Tian W, Cui Y, Choi JC, Doh J, Han JK, Cho W: **Cholesterol selectively activates canonical Wnt signalling over non-canonical Wnt signalling.** *Nat Commun* 2014, **5:**4393.
99. Wang L, Shao YY, Ballock RT: **Thyroid hormone interacts with the Wnt/beta-catenin signaling pathway in the terminal differentiation of growth plate chondrocytes.** *J Bone Miner Res* 2007, **22:**1988-1995.
100. Choi JW, Choi HS: **The regulatory effects of thyroid hormone on the activity of 3-hydroxy-3-methylglutaryl coenzyme A reductase.** *Endocr Res* 2000, **26:**1-21.
101. Milev MP, Hasaj B, Saint-Dic D, Snounou S, Zhao Q, Sacher M: **TRAMM/TrappC12 plays a role in chromosome congression, kinetochore stability, and CENP-E recruitment.** *J Cell Biol* 2015, **209:**221-234.

102. Mbom BC, Nelson WJ, Barth A: **beta-catenin at the centrosome: discrete pools of beta-catenin communicate during mitosis and may co-ordinate centrosome functions and cell cycle progression.** *Bioessays* 2013, **35:**804-809.
103. Puxeddu E, Uhart M, Li CC, Ahmad F, Pacheco-Rodriguez G, Manganiello VC, Moss J, Vaughan M: **Interaction of phosphodiesterase 3A with brefeldin A-inhibited guanine nucleotide-exchange proteins BIG1 and BIG2 and effect on ARF1 activity.** *Proc Natl Acad Sci U S A* 2009, **106:**6158-6163.
104. Li CC, Le K, Kato J, Moss J, Vaughan M: **Enhancement of beta-catenin activity by BIG1 plus BIG2 via Arf activation and cAMP signals.** *Proc Natl Acad Sci U S A* 2016, **113:**5946-5951.
105. Bar-Peled L, Chantranupong L, Cherniack AD, Chen WW, Ottina KA, Grabiner BC, Spear ED, Carter SL, Meyerson M, Sabatini DM: **A Tumor suppressor complex with GAP activity for the Rag GTPases that signal amino acid sufficiency to mTORC1.** *Science* 2013, **340:**1100-1106.
106. Kim JS, Ro SH, Kim M, Park HW, Semple IA, Park H, Cho US, Wang W, Guan KL, Karin M, Lee JH: **Sestrin2 inhibits mTORC1 through modulation of GATOR complexes.** *Sci Rep* 2015, **5:**9502.
107. Rocha N, Kuijl C, van der Kant R, Janssen L, Houben D, Janssen H, Zwart W, Neefjes J: **Cholesterol sensor ORP1L contacts the ER protein VAP to control Rab7-RILP-p150 Glued and late endosome positioning.** *J Cell Biol* 2009, **185:**1209-1225.
108. Johansson M, Lehto M, Tanhuanpaa K, Cover TL, Olkkonen VM: **The oxysterol-binding protein homologue ORP1L interacts with Rab7 and alters functional properties of late endocytic compartments.** *Mol Biol Cell* 2005, **16:**5480-5492.
109. Bellett G, Carter JM, Keynton J, Goldspink D, James C, Moss DK, Mogensen MM: **Microtubule plus-end and minus-end capture at adherens junctions is involved in the assembly of apico-basal arrays in polarised epithelial cells.** *Cell Motil Cytoskeleton* 2009, **66:**893-908.
110. Nakamura T, Hayashi T, Nasu-Nishimura Y, Sakaue F, Morishita Y, Okabe T, Ohwada S, Matsuura K, Akiyama T: **PX-RICS mediates ER-to-Golgi transport of the N-cadherin/beta-catenin complex.** *Genes Dev* 2008, **22:**1244-1256.
111. Li L, Kim E, Yuan H, Inoki K, Goraksha-Hicks P, Schiesher RL, Neufeld TP, Guan KL: **Regulation of mTORC1 by the Rab and Arf GTPases.** *J Biol Chem* 2010, **285:**19705-19709.
112. Lang B, Zhang L, Jiang G, Hu L, Lan W, Zhao L, Hunter I, Pruski M, Song NN, Huang Y, et al: **Control of cortex development by ULK4, a rare risk gene for mental disorders including schizophrenia.** *Sci Rep* 2016, **6:**31126.
113. Liu M, Guan Z, Shen Q, Flinter F, Dominguez L, Ahn JW, Collier DA, O'Brien T, Shen S: **Ulk4 Regulates Neural Stem Cell Pool.** *Stem Cells* 2016, **34:**2318-2331.
114. Chan EY, Longatti A, McKnight NC, Tooze SA: **Kinase-inactivated ULK proteins inhibit autophagy via their conserved C-terminal domains using an Atg13-independent mechanism.** *Mol Cell Biol* 2009, **29:**157-171.
115. Goehring AS, Pedroja BS, Hinke SA, Langeberg LK, Scott JD: **MyRIP anchors protein kinase A to the exocyst complex.** *J Biol Chem* 2007, **282:**33155-33167.
116. Qin N, Olcese R, Stefani E, Birnbaumer L: **Modulation of human neuronal alpha 1E-type calcium channel by alpha 2 delta-subunit.** *Am J Physiol* 1998, **274:**C1324-1331.
117. Bikkavilli RK, Feigin ME, Malbon CC: **p38 mitogen-activated protein kinase regulates canonical Wnt-beta-catenin signaling by inactivation of GSK3beta.** *J Cell Sci* 2008, **121:**3598-3607.
118. Rakhilin SV, Olson PA, Nishi A, Starkova NN, Fienberg AA, Nairn AC, Surmeier DJ, Greengard P: **A network of control mediated by regulator of calcium/calmodulin-dependent signaling.** *Science* 2004, **306:**698-701.
119. Schreiber R, Faria D, Skryabin BV, Wanitchakool P, Rock JR, Kunzelmann K: **Anoctamins support calcium-dependent chloride secretion by facilitating calcium signaling in adult mouse intestine.** *Pflugers Arch* 2015, **467:**1203-1213.

120. Vocke K, Dauner K, Hahn A, Ulbrich A, Broecker J, Keller S, Frings S, Mohrlen F: **Calmodulin-dependent activation and inactivation of anoctamin calcium-gated chloride channels.** *J Gen Physiol* 2013, **142:**381-404.
121. Jeong J, VanHouten JN, Dann P, Kim W, Sullivan C, Yu H, Liotta L, Espina V, Stern DF, Friedman PA, Wysolmerski JJ: **PMCA2 regulates HER2 protein kinase localization and signaling and promotes HER2-mediated breast cancer.** *Proc Natl Acad Sci U S A* 2016, **113:**E282-290.
122. Strehler EE, Zacharias DA: **Role of alternative splicing in generating isoform diversity among plasma membrane calcium pumps.** *Physiol Rev* 2001, **81:**21-50.
123. Mimoto MS, Kwon S, Green YS, Goldman D, Christian JL: **GATA2 regulates Wnt signaling to promote primitive red blood cell fate.** *Dev Biol* 2015, **407:**1-11.
124. Li Z, Wang Y, Zhang M, Xu P, Huang H, Wu D, Meng A: **The Amotl2 gene inhibits Wnt/beta-catenin signaling and regulates embryonic development in zebrafish.** *J Biol Chem* 2012, **287:**13005-13015.
125. Rawal N, Corti O, Sacchetti P, Ardilla-Osorio H, Sehat B, Brice A, Arenas E: **Parkin protects dopaminergic neurons from excessive Wnt/beta-catenin signaling.** *Biochem Biophys Res Commun* 2009, **388:**473-478.
126. Geisbrecht ER, Montell DJ: **Myosin VI is required for E-cadherin-mediated border cell migration.** *Nat Cell Biol* 2002, **4:**616-620.
127. Tervaniemi MH, Siitonen HA, Soderhall C, Minhas G, Vuola J, Tiala I, Sormunen R, Samuelsson L, Suomela S, Kere J, Elomaa O: **Centrosomal localization of the psoriasis candidate gene product, CCHCR1, supports a role in cytoskeletal organization.** *PLoS One* 2012, **7:**e49920.
128. Stork B, Engelke M, Frey J, Horejsi V, Hamm-Baarke A, Schraven B, Kurosaki T, Wienands J: **Grb2 and the non-T cell activation linker NTAL constitute a Ca(2+)-regulating signal circuit in B lymphocytes.** *Immunity* 2004, **21:**681-691.
129. Crampton SP, Wu B, Park EJ, Kim JH, Solomon C, Waterman ML, Hughes CC: **Integration of the beta-catenin-dependent Wnt pathway with integrin signaling through the adaptor molecule Grb2.** *PLoS One* 2009, **4:**e7841.
130. Dinarina A, Ruiz EJ, O'Loghlen A, Mouron S, Perez L, Nebreda AR: **Negative regulation of cell-cycle progression by RINGO/Speedy E.** *Biochem J* 2008, **410:**535-542.
131. Park CS, Kim SI, Lee MS, Youn CY, Kim DJ, Jho EH, Song WK: **Modulation of beta-catenin phosphorylation/degradation by cyclin-dependent kinase 2.** *J Biol Chem* 2004, **279:**19592-19599.
132. Akhmanova A, Hoogenraad CC, Drabek K, Stepanova T, Dortland B, Verkerk T, Vermeulen W, Burgering BM, De Zeeuw CI, Grosveld F, Galjart N: **Clasps are CLIP-115 and -170 associating proteins involved in the regional regulation of microtubule dynamics in motile fibroblasts.** *Cell* 2001, **104:**923-935.
133. Hoogenraad CC, Akhmanova A, Grosveld F, De Zeeuw CI, Galjart N: **Functional analysis of CLIP-115 and its binding to microtubules.** *J Cell Sci* 2000, **113 (Pt 12):**2285-2297.
134. Hur EM, Saijilafu, Lee BD, Kim SJ, Xu WL, Zhou FQ: **GSK3 controls axon growth via CLASP-mediated regulation of growth cone microtubules.** *Genes Dev* 2011, **25:**1968-1981.
135. Satoh K, Yanai H, Senda T, Kohu K, Nakamura T, Okumura N, Matsumine A, Kobayashi S, Toyoshima K, Akiyama T: **DAP-1, a novel protein that interacts with the guanylate kinase-like domains of hDLG and PSD-95.** *Genes Cells* 1997, **2:**415-424.
136. Lin Y, Skeberdis VA, Francesconi A, Bennett MV, Zukin RS: **Postsynaptic density protein-95 regulates NMDA channel gating and surface expression.** *J Neurosci* 2004, **24:**10138-10148.
137. Park WJ, Lee SE, Kwon NS, Baek KJ, Kim DS, Yun HY: **Leucine-rich glioma inactivated 3 associates with syntaxin 1.** *Neurosci Lett* 2008, **444:**240-244.
138. Cibulsky SM, Fei H, Levitan IB: **Syntaxin-1A binds to and modulates the Slo calcium-activated potassium channel via an interaction that excludes syntaxin binding to calcium channels.** *J Neurophysiol* 2005, **93:**1393-1405.
139. Yokoyama T, Kanno Y, Yamazaki Y, Takahara T, Miyata S, Nakamura T: **Trib1 links the MEK1/ERK pathway in myeloid leukemogenesis.** *Blood* 2010, **116:**2768-2775.

140. Ding Q, Xia W, Liu JC, Yang JY, Lee DF, Xia J, Bartholomeusz G, Li Y, Pan Y, Li Z, et al: **Erk associates with and primes GSK-3beta for its inactivation resulting in upregulation of beta-catenin.** *Mol Cell* 2005, **19:**159-170.
141. Yun MS, Kim SE, Jeon SH, Lee JS, Choi KY: **Both ERK and Wnt/beta-catenin pathways are involved in Wnt3a-induced proliferation.** *J Cell Sci* 2005, **118:**313-322.
142. Tanaka H, Katoh H, Negishi M: **Pragmin, a novel effector of Rnd2 GTPase, stimulates RhoA activity.** *J Biol Chem* 2006, **281:**10355-10364.
143. Rodrigues P, Macaya I, Bazzocco S, Mazzolini R, Andretta E, Dopeso H, Mateo-Lozano S, Bilic J, Carton-Garcia F, Nieto R, et al: **RHOA inactivation enhances Wnt signalling and promotes colorectal cancer.** *Nat Commun* 2014, **5:**5458.
144. Rharass T, Lemcke H, Lantow M, Kuznetsov SA, Weiss DG, Panakova D: **Ca2+-mediated mitochondrial reactive oxygen species metabolism augments Wnt/beta-catenin pathway activation to facilitate cell differentiation.** *J Biol Chem* 2014, **289:**27937-27951.
145. Funato Y, Michiue T, Asashima M, Miki H: **The thioredoxin-related redox-regulating protein nucleoredoxin inhibits Wnt-beta-catenin signalling through dishevelled.** *Nat Cell Biol* 2006, **8:**501-508.
146. Funato Y, Terabayashi T, Sakamoto R, Okuzaki D, Ichise H, Nojima H, Yoshida N, Miki H: **Nucleoredoxin sustains Wnt/beta-catenin signaling by retaining a pool of inactive dishevelled protein.** *Curr Biol* 2010, **20:**1945-1952.
147. Levy S, Allerston CK, Liveanu V, Habib MR, Gileadi O, Schuster G: **Identification of LACTB2, a metallo-beta-lactamase protein, as a human mitochondrial endoribonuclease.** *Nucleic Acids Res* 2016, **44:**1813-1832.
148. Huard CC, Tremblay CS, Magron A, Levesque G, Carreau M: **The Fanconi anemia pathway has a dual function in Dickkopf-1 transcriptional repression.** *Proc Natl Acad Sci U S A* 2014, **111:**2152-2157.
149. Pang Q, Fagerlie S, Christianson TA, Keeble W, Faulkner G, Diaz J, Rathbun RK, Bagby GC: **The Fanconi anemia protein FANCC binds to and facilitates the activation of STAT1 by gamma interferon and hematopoietic growth factors.** *Mol Cell Biol* 2000, **20:**4724-4735.
150. Abell K, Watson CJ: **The Jak/Stat pathway: a novel way to regulate PI3K activity.** *Cell Cycle* 2005, **4:**897-900.
151. Rawlings JS, Rosler KM, Harrison DA: **The JAK/STAT signaling pathway.** *J Cell Sci* 2004, **117:**1281-1283.
152. Moy I, Todorovic V, Dubash AD, Coon JS, Parker JB, Buranapramest M, Huang CC, Zhao H, Green KJ, Bulun SE: **Estrogen-dependent sushi domain containing 3 regulates cytoskeleton organization and migration in breast cancer cells.** *Oncogene* 2015, **34:**323-333.
153. Gao C, Chen G, Kuan SF, Zhang DH, Schlaepfer DD, Hu J: **FAK/PYK2 promotes the Wnt/beta-catenin pathway and intestinal tumorigenesis by phosphorylating GSK3beta.** *Elife* 2015, **4**.
154. Park HW, Jung H, Choi KH, Baik JH, Rhim H: **Direct interaction and functional coupling between voltage-gated CaV1.3 Ca2+ channel and GABAB receptor subunit 2.** *FEBS Lett* 2010, **584:**3317-3322.
155. de Bruyn KM, de Rooij J, Wolthuis RM, Rehmann H, Wesenbeek J, Cool RH, Wittinghofer AH, Bos JL: **RalGEF2, a pleckstrin homology domain containing guanine nucleotide exchange factor for Ral.** *J Biol Chem* 2000, **275:**29761-29766.
156. Balasubramanian N, Meier JA, Scott DW, Norambuena A, White MA, Schwartz MA: **RalA-exocyst complex regulates integrin-dependent membrane raft exocytosis and growth signaling.** *Curr Biol* 2010, **20:**75-79.
157. Ozhan G, Sezgin E, Wehner D, Pfister AS, Kuhl SJ, Kagermeier-Schenk B, Kuhl M, Schwille P, Weidinger G: **Lypd6 enhances Wnt/beta-catenin signaling by promoting Lrp6 phosphorylation in raft plasma membrane domains.** *Dev Cell* 2013, **26:**331-345.
158. Sakane H, Yamamoto H, Kikuchi A: **LRP6 is internalized by Dkk1 to suppress its phosphorylation in the lipid raft and is recycled for reuse.** *J Cell Sci* 2010, **123:**360-368.
159. Liu GH, Guan T, Datta K, Coppinger J, Yates J, 3rd, Gerace L: **Regulation of myoblast differentiation by the nuclear envelope protein NET39.** *Mol Cell Biol* 2009, **29:**5800-5812.

160. Kaczmarek LK: **Slack, Slick and Sodium-Activated Potassium Channels.** *ISRN Neurosci* 2013, **2013**.
161. Nosaka Y, Arai A, Miyasaka N, Miura O: **CrkL mediates Ras-dependent activation of the Raf/ERK pathway through the guanine nucleotide exchange factor C3G in hematopoietic cells stimulated with erythropoietin or interleukin-3.** *J Biol Chem* 1999, **274:**30154-30162.
162. Uemura N, Griffin JD: **The adapter protein Crkl links Cbl to C3G after integrin ligation and enhances cell migration.** *J Biol Chem* 1999, **274:**37525-37532.
163. Charrasse S, Comunale F, De Rossi S, Echard A, Gauthier-Rouviere C: **Rab35 regulates cadherin-mediated adherens junction formation and myoblast fusion.** *Mol Biol Cell* 2013, **24:**234-245.
164. Golan T, Yaniv A, Bafico A, Liu G, Gazit A: **The human Frizzled 6 (HFz6) acts as a negative regulator of the canonical Wnt. beta-catenin signaling cascade.** *J Biol Chem* 2004, **279:**14879-14888.
165. Chang H, Cahill H, Smallwood PM, Wang Y, Nathans J: **Identification of Astrotactin2 as a Genetic Modifier That Regulates the Global Orientation of Mammalian Hair Follicles.** *PLoS Genet* 2015, **11:**e1005532.
166. Johansen LD, Naumanen T, Knudsen A, Westerlund N, Gromova I, Junttila M, Nielsen C, Bottzauw T, Tolkovsky A, Westermarck J, et al: **IKAP localizes to membrane ruffles with filamin A and regulates actin cytoskeleton organization and cell migration.** *J Cell Sci* 2008, **121:**854-864.
167. Kim HA, Koo BK, Cho JH, Kim YY, Seong J, Chang HJ, Oh YM, Stange DE, Park JG, Hwang D, Kong YY: **Notch1 counteracts WNT/beta-catenin signaling through chromatin modification in colorectal cancer.** *J Clin Invest* 2012, **122:**3248-3259.
168. Kwon C, Cheng P, King IN, Andersen P, Shenje L, Nigam V, Srivastava D: **Notch post-translationally regulates beta-catenin protein in stem and progenitor cells.** *Nat Cell Biol* 2011, **13:**1244-1251.
169. Tian H, Biehs B, Chiu C, Siebel CW, Wu Y, Costa M, de Sauvage FJ, Klein OD: **Opposing activities of Notch and Wnt signaling regulate intestinal stem cells and gut homeostasis.** *Cell Rep* 2015, **11:**33-42.
170. Tang H, Ji F, Sun J, Xie Y, Xu Y, Yue H: **RBEL1 is required for osteosarcoma cell proliferation via inhibiting retinoblastoma 1.** *Mol Med Rep* 2016, **13:**1275-1280.
171. Ruiz S, Segrelles C, Santos M, Lara MF, Paramio JM: **Functional link between retinoblastoma family of proteins and the Wnt signaling pathway in mouse epidermis.** *Dev Dyn* 2004, **230:**410-418.
172. Yao CK, Lin YQ, Ly CV, Ohyama T, Haueter CM, Moiseenkova-Bell VY, Wensel TG, Bellen HJ: **A synaptic vesicle-associated Ca2+ channel promotes endocytosis and couples exocytosis to endocytosis.** *Cell* 2009, **138:**947-960.
173. Gujral TS, van Veelen W, Richardson DS, Myers SM, Meens JA, Acton DS, Dunach M, Elliott BE, Hoppener JW, Mulligan LM: **A novel RET kinase-beta-catenin signaling pathway contributes to tumorigenesis in thyroid carcinoma.** *Cancer Res* 2008, **68:**1338-1346.
174. Takahashi M: **The GDNF/RET signaling pathway and human diseases.** *Cytokine Growth Factor Rev* 2001, **12:**361-373.
175. Rhee J, Buchan T, Zukerberg L, Lilien J, Balsamo J: **Cables links Robo-bound Abl kinase to N-cadherin-bound beta-catenin to mediate Slit-induced modulation of adhesion and transcription.** *Nat Cell Biol* 2007, **9:**883-892.
176. Giannini AL, Vivanco M, Kypta RM: **alpha-catenin inhibits beta-catenin signaling by preventing formation of a beta-catenin*T-cell factor*DNA complex.** *J Biol Chem* 2000, **275:**21883-21888.
177. Kim DI, Kang M, Kim S, Lee J, Park Y, Chang I, Suh BC: **Molecular Basis of the Membrane Interaction of the beta2e Subunit of Voltage-Gated Ca(2+) Channels.** *Biophys J* 2015, **109:**922-935.
178. Cross DA, Alessi DR, Cohen P, Andjelkovich M, Hemmings BA: **Inhibition of glycogen synthase kinase-3 by insulin mediated by protein kinase B.** *Nature* 1995, **378:**785-789.

179. Bryja V, Gradl D, Schambony A, Arenas E, Schulte G: **Beta-arrestin is a necessary component of Wnt/beta-catenin signaling in vitro and in vivo.** *Proc Natl Acad Sci U S A* 2007, **104:**6690-6695.
180. He X, Semenov M, Tamai K, Zeng X: **LDL receptor-related proteins 5 and 6 in Wnt/beta-catenin signaling: arrows point the way.** *Development* 2004, **131:**1663-1677.
181. Ahn VE, Chu ML, Choi HJ, Tran D, Abo A, Weis WI: **Structural basis of Wnt signaling inhibition by Dickkopf binding to LRP5/6.** *Dev Cell* 2011, **21:**862-873.
182. Bao J, Zheng JJ, Wu D: **The structural basis of DKK-mediated inhibition of Wnt/LRP signaling.** *Sci Signal* 2012, **5:**pe22.
183. Eswaran J, von Kries JP, Marsden B, Longman E, Debreczeni JE, Ugochukwu E, Turnbull A, Lee WH, Knapp S, Barr AJ: **Crystal structures and inhibitor identification for PTPN5, PTPRR and PTPN7: a family of human MAPK-specific protein tyrosine phosphatases.** *Biochem J* 2006, **395:**483-491.
184. Ikeda S, Kishida S, Yamamoto H, Murai H, Koyama S, Kikuchi A: **Axin, a negative regulator of the Wnt signaling pathway, forms a complex with GSK-3beta and beta-catenin and promotes GSK-3beta-dependent phosphorylation of beta-catenin.** *EMBO J* 1998, **17:**1371-1384.
185. Kishida S, Yamamoto H, Ikeda S, Kishida M, Sakamoto I, Koyama S, Kikuchi A: **Axin, a negative regulator of the wnt signaling pathway, directly interacts with adenomatous polyposis coli and regulates the stabilization of beta-catenin.** *J Biol Chem* 1998, **273:**10823-10826.
186. Hussain NK, Jenna S, Glogauer M, Quinn CC, Wasiak S, Guipponi M, Antonarakis SE, Kay BK, Stossel TP, Lamarche-Vane N, McPherson PS: **Endocytic protein intersectin-l regulates actin assembly via Cdc42 and N-WASP.** *Nat Cell Biol* 2001, **3:**927-932.
187. Lan Z, Wu H, Li W, Wu S, Lu L, Xu M, Dai W: **Transforming activity of receptor tyrosine kinase tyro3 is mediated, at least in part, by the PI3 kinase-signaling pathway.** *Blood* 2000, **95:**633-638.
188. Nopparat J, Zhang J, Lu JP, Chen YH, Zheng D, Neufer PD, Fan JM, Hong H, Boykin C, Lu Q: **delta-Catenin, a Wnt/beta-catenin modulator, reveals inducible mutagenesis promoting cancer cell survival adaptation and metabolic reprogramming.** *Oncogene* 2015, **34:**1542-1552.
189. Toyokawa G, Cho HS, Masuda K, Yamane Y, Yoshimatsu M, Hayami S, Takawa M, Iwai Y, Daigo Y, Tsuchiya E, et al: **Histone lysine methyltransferase Wolf-Hirschhorn syndrome candidate 1 is involved in human carcinogenesis through regulation of the Wnt pathway.** *Neoplasia* 2011, **13:**887-898.
190. Brooks SP, Coccia M, Tang HR, Kanuga N, Machesky LM, Bailly M, Cheetham ME, Hardcastle AJ: **The Nance-Horan syndrome protein encodes a functional WAVE homology domain (WHD) and is important for co-ordinating actin remodelling and maintaining cell morphology.** *Hum Mol Genet* 2010, **19:**2421-2432.
191. Sharma S, Ang SL, Shaw M, Mackey DA, Gecz J, McAvoy JW, Craig JE: **Nance-Horan syndrome protein, NHS, associates with epithelial cell junctions.** *Hum Mol Genet* 2006, **15:**1972-1983.
192. Gambino F, Pavlowsky A, Begle A, Dupont JL, Bahi N, Courjaret R, Gardette R, Hadjkacem H, Skala H, Poulain B, et al: **IL1-receptor accessory protein-like 1 (IL1RAPL1), a protein involved in cognitive functions, regulates N-type Ca2+-channel and neurite elongation.** *Proc Natl Acad Sci U S A* 2007, **104:**9063-9068.
193. Kuhl M, Sheldahl LC, Malbon CC, Moon RT: **Ca(2+)/calmodulin-dependent protein kinase II is stimulated by Wnt and Frizzled homologs and promotes ventral cell fates in Xenopus.** *J Biol Chem* 2000, **275:**12701-12711.
194. Lehtonen S, Ryan JJ, Kudlicka K, Iino N, Zhou H, Farquhar MG: **Cell junction-associated proteins IQGAP1, MAGI-2, CASK, spectrins, and alpha-actinin are components of the nephrin multiprotein complex.** *Proc Natl Acad Sci U S A* 2005, **102:**9814-9819.
195. Lu CS, Hodge JJ, Mehren J, Sun XX, Griffith LC: **Regulation of the Ca2+/CaM-responsive pool of CaMKII by scaffold-dependent autophosphorylation.** *Neuron* 2003, **40:**1185-1197.

196. Liu Y, Borchert GL, Surazynski A, Phang JM: **Proline oxidase, a p53-induced gene, targets COX-2/PGE2 signaling to induce apoptosis and inhibit tumor growth in colorectal cancers.** *Oncogene* 2008, **27:**6729-6737.
197. Leimeister C, Steidl C, Schumacher N, Erhard S, Gessler M: **Developmental expression and biochemical characterization of Emu family members.** *Dev Biol* 2002, **249:**204-218.
198. Davidson G, Mao B, del Barco Barrantes I, Niehrs C: **Kremen proteins interact with Dickkopf1 to regulate anteroposterior CNS patterning.** *Development* 2002, **129:**5587-5596.
199. Mao B, Wu W, Davidson G, Marhold J, Li M, Mechler BM, Delius H, Hoppe D, Stannek P, Walter C, et al: **Kremen proteins are Dickkopf receptors that regulate Wnt/beta-catenin signalling.** *Nature* 2002, **417:**664-667.
200. Duex JE, Mullins MR, Sorkin A: **Recruitment of Uev1B to Hrs-containing endosomes and its effect on endosomal trafficking.** *Exp Cell Res* 2010, **316:**2136-2151.
201. Pradhan-Sundd T, Verheyen EM: **The Myopic-Ubpy-Hrs nexus enables endosomal recycling of Frizzled.** *Mol Biol Cell* 2015, **26:**3329-3342.
202. Lee HK, Laug D, Zhu W, Patel JM, Ung K, Arenkiel BR, Fancy SP, Mohila C, Deneen B: **Apcdd1 stimulates oligodendrocyte differentiation after white matter injury.** *Glia* 2015, **63:**1840-1849.
203. Shimomura Y, Agalliu D, Vonica A, Luria V, Wajid M, Baumer A, Belli S, Petukhova L, Schinzel A, Brivanlou AH, et al: **APCDD1 is a novel Wnt inhibitor mutated in hereditary hypotrichosis simplex.** *Nature* 2010, **464:**1043-1047.
204. Martinez-Garay I, Gil-Sanz C, Franco SJ, Espinosa A, Molnar Z, Mueller U: **Cadherin 2/4 signaling via PTP1B and catenins is crucial for nucleokinesis during radial neuronal migration in the neocortex.** *Development* 2016, **143:**2121-2134.
205. Kim I, Pan W, Jones SA, Zhang Y, Zhuang X, Wu D: **Clathrin and AP2 are required for PtdIns(4,5)P2-mediated formation of LRP6 signalosomes.** *J Cell Biol* 2013, **200:**419-428.
206. Barro Soria R, Spitzner M, Schreiber R, Kunzelmann K: **Bestrophin-1 enables Ca2+-activated Cl- conductance in epithelia.** *J Biol Chem* 2009, **284:**29405-29412.
207. Yalcin A, Clem B, Makoni S, Clem A, Nelson K, Thornburg J, Siow D, Lane AN, Brock SE, Goswami U, et al: **Selective inhibition of choline kinase simultaneously attenuates MAPK and PI3K/AKT signaling.** *Oncogene* 2010, **29:**139-149.
208. Asperti C, Pettinato E, de Curtis I: **Liprin-alpha1 affects the distribution of low-affinity beta1 integrins and stabilizes their permanence at the cell surface.** *Exp Cell Res* 2010, **316:**915-926.
209. Asperti C, Astro V, Totaro A, Paris S, de Curtis I: **Liprin-alpha1 promotes cell spreading on the extracellular matrix by affecting the distribution of activated integrins.** *J Cell Sci* 2009, **122:**3225-3232.
210. Hoogenraad CC, Feliu-Mojer MI, Spangler SA, Milstein AD, Dunah AW, Hung AY, Sheng M: **Liprinalpha1 degradation by calcium/calmodulin-dependent protein kinase II regulates LAR receptor tyrosine phosphatase distribution and dendrite development.** *Dev Cell* 2007, **12:**587-602.
211. Fielding AB, Lim S, Montgomery K, Dobreva I, Dedhar S: **A critical role of integrin-linked kinase, ch-TOG and TACC3 in centrosome clustering in cancer cells.** *Oncogene* 2011, **30:**521-534.
212. James AW, Shen J, Zhang X, Asatrian G, Goyal R, Kwak JH, Jiang L, Bengs B, Culiat CT, Turner AS, et al: **NELL-1 in the treatment of osteoporotic bone loss.** *Nat Commun* 2015, **6:**7362.
213. Kim WT, Kim H, Katanaev VL, Joon Lee S, Ishitani T, Cha B, Han JK, Jho EH: **Dual functions of DP1 promote biphasic Wnt-on and Wnt-off states during anteroposterior neural patterning.** *EMBO J* 2012, **31:**3384-3397.
214. Lund EG, Xie C, Kotti T, Turley SD, Dietschy JM, Russell DW: **Knockout of the cholesterol 24-hydroxylase gene in mice reveals a brain-specific mechanism of cholesterol turnover.** *J Biol Chem* 2003, **278:**22980-22988.
215. Lu B, Su Y, Das S, Liu J, Xia J, Ren D: **The neuronal channel NALCN contributes resting sodium permeability and is required for normal respiratory rhythm.** *Cell* 2007, **129:**371-383.

216. Ren D: **Sodium leak channels in neuronal excitability and rhythmic behaviors.** *Neuron* 2011, **72:**899-911.
217. Perrier AL, Massoulie J, Krejci E: **PRiMA: the membrane anchor of acetylcholinesterase in the brain.** *Neuron* 2002, **33:**275-285.
218. Liu J, Xia J, Cho KH, Clapham DE, Ren D: **CatSperbeta, a novel transmembrane protein in the CatSper channel complex.** *J Biol Chem* 2007, **282:**18945-18952.
219. Bouteille N, Driouch K, Hage PE, Sin S, Formstecher E, Camonis J, Lidereau R, Lallemand F: **Inhibition of the Wnt/beta-catenin pathway by the WWOX tumor suppressor protein.** *Oncogene* 2009, **28:**2569-2580.
220. Stanevich V, Jiang L, Satyshur KA, Li Y, Jeffrey PD, Li Z, Menden P, Semmelhack MF, Xing Y: **The structural basis for tight control of PP2A methylation and function by LCMT-1.** *Mol Cell* 2011, **41:**331-342.
221. Li X, Yost HJ, Virshup DM, Seeling JM: **Protein phosphatase 2A and its B56 regulatory subunit inhibit Wnt signaling in Xenopus.** *EMBO J* 2001, **20:**4122-4131.
222. Lin CF, Chen CL, Chiang CW, Jan MS, Huang WC, Lin YS: **GSK-3beta acts downstream of PP2A and the PI 3-kinase-Akt pathway, and upstream of caspase-2 in ceramide-induced mitochondrial apoptosis.** *J Cell Sci* 2007, **120:**2935-2943.
223. Ratcliffe MJ, Itoh K, Sokol SY: **A positive role for the PP2A catalytic subunit in Wnt signal transduction.** *J Biol Chem* 2000, **275:**35680-35683.
224. Feoktistov I, Murray JJ, Biaggioni I: **Positive modulation of intracellular Ca2+ levels by adenosine A2b receptors, prostacyclin, and prostaglandin E1 via a cholera toxin-sensitive mechanism in human erythroleukemia cells.** *Mol Pharmacol* 1994, **45:**1160-1167.
225. Jimenez AI, Castro E, Mirabet M, Franco R, Delicado EG, Miras-Portugal MT: **Potentiation of ATP calcium responses by A2B receptor stimulation and other signals coupled to Gs proteins in type-1 cerebellar astrocytes.** *Glia* 1999, **26:**119-128.
226. Mirabet M, Mallol J, Lluis C, Franco R: **Calcium mobilization in Jurkat cells via A2b adenosine receptors.** *Br J Pharmacol* 1997, **122:**1075-1082.
227. Schulte G, Fredholm BB: **Signalling from adenosine receptors to mitogen-activated protein kinases.** *Cell Signal* 2003, **15:**813-827.
228. Mahmoudi S, Henriksson S, Corcoran M, Mendez-Vidal C, Wiman KG, Farnebo M: **Wrap53, a Natural p53 Antisense Transcript Required for p53 Induction upon DNA Damage.** *Mol Cell* 2016, **64:**1009.
229. Nijnik A, Clare S, Hale C, Chen J, Raisen C, Mottram L, Lucas M, Estabel J, Ryder E, Adissu H, et al: **The role of sphingosine-1-phosphate transporter Spns2 in immune system function.** *J Immunol* 2012, **189:**102-111.
230. Matsuzaki E, Hiratsuka S, Hamachi T, Takahashi-Yanaga F, Hashimoto Y, Higashi K, Kobayashi M, Hirofuji T, Hirata M, Maeda K: **Sphingosine-1-phosphate promotes the nuclear translocation of beta-catenin and thereby induces osteoprotegerin gene expression in osteoblast-like cell lines.** *Bone* 2013, **55:**315-324.
231. Pfisterer SG, Mauthe M, Codogno P, Proikas-Cezanne T: **Ca2+/calmodulin-dependent kinase (CaMK) signaling via CaMKI and AMP-activated protein kinase contributes to the regulation of WIPI-1 at the onset of autophagy.** *Mol Pharmacol* 2011, **80:**1066-1075.
232. Finkler A, Ashery-Padan R, Fromm H: **CAMTAs: calmodulin-binding transcription activators from plants to human.** *FEBS Lett* 2007, **581:**3893-3898.
233. Mollet IG, Malm HA, Wendt A, Orho-Melander M, Eliasson L: **Integrator of Stress Responses Calmodulin Binding Transcription Activator 1 (Camta1) Regulates miR-212/miR-132 Expression and Insulin Secretion.** *J Biol Chem* 2016, **291:**18440-18452.
234. Bahe S, Stierhof YD, Wilkinson CJ, Leiss F, Nigg EA: **Rootletin forms centriole-associated filaments and functions in centrosome cohesion.** *J Cell Biol* 2005, **171:**27-33.
235. Bahmanyar S, Kaplan DD, Deluca JG, Giddings TH, Jr., O'Toole ET, Winey M, Salmon ED, Casey PJ, Nelson WJ, Barth AI: **beta-Catenin is a Nek2 substrate involved in centrosome separation.** *Genes Dev* 2008, **22:**91-105.
236. Tu HC, Schwitalla S, Qian Z, LaPier GS, Yermalovich A, Ku YC, Chen SC, Viswanathan SR, Zhu H, Nishihara R, et al: **LIN28 cooperates with WNT signaling to drive invasive intestinal and colorectal adenocarcinoma in mice and humans.** *Genes Dev* 2015, **29:**1074-1086.

237. Cai WY, Wei TZ, Luo QC, Wu QW, Liu QF, Yang M, Ye GD, Wu JF, Chen YY, Sun GB, et al: **The Wnt-beta-catenin pathway represses let-7 microRNA expression through transactivation of Lin28 to augment breast cancer stem cell expansion.** *J Cell Sci* 2013, **126:**2877-2889.
238. Yan HX, He YQ, Dong H, Zhang P, Zeng JZ, Cao HF, Wu MC, Wang HY: **Physical and functional interaction between receptor-like protein tyrosine phosphatase PCP-2 and beta-catenin.** *Biochemistry* 2002, **41:**15854-15860.
239. Yan HX, Yang W, Zhang R, Chen L, Tang L, Zhai B, Liu SQ, Cao HF, Man XB, Wu HP, et al: **Protein-tyrosine phosphatase PCP-2 inhibits beta-catenin signaling and increases E-cadherin-dependent cell adhesion.** *J Biol Chem* 2006, **281:**15423-15433.
240. Watanabe K, Watson E, Cremona ML, Millings EJ, Lefkowitch JH, Fischer SG, LeDuc CA, Leibel RL: **ILDR2: an endoplasmic reticulum resident molecule mediating hepatic lipid homeostasis.** *PLoS One* 2013, **8:**e67234.
241. Yorimitsu T, Nair U, Yang Z, Klionsky DJ: **Endoplasmic reticulum stress triggers autophagy.** *J Biol Chem* 2006, **281:**30299-30304.
242. Gao C, Cao W, Bao L, Zuo W, Xie G, Cai T, Fu W, Zhang J, Wu W, Zhang X, Chen YG: **Autophagy negatively regulates Wnt signalling by promoting Dishevelled degradation.** *Nat Cell Biol* 2010, **12:**781-790.
243. Petherick KJ, Williams AC, Lane JD, Ordonez-Moran P, Huelsken J, Collard TJ, Smartt HJ, Batson J, Malik K, Paraskeva C, Greenhough A: **Autolysosomal beta-catenin degradation regulates Wnt-autophagy-p62 crosstalk.** *EMBO J* 2013, **32:**1903-1916.
244. Matern HT, Yeaman C, Nelson WJ, Scheller RH: **The Sec6/8 complex in mammalian cells: characterization of mammalian Sec3, subunit interactions, and expression of subunits in polarized cells.** *Proc Natl Acad Sci U S A* 2001, **98:**9648-9653.
245. Tanaka T, Iino M, Goto K: **Knockdown of Sec6 improves cell-cell adhesion by increasing alpha-E-catenin in oral cancer cells.** *FEBS Lett* 2012, **586:**924-933.
246. Wang G, Zhang Q, Song Y, Wang X, Guo Q, Zhang J, Li J, Han Y, Miao Z, Li F: **PAK1 regulates RUFY3-mediated gastric cancer cell migration and invasion.** *Cell Death Dis* 2015, **6:**e1682.
247. Takahashi A, Tsutsumi R, Kikuchi I, Obuse C, Saito Y, Seidi A, Karisch R, Fernandez M, Cho T, Ohnishi N, et al: **SHP2 tyrosine phosphatase converts parafibromin/Cdc73 from a tumor suppressor to an oncogenic driver.** *Mol Cell* 2011, **43:**45-56.
248. Zhang Q, Lu C, Fang T, Wang Y, Hu W, Qiao J, Liu B, Liu J, Chen N, Li M, Zhu R: **Notch3 functions as a regulator of cell self-renewal by interacting with the beta-catenin pathway in hepatocellular carcinoma.** *Oncotarget* 2015, **6:**3669-3679.
249. Nakauchi J, Matsuo H, Kim DK, Goto A, Chairoungdua A, Cha SH, Inatomi J, Shiokawa Y, Yamaguchi K, Saito I, et al: **Cloning and characterization of a human brain Na(+)-independent transporter for small neutral amino acids that transports D-serine with high affinity.** *Neurosci Lett* 2000, **287:**231-235.
250. Wolosker H: **D-serine regulation of NMDA receptor activity.** *Sci STKE* 2006, **2006:**pe41.
251. Berry WL, Kim TD, Janknecht R: **Stimulation of beta-catenin and colon cancer cell growth by the KDM4B histone demethylase.** *Int J Oncol* 2014, **44:**1341-1348.
252. Cohen Y, Megyeri M, Chen OC, Condomitti G, Riezman I, Loizides-Mangold U, Abdul-Sada A, Rimon N, Riezman H, Platt FM, et al: **The yeast p5 type ATPase, spf1, regulates manganese transport into the endoplasmic reticulum.** *PLoS One* 2013, **8:**e85519.
253. Nishito Y, Usui H, Tanabe O, Shimizu M, Takeda M: **Interconversion of Mn(2+)-dependent and -independent protein phosphatase 2A from human erythrocytes: role of Zn(2+) and Fe(2+) in protein phosphatase 2A.** *J Biochem* 1999, **126:**632-638.
254. Luo W, Peterson A, Garcia BA, Coombs G, Kofahl B, Heinrich R, Shabanowitz J, Hunt DF, Yost HJ, Virshup DM: **Protein phosphatase 1 regulates assembly and function of the beta-catenin degradation complex.** *EMBO J* 2007, **26:**1511-1521.
255. Maydan M, McDonald PC, Sanghera J, Yan J, Rallis C, Pinchin S, Hannigan GE, Foster LJ, Ish-Horowicz D, Walsh MP, Dedhar S: **Integrin-linked kinase is a functional Mn2+-dependent protein kinase that regulates glycogen synthase kinase-3beta (GSK-3beta) phosphorylation.** *PLoS One* 2010, **5:**e12356.

256. Lestari W, Ichwan SJ, Otsu M, Yamada S, Iseki S, Shimizu S, Ikeda MA: **Cooperation between ARID3A and p53 in the transcriptional activation of p21WAF1 in response to DNA damage.** *Biochem Biophys Res Commun* 2012, **417:**710-716.
257. Paul NR, Allen JL, Chapman A, Morlan-Mairal M, Zindy E, Jacquemet G, Fernandez del Ama L, Ferizovic N, Green DM, Howe JD, et al: **alpha5beta1 integrin recycling promotes Arp2/3-independent cancer cell invasion via the formin FHOD3.** *J Cell Biol* 2015, **210:**1013-1031.
258. Joshi PA, Waterhouse PD, Kannan N, Narala S, Fang H, Di Grappa MA, Jackson HW, Penninger JM, Eaves C, Khokha R: **RANK Signaling Amplifies WNT-Responsive Mammary Progenitors through R-SPONDIN1.** *Stem Cell Reports* 2015, **5:**31-44.
259. Chamoux E, Bisson M, Payet MD, Roux S: **TRPV-5 mediates a receptor activator of NF-kappaB (RANK) ligand-induced increase in cytosolic Ca2+ in human osteoclasts and down-regulates bone resorption.** *J Biol Chem* 2010, **285:**25354-25362.
260. Komarova SV, Pilkington MF, Weidema AF, Dixon SJ, Sims SM: **RANK ligand-induced elevation of cytosolic Ca2+ accelerates nuclear translocation of nuclear factor kappa B in osteoclasts.** *J Biol Chem* 2003, **278:**8286-8293.
261. Takayanagi H, Kim S, Koga T, Nishina H, Isshiki M, Yoshida H, Saiura A, Isobe M, Yokochi T, Inoue J, et al: **Induction and activation of the transcription factor NFATc1 (NFAT2) integrate RANKL signaling in terminal differentiation of osteoclasts.** *Dev Cell* 2002, **3:**889-901.
262. Chuang TH, Xu X, Kaartinen V, Heisterkamp N, Groffen J, Bokoch GM: **Abr and Bcr are multifunctional regulators of the Rho GTP-binding protein family.** *Proc Natl Acad Sci U S A* 1995, **92:**10282-10286.
263. You JJ, Lin-Chao S: **Gas7 functions with N-WASP to regulate the neurite outgrowth of hippocampal neurons.** *J Biol Chem* 2010, **285:**11652-11666.
264. Liu XY, Zhang SZ, Ma XY, Wang H, Wu BH, Sun HL, Li X, Wei XL, Zheng JQ: **Knockdown of ASIC2a subunit aggravates injury of rat C6 glioma cells in acidosis.** *J Physiol Biochem* 2011, **67:**275-281.
265. Xiong ZG, Chu XP, Simon RP: **Ca2+ -permeable acid-sensing ion channels and ischemic brain injury.** *J Membr Biol* 2006, **209:**59-68.
266. Kim MT, Kim BJ, Lee JH, Kwon SC, Yeon DS, Yang DK, So I, Kim KW: **Involvement of calmodulin and myosin light chain kinase in activation of mTRPC5 expressed in HEK cells.** *Am J Physiol Cell Physiol* 2006, **290:**C1031-1040.
267. Shimizu S, Yoshida T, Wakamori M, Ishii M, Okada T, Takahashi M, Seto M, Sakurada K, Kiuchi Y, Mori Y: **Ca2+-calmodulin-dependent myosin light chain kinase is essential for activation of TRPC5 channels expressed in HEK293 cells.** *J Physiol* 2006, **570:**219-235.
268. Zhang Y, Zolov SN, Chow CY, Slutsky SG, Richardson SC, Piper RC, Yang B, Nau JJ, Westrick RJ, Morrison SJ, et al: **Loss of Vac14, a regulator of the signaling lipid phosphatidylinositol 3,5-bisphosphate, results in neurodegeneration in mice.** *Proc Natl Acad Sci U S A* 2007, **104:**17518-17523.
269. Zolov SN, Bridges D, Zhang Y, Lee WW, Riehle E, Verma R, Lenk GM, Converso-Baran K, Weide T, Albin RL, et al: **In vivo, Pikfyve generates PI(3,5)P2, which serves as both a signaling lipid and the major precursor for PI5P.** *Proc Natl Acad Sci U S A* 2012, **109:**17472-17477.
270. Dong XP, Shen D, Wang X, Dawson T, Li X, Zhang Q, Cheng X, Zhang Y, Weisman LS, Delling M, Xu H: **PI(3,5)P(2) controls membrane trafficking by direct activation of mucolipin Ca(2+) release channels in the endolysosome.** *Nat Commun* 2010, **1:**38.
271. Feng X, Huang Y, Lu Y, Xiong J, Wong CO, Yang P, Xia J, Chen D, Du G, Venkatachalam K, et al: **Drosophila TRPML forms PI(3,5)P2-activated cation channels in both endolysosomes and plasma membrane.** *J Biol Chem* 2014, **289:**4262-4272.
272. Yu Y, Wu J, Wang Y, Zhao T, Ma B, Liu Y, Fang W, Zhu WG, Zhang H: **Kindlin 2 forms a transcriptional complex with beta-catenin and TCF4 to enhance Wnt signalling.** *EMBO Rep* 2012, **13:**750-758.
273. Jensen LE, Whitehead AS: **Pellino2 activates the mitogen activated protein kinase pathway.** *FEBS Lett* 2003, **545:**199-202.

274. Bikkavilli RK, Malbon CC: **Mitogen-activated protein kinases and Wnt/beta-catenin signaling: Molecular conversations among signaling pathways.** *Commun Integr Biol* 2009, **2:**46-49.
275. Humtsoe JO, Liu M, Malik AB, Wary KK: **Lipid phosphate phosphatase 3 stabilization of beta-catenin induces endothelial cell migration and formation of branching point structures.** *Mol Cell Biol* 2010, **30:**1593-1606.
276. Meng K, Rodriguez-Pena A, Dimitrov T, Chen W, Yamin M, Noda M, Deuel TF: **Pleiotrophin signals increased tyrosine phosphorylation of beta beta-catenin through inactivation of the intrinsic catalytic activity of the receptor-type protein tyrosine phosphatase beta/zeta.** *Proc Natl Acad Sci U S A* 2000, **97:**2603-2608.
277. Alam MR, Johnson RC, Darlington DN, Hand TA, Mains RE, Eipper BA: **Kalirin, a cytosolic protein with spectrin-like and GDP/GTP exchange factor-like domains that interacts with peptidylglycine alpha-amidating monooxygenase, an integral membrane peptide-processing enzyme.** *J Biol Chem* 1997, **272:**12667-12675.
278. Neukomm LJ, Zeng S, Frei AP, Huegli PA, Hengartner MO: **Small GTPase CDC-42 promotes apoptotic cell corpse clearance in response to PAT-2 and CED-1 in C. elegans.** *Cell Death Differ* 2014, **21:**845-853.
279. Haskins KA, Russell JF, Gaddis N, Dressman HK, Aballay A: **Unfolded protein response genes regulated by CED-1 are required for Caenorhabditis elegans innate immunity.** *Dev Cell* 2008, **15:**87-97.
280. Ster J, Steuble M, Orlando C, Diep TM, Akhmedov A, Raineteau O, Pernet V, Sonderegger P, Gerber U: **Calsyntenin-1 regulates targeting of dendritic NMDA receptors and dendritic spine maturation in CA1 hippocampal pyramidal cells during postnatal development.** *J Neurosci* 2014, **34:**8716-8727.
281. Warren DT, Zhang Q, Weissberg PL, Shanahan CM: **Nesprins: intracellular scaffolds that maintain cell architecture and coordinate cell function?** *Expert Rev Mol Med* 2005, **7:**1-15.
282. Zhang Q, Minaisah RM, Ferraro E, Li C, Porter LJ, Zhou C, Gao F, Zhang J, Rajgor D, Autore F, et al: **N-terminal nesprin-2 variants regulate beta-catenin signalling.** *Exp Cell Res* 2016, **345:**168-179.
283. Fontaine RH, Cases O, Lelievre V, Mesples B, Renauld JC, Loron G, Degos V, Dournaud P, Baud O, Gressens P: **IL-9/IL-9 receptor signaling selectively protects cortical neurons against developmental apoptosis.** *Cell Death Differ* 2008, **15:**1542-1552.
284. Castle CD, Cassimere EK, Denicourt C: **LAS1L interacts with the mammalian Rix1 complex to regulate ribosome biogenesis.** *Mol Biol Cell* 2012, **23:**716-728.
285. Pestov DG, Strezoska Z, Lau LF: **Evidence of p53-dependent cross-talk between ribosome biogenesis and the cell cycle: effects of nucleolar protein Bop1 on G(1)/S transition.** *Mol Cell Biol* 2001, **21:**4246-4255.
286. Castle CD, Cassimere EK, Lee J, Denicourt C: **Las1L is a nucleolar protein required for cell proliferation and ribosome biogenesis.** *Mol Cell Biol* 2010, **30:**4404-4414.
287. Mouysset J, Gilberto S, Meier MG, Lampert F, Belwal M, Meraldi P, Peter M: **CRL4(RBBP7) is required for efficient CENP-A deposition at centromeres.** *J Cell Sci* 2015, **128:**1732-1745.
288. Wennmann DO, Schmitz J, Wehr MC, Krahn MP, Koschmal N, Gromnitza S, Schulze U, Weide T, Chekuri A, Skryabin BV, et al: **Evolutionary and molecular facts link the WWC protein family to Hippo signaling.** *Mol Biol Evol* 2014, **31:**1710-1723.
289. Azzolin L, Panciera T, Soligo S, Enzo E, Bicciato S, Dupont S, Bresolin S, Frasson C, Basso G, Guzzardo V, et al: **YAP/TAZ incorporation in the beta-catenin destruction complex orchestrates the Wnt response.** *Cell* 2014, **158:**157-170.
290. Read MA, Brownell JE, Gladysheva TB, Hottelet M, Parent LA, Coggins MB, Pierce JW, Podust VN, Luo RS, Chau V, Palombella VJ: **Nedd8 modification of cul-1 activates SCF(beta(TrCP))-dependent ubiquitination of IkappaBalpha.** *Mol Cell Biol* 2000, **20:**2326-2333.
291. Winston JT, Strack P, Beer-Romero P, Chu CY, Elledge SJ, Harper JW: **The SCFbeta-TRCP-ubiquitin ligase complex associates specifically with phosphorylated destruction motifs in**

IkappaBalpha and beta-catenin and stimulates IkappaBalpha ubiquitination in vitro. *Genes Dev* 1999, **13:**270-283.

292. Liu HY, Meakin SO: **ShcB and ShcC activation by the Trk family of receptor tyrosine kinases.** *J Biol Chem* 2002, **277:**26046-26056.
293. Henne WM, Boucrot E, Meinecke M, Evergren E, Vallis Y, Mittal R, McMahon HT: **FCHo proteins are nucleators of clathrin-mediated endocytosis.** *Science* 2010, **328:**1281-1284.
294. Blitzer JT, Nusse R: **A critical role for endocytosis in Wnt signaling.** *BMC Cell Biol* 2006, **7:**28.
295. Das TK, Sangodkar J, Negre N, Narla G, Cagan RL: **Sin3a acts through a multi-gene module to regulate invasion in Drosophila and human tumors.** *Oncogene* 2013, **32:**3184-3197.
296. Sultana A, Nakaya N, Dong L, Abu-Asab M, Qian H, Tomarev SI: **Deletion of olfactomedin 2 induces changes in the AMPA receptor complex and impairs visual, olfactory, and motor functions in mice.** *Exp Neurol* 2014, **261:**802-811.
297. Hollmann M, Hartley M, Heinemann S: **Ca2+ permeability of KA-AMPA--gated glutamate receptor channels depends on subunit composition.** *Science* 1991, **252:**851-853.
298. Palacios F, Tushir JS, Fujita Y, D'Souza-Schorey C: **Lysosomal targeting of E-cadherin: a unique mechanism for the down-regulation of cell-cell adhesion during epithelial to mesenchymal transitions.** *Mol Cell Biol* 2005, **25:**389-402.
299. Toyoshima M, Tanaka N, Aoki J, Tanaka Y, Murata K, Kyuuma M, Kobayashi H, Ishii N, Yaegashi N, Sugamura K: **Inhibition of tumor growth and metastasis by depletion of vesicular sorting protein Hrs: its regulatory role on E-cadherin and beta-catenin.** *Cancer Res* 2007, **67:**5162-5171.
300. Zarnegar B, Yamazaki S, He JQ, Cheng G: **Control of canonical NF-kappaB activation through the NIK-IKK complex pathway.** *Proc Natl Acad Sci U S A* 2008, **105:**3503-3508.
301. Deng J, Miller SA, Wang HY, Xia W, Wen Y, Zhou BP, Li Y, Lin SY, Hung MC: **beta-catenin interacts with and inhibits NF-kappa B in human colon and breast cancer.** *Cancer Cell* 2002, **2:**323-334.
302. Li FQ, Mofunanya A, Harris K, Takemaru K: **Chibby cooperates with 14-3-3 to regulate beta-catenin subcellular distribution and signaling activity.** *J Cell Biol* 2008, **181:**1141-1154.
303. Carroll CW, Silva MC, Godek KM, Jansen LE, Straight AF: **Centromere assembly requires the direct recognition of CENP-A nucleosomes by CENP-N.** *Nat Cell Biol* 2009, **11:**896-902.
304. Peng YY, He YH, Chen C, Xu T, Li L, Ni MM, Meng XM, Huang C, Li J: **NLRC5 regulates cell proliferation, migration and invasion in hepatocellular carcinoma by targeting the Wnt/beta-catenin signaling pathway.** *Cancer Lett* 2016, **376:**10-21.
305. Meng Q, Cai C, Sun T, Wang Q, Xie W, Wang R, Cui J: **Reversible ubiquitination shapes NLRC5 function and modulates NF-kappaB activation switch.** *J Cell Biol* 2015, **211:**1025-1040.
306. Inohara N, Koseki T, del Peso L, Hu Y, Yee C, Chen S, Carrio R, Merino J, Liu D, Ni J, Nunez G: **Nod1, an Apaf-1-like activator of caspase-9 and nuclear factor-kappaB.** *J Biol Chem* 1999, **274:**14560-14567.
307. Yao L, Zhao H, Tang H, Liang J, Liu L, Dong H, Zou F, Cai S: **The receptor for advanced glycation end products is required for beta-catenin stabilization in a chemical-induced asthma model.** *Br J Pharmacol* 2016, **173:**2600-2613.
308. Tu M, Lu C, Lv N, Wei J, Lu Z, Xi C, Chen J, Guo F, Jiang K, Li Q, et al: **Vasohibin 2 promotes human luminal breast cancer angiogenesis in a non-paracrine manner via transcriptional activation of fibroblast growth factor 2.** *Cancer Lett* 2016, **383:**272-281.
309. Ruehr ML, Russell MA, Ferguson DG, Bhat M, Ma J, Damron DS, Scott JD, Bond M: **Targeting of protein kinase A by muscle A kinase-anchoring protein (mAKAP) regulates phosphorylation and function of the skeletal muscle ryanodine receptor.** *J Biol Chem* 2003, **278:**24831-24836.
310. Buzzi LI, Simonetta SH, Parodi AJ, Castro OA: **The two Caenorhabditis elegans UDP-glucose:glycoprotein glucosyltransferase homologues have distinct biological functions.** *PLoS One* 2011, **6:**e27025.

311. Hoyer-Hansen M, Jaattela M: **Connecting endoplasmic reticulum stress to autophagy by unfolded protein response and calcium.** *Cell Death Differ* 2007, **14:**1576-1582.
312. Abe S, Nagasaka K, Hirayama Y, Kozuka-Hata H, Oyama M, Aoyagi Y, Obuse C, Hirota T: **The initial phase of chromosome condensation requires Cdk1-mediated phosphorylation of the CAP-D3 subunit of condensin II.** *Genes Dev* 2011, **25:**863-874.
313. McKie AB, Vaughan S, Zanini E, Okon IS, Louis L, de Sousa C, Greene MI, Wang Q, Agarwal R, Shaposhnikov D, et al: **The OPCML tumor suppressor functions as a cell surface repressor-adaptor, negatively regulating receptor tyrosine kinases in epithelial ovarian cancer.** *Cancer Discov* 2012, **2:**156-171.
314. Han WD, Zhao YL, Meng YG, Zang L, Wu ZQ, Li Q, Si YL, Huang K, Ba JM, Morinaga H, et al: **Estrogenically regulated LRP16 interacts with estrogen receptor alpha and enhances the receptor's transcriptional activity.** *Endocr Relat Cancer* 2007, **14:**741-753.
315. Yang J, Zhao YL, Wu ZQ, Si YL, Meng YG, Fu XB, Mu YM, Han WD: **The single-macro domain protein LRP16 is an essential cofactor of androgen receptor.** *Endocr Relat Cancer* 2009, **16:**139-153.
316. Kouzmenko AP, Takeyama K, Ito S, Furutani T, Sawatsubashi S, Maki A, Suzuki E, Kawasaki Y, Akiyama T, Tabata T, Kato S: **Wnt/beta-catenin and estrogen signaling converge in vivo.** *J Biol Chem* 2004, **279:**40255-40258.
317. Kretzschmar K, Cottle DL, Schweiger PJ, Watt FM: **The Androgen Receptor Antagonizes Wnt/beta-Catenin Signaling in Epidermal Stem Cells.** *J Invest Dermatol* 2015, **135:**2753-2763.
318. Pearce LR, Huang X, Boudeau J, Pawlowski R, Wullschleger S, Deak M, Ibrahim AF, Gourlay R, Magnuson MA, Alessi DR: **Identification of Protor as a novel Rictor-binding component of mTOR complex-2.** *Biochem J* 2007, **405:**513-522.
319. Lu SC: **Regulation of glutathione synthesis.** *Mol Aspects Med* 2009, **30:**42-59.
320. Hayano M, Yang WS, Corn CK, Pagano NC, Stockwell BR: **Loss of cysteinyl-tRNA synthetase (CARS) induces the transsulfuration pathway and inhibits ferroptosis induced by cystine deprivation.** *Cell Death Differ* 2016, **23:**270-278.
321. Zhang T, Taylor J, Jiang Y, Pereyra AS, Messi ML, Wang ZM, Herenu C, Delbono O: **Troponin T3 regulates nuclear localization of the calcium channel Cavbeta1a subunit in skeletal muscle.** *Exp Cell Res* 2015, **336:**276-286.

Supplementary Figure 1

Interaction of miR-4673 with target transcripts.

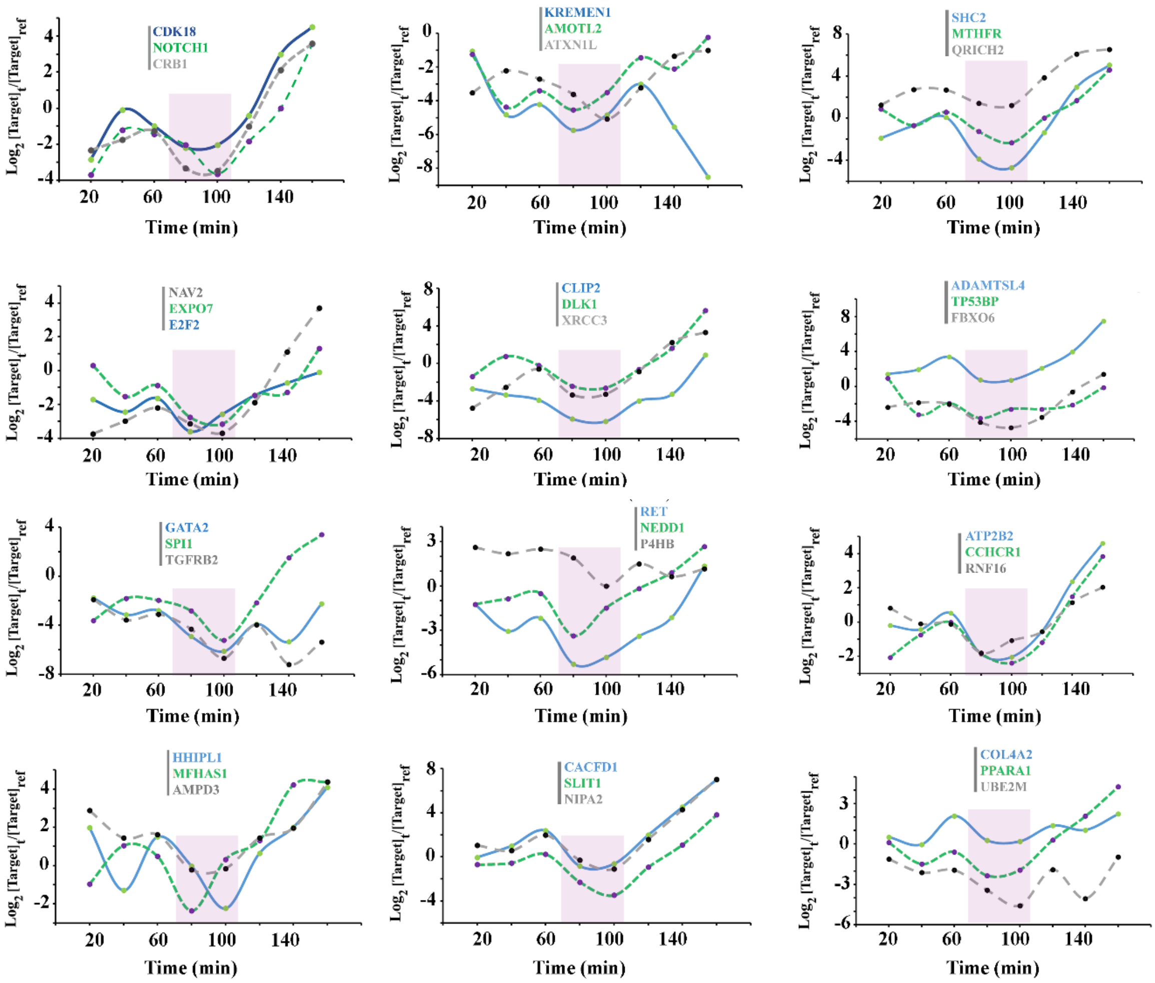

Graphs demonstrate expression levels of miR-4673 targets (summarised in Figure 7d of the main manuscript) at specific time points ([Target]$_t$) normalized to the expression levels of the same genes at time zero ([target]$_{ref}$) on a logarithmic basis. Purple demonstrated time points that correspond to the temporal window that accommodate the transcriptional profile of M phase.

Supplementary Figure 2

Identification of a mouse genic cluster with miR$_{HR}$ signature.

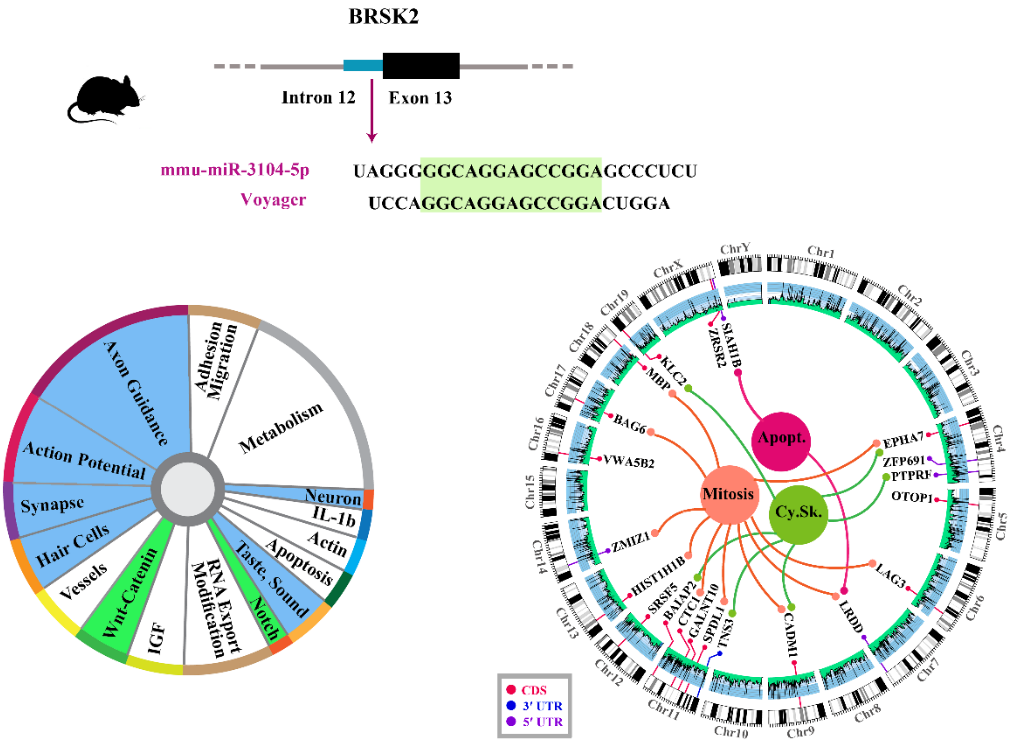

In mouse, miR$_{HR}$ is targeted by mmu-miR-3104 encoded in intron 12 of BRSK2 locus (top). The putative interactome is distributed in GC-rich isochores and ontology analysis demonstrates involvement of the target gens in neurogenesis similar to the interactome of miR-4673.

Supplementary Discussion 2

Targets identified as potential interactome of miR-4673 in *Gallus gallus* are involved in the regulation of canonical and non-canonical Wnt cascades as follows:

VASH2: Vasohibin-2 downregulates p53[1] that in turn accelerates degradation of catenin-β1[2]. Vasohibin-2 also promotes phosphorylation of Smad-2 and Smad-2[3] that are required for nuclear translocation of catenin-β1[4]. Smads also control transcriptional elongation from genes activated by catenin-β1[5]. Further, vasohibin-2 activates FGF-2[6]. FGF signaling decreases autophagy and enhances the activity of catenin-β1 as stated previously.

MEIS1: Meis1 enhances transcription of the CDK inhibitor p21[7]. In contrast, catenin-β1 downregulates expression of p21[8]. This interaction controls proliferative equilibrium in the context of cell-cell adhesion[8].

PLCG1: This protein is required for Ca^{2+} flux into cells[9]. Also PLC-γ1 catalyses hydrolysis of phosphatidylinositol4,5-P_2 to inositol 1,4,5-trisphosphate and DAG leading to increased intracellular Ca^{2+} and activation of protein kinase C (PKC). Activation of PKC by DAG[10] triggers phosphorylation and subsequent inactivation of GSK3β[11]. The latter interaction is enhanced by Akt-dependent activation of PLCG1[12].

DACT2: This protein inhibits Wnt signalling by disrupting association of catenin-β1 and LEF1 and also enhancing association of cytoplasmic catenin-β1 and E-cadherin in junctional complexes[13].

FRK: This protein (alias: RAK) regulates stability and activity of PTEN[14] that in turn inhibits PI3k cascade[15] and activates GSK3β by inhibiting AKT and Integrin-linked kinase[16].

AP180: binds to the carboxyl-terminal SH2 domain of PLCG1 and inhibits its enzymatic activity[17]. The activities of PLCG1 were addressed previously.

LPIN1: is a Mg^{2+}-dependent phosphatidate phosphatase that catalyzes the dephosphorylation of PA to DAG and phosphate[18, 19].

PTCHD4: suppresses Hedghog signalling downstream of p53[20]. Signalling by Hedgehog cascade antagonizes transcriptional activity of catenin-β1[21].

EFEMP1: This protein activates AKT and MAPK by binding to and activating EGFR[22].

NECAB2: is a neuronal calcium binding protein that associates with and mediates activation of AKT/MAPK by metabotropic glutamate receptor type-5[23].

ZNF423: This protein (alias: OAZ) associates with Smads downstream of BMP2 signalling[24]. Smads (Smad2 and Smad4) are required for nuclear translocation of catenin-β1[4]. Smads also control transcriptional elongation from genes activated by catenin-β1[5].

ACSF3: This protein is a malonyl-CoA synthetase involved in the synthesis of fatty acids in mitochondria[25]. Malonate modulates generation of reactive oxygen species in the mitochondria[26]. Reactive oxygen species in turn control thioredoxin activity that upon redox-dependent dissociation from Dvl, provides a stable pool of cytosolic catenin-β1[27, 28].

SNTB2: β-synthrophin interacts with Tiam1 and Rac and localizes the latter proteins to cell-cell junctions[29]. Enhancement of adherence junctions by Tiam1 decreases availability of cytosolic free catenin-β1[30]. Syntrophin also activates PI3K/AKT in complex with integrins[31].

CELSR1: A cadherin-type receptor that is reported to block Wnt signalling[32].

SMO: This protein (Smoothened) is an upstream component of the Sonic Hedgehog pathway[33]. Signalling by Hedgehog cascade antagonizes transcriptional activity of catenin-β1[21].

FSTL1: This protein is a BMP4 antagonist[34] and signalling by the latter protein in turn antagonizes Wnt activity[35].

TUBA1A: This protein (tubulin Alpha 1a) is a major component of microtubules. Microtubules are involved in supporting and regulating distribution and local concentration of E-cadherin[36] that indirectly controls catenin-β1 spatial distribution.

CFTR: is a chloride channel that regulates membrane potential[37] and hence voltage-gated calcium channel activity.

CACNA1C: encodes an L-type voltage-sensitive calcium channel[38].

ATG3: is involved in upregulation of autophagy[39] that in turn antagonizes Wnt signalling[40].

ARHGAP6: GTPase activator for the Rho[41] that activates phospholipase C in PI3K cascade[42].

NAXD: This protein (alias: CARKD) is a component of the NAPDHX repair enzyme[43]. NADPHX is generated through activity of uncoupling agents in the inner mitochondrial membrane[44].

HIKESHI: A nuclear importer for HSP70s during heat shock stress[45].

AHCYL2: regulates electrogenic Na(+)-HCO3(-) cotransporter NBCe1-B[46] and hence controls intracellular pH that in turn modulates secretion of Wnt inhibitor Dkk1[47].

CDC123: Cdc123 facilitates the biogenesis of eIF2 by catalysing assembly of eIF2γ to eIF2α[48]. Activity of eIF2α modulates autophagy[49] and hence indirectly regulates Wnt signalling[40].

DHX30: is involved in biogenesis of mitochondrial ribosomes[50]. Ribosomes are only essential for transcription of genes that encode proteins of the electron transport chain[51] and hence the energetic landscape of mitochondria and ROS generation.

TPK1: The protein encoded by this gene is a thiamine pyrophosphokinase[52] that catalyses production of thiamine pyrophosphate required by various mitochondrial enzymes, e.g. pyruvate dehydrogenase[53].

CSPG5: interacts with Golgi-associated PIST (alias: GOPC)[54] that in turn modulates autophagy[55] and trafficking of Frizzled to the cell membrane[56].

UBP1: modulates the transcription of CYP11A1[57] that in turn catalyses conversion of cholesterol into steroids. Steroids regulate transcription of mitochondrial genes[58] that encode proteins of the electron transport chain[51].

ADCY2/ADCY8: Members of the family of adenylate cyclases that catalyse production of cAMP that in turn activates PKA with concomitant phosphorylation-dependent stabilization of catenin-β1[59].

RASGEF1C: is a Rap2 specific activator[60]. Rap2 stabilizes catenin-β1[61].

WNT8C: is a Wnt ligand.

PDLIM4: suppresses Src kinase activation[62] . In turn Src kinase activates RhoGAP[63] and promotes inactivation of CDC42, Rho and Rac.

GRIN2A: is subunit 2A of Ionotropic glutamate receptor that triggers calcium influx.

AXIN1: is an essential component of a catenin destruction complex[64].

ASTN2: In combination with frizzled-6, a negative regulator of Wnt signalling[65], ASTN2 regulates planar cell polarity[66].

AIF1L: This protein (alias: Iba2) mediates F-actin cross-linking activity[67].

NACC2: this protein (alias: RBB) acts as a transcriptional repressor of MDM2 that subsequently stabilizes p53[68]. Stabilized p53 accelerates degradation of catenin-β1[2].

TCF4: A transcriptional repressor and binding partner of catenin-β1.

PRICKLE2: is a key component of PCP-related non-canonical Wnt signalling[69].

TPRA1: This protein (alias: GPR175) inhibits PKA activity and amplifies Hedgehog signalling[70], leading to dephosphorylation-dependent destabilization of catenin-β1[59] and antagonizing transcriptional activity of catenin-β1[21].

GRM7: A G-protein coupled glutamate receptor that reduces cAMP[71]. Reduction of cAMP inactivates PKA with concomitant dephosphorylation-dependent destabilization of catenin-β1[59].

PTPRG: belongs to protein tyrosine phosphatase family that binds to and modulates phosphorylation level of catenin-β1[72].

WNK2: Wnk kinases are positive regulators of Wnt/Catenin signalling[73].

STK10: is a polo-like kinase kinase[74]. Plk directly[75] and indirectly[76] enhances phosphorylation of catenin-β1 and its stabilization.

ACAP2: is a GTPase-activating protein for Arf6[77] and the latter protein in turn liberates catenin-β1 from N-cadherin and increases cytoplasmic availability of catenin-β1[78] during Wnt signalling.

RERE: this protein (alias: Atrophin-2) interacts with atypical cadherin Fat1 in cell-cell junctions[79]. Fat-1 sequesters catenin-β1 in the junctional complex and fine-tunes Wnt signalling[80].

CDH4: is a calcium-dependent cadherin that sequesters catenin-β1 in the junctional complex[81].

NTSR1: A neurotensin receptor that activates phospholipase C[82] and the latter protein in turn catalyses hydrolysis of phosphatidylinositol4,5-P_2 to inositol 1,4,5-trisphosphate and DAG leading to increased intracellular Ca^{2+} and activation of protein kinase C. Activation of PKC by DAG[10] triggers phosphorylation and subsequent inactivation of GSK3β[11]. The latter interaction is enhanced by Akt-dependent activation of PLCG1[12].

CBFA2T2: This protein (alias: p85) is the regulatory subunit of Class I PI 3′-kinases[83].

OPHN1: It encodes a RhoGAP protein[84] that controls activity of Rho and the latter protein in turn controls stability of adherence junctions[85] to sequester catenin-β1.

GABRA3: Gamma-Aminobutyric Acid Type A Receptor Alpha3 Subunit that forms an anion channel and upon activation hyperpolarizes the cell membrane and inactivates voltage-gated calcium channels[86].

IL1RAPL2 : A close homologue of this protein, IL1RAPL1, associates with NCS-1 and inhibits N-type voltage-gated calcium channels (N-VGCC)[87].

UNC5C: is a netrin receptor[88] that functions parallel to and fine-tunes Wnt signalling[89].

TALDO1: encodes a key enzyme of the non-oxidative pentose phosphate pathway that maintains the level of reduced Glutathione[90] and hence regulates ROS activity. Reactive oxygen species in turn control thioredoxin activity that upon redox-dependent dissociation from Dvl, provides a stable pool of cytosolic catenin-β1[27, 28].

KCNMA1: the protein encodes high-conductance voltage- and calcium-activated K^+ channels that open in response to calcium influx and hyperpolarize the cell membrane and close voltage-dependent Ca^{2+} channels[91].

RGS10: The encoded protein acts as a selective activator of inhibitory G-protein αi GTPase activity[92].

GLRX3: This protein (alias: ERp16) relieves endoplasmic reticulum stress through disulfide isomerization[93]. Autophagy that follows endoplasmic reticulum stress[94] reduces cytosolic availability of catenin-β1[95].

MLYK: acts downstream of and in parallel to catenin-β1 signalling[96].

RXRG: is a retinoic acid receptor that inhibits Wnt signalling[97].

SEC16B: is an endoplasmic reticulum protein critical for secretion of CFTR[98] that in turn controls chloride and bicarbonate concentration and activity of soluble adenylyl cyclase[99].

DGKD: A diacylglycerol kinase that generates phosphtidic acid that in turn activates mTOR signalling[100] to inhibit autophagy and degradation of catenin-β1[95].

FARP2: is a guanine nucleotide exchange factor that activates CDC42[101] and the latter protein in turn inactivates GSK3β.

PIK3R5: This protein (alias: p101) is the regulatory subunit of PI3K[102].

MAP2K1: Dual specificity protein kinase that is central to activity of MAPK pathway. Downstream of MAPK signalling, JNK phosphorylates and promotes degradation of catenin-β1[103].

SLC9A1: This protein (alias: NHE1) regulates intracellular pH[104, 105] that in turn modulates secretion of Wnt inhibitor Dkk1[47].

GRIK3: Glutamate ionotropic receptor kainate type subunit 3 that inhibits calcium flux through P/Q-type Ca^{2+} channels[106].

COL16A1: associates with cell surface integrin-α1β1 in focal adhesions[107] that in turn activate integrin-linked kinase[108]. Integrin-linked kinase inhibits GSK3β by phosphorylation of serin-9[109]. Inhibition of GSK3β increases the level of free cytoplasmic catenin-β1.

TFAP2E: is a potent upstream inducer of Wnt antagonist DKK4[110].

CACNA1G: is a low voltage-activated T-type calcium channel[111].

PRKCA: is a calcium-activated, phospholipid- and diacylglycerol (DAG)-dependent serine/threonine-protein kinase that counteracts Wnt signalling by phosphorylating catenin-β1[112].

DDX5: associates with and promotes nuclear translocation of catenin-β1[113].

References

1. Li Z, Tu M, Han B, Gu Y, Xue X, Sun J, Ge Q, Miao Y, Qian Z, Gao W: **Vasohibin 2 decreases the cisplatin sensitivity of hepatocarcinoma cell line by downregulating p53.** *PLoS One* 2014, **9:**e90358.
2. Sadot E, Geiger B, Oren M, Ben-Ze'ev A: **Down-regulation of beta-catenin by activated p53.** *Mol Cell Biol* 2001, **21:**6768-6781.
3. Norita R, Suzuki Y, Furutani Y, Takahashi K, Yoshimatsu Y, Podyma-Inoue KA, Watabe T, Sato Y: **Vasohibin-2 is required for epithelial-mesenchymal transition of ovarian cancer cells by modulating transforming growth factor-beta signaling.** *Cancer Sci* 2017, **108:**419-426.
4. Jian H, Shen X, Liu I, Semenov M, He X, Wang XF: **Smad3-dependent nuclear translocation of beta-catenin is required for TGF-beta1-induced proliferation of bone marrow-derived adult human mesenchymal stem cells.** *Genes Dev* 2006, **20:**666-674.
5. Estaras C, Benner C, Jones KA: **SMADs and YAP compete to control elongation of beta-catenin:LEF-1-recruited RNAPII during hESC differentiation.** *Mol Cell* 2015, **58:**780-793.
6. Tu M, Lu C, Lv N, Wei J, Lu Z, Xi C, Chen J, Guo F, Jiang K, Li Q, et al: **Vasohibin 2 promotes human luminal breast cancer angiogenesis in a non-paracrine manner via transcriptional activation of fibroblast growth factor 2.** *Cancer Lett* 2016, **383:**272-281.
7. Mahmoud AI, Kocabas F, Muralidhar SA, Kimura W, Koura AS, Thet S, Porrello ER, Sadek HA: **Meis1 regulates postnatal cardiomyocyte cell cycle arrest.** *Nature* 2013, **497:**249-253.
8. Kamei J, Toyofuku T, Hori M: **Negative regulation of p21 by beta-catenin/TCF signaling: a novel mechanism by which cell adhesion molecules regulate cell proliferation.** *Biochem Biophys Res Commun* 2003, **312:**380-387.
9. Patterson RL, van Rossum DB, Ford DL, Hurt KJ, Bae SS, Suh PG, Kurosaki T, Snyder SH, Gill DL: **Phospholipase C-gamma is required for agonist-induced Ca2+ entry.** *Cell* 2002, **111:**529-541.
10. Huang KP: **The mechanism of protein kinase C activation.** *Trends Neurosci* 1989, **12:**425-432.
11. Goode N, Hughes K, Woodgett JR, Parker PJ: **Differential regulation of glycogen synthase kinase-3 beta by protein kinase C isotypes.** *J Biol Chem* 1992, **267:**16878-16882.
12. Wang Y, Wu J, Wang Z: **Akt binds to and phosphorylates phospholipase C-gamma1 in response to epidermal growth factor.** *Mol Biol Cell* 2006, **17:**2267-2277.
13. Wang S, Dong Y, Zhang Y, Wang X, Xu L, Yang S, Li X, Dong H, Xu L, Su L, et al: **DACT2 is a functional tumor suppressor through inhibiting Wnt/beta-catenin pathway and associated with poor survival in colon cancer.** *Oncogene* 2015, **34:**2575-2585.

14. Yim EK, Peng G, Dai H, Hu R, Li K, Lu Y, Mills GB, Meric-Bernstam F, Hennessy BT, Craven RJ, Lin SY: **Rak functions as a tumor suppressor by regulating PTEN protein stability and function.** *Cancer Cell* 2009, **15:**304-314.
15. Carracedo A, Pandolfi PP: **The PTEN-PI3K pathway: of feedbacks and cross-talks.** *Oncogene* 2008, **27:**5527-5541.
16. Mulholland DJ, Dedhar S, Wu H, Nelson CC: **PTEN and GSK3beta: key regulators of progression to androgen-independent prostate cancer.** *Oncogene* 2006, **25:**329-337.
17. Han SJ, Lee JH, Hong SH, Park SD, Kim CG, Song MD, Park TK, Kim CG: **AP180 binds to the C-terminal SH2 domain of phospholipase C-gamma1 and inhibits its enzymatic activity.** *Biochem Biophys Res Commun* 2002, **290:**35-41.
18. Grimsey N, Han GS, O'Hara L, Rochford JJ, Carman GM, Siniossoglou S: **Temporal and spatial regulation of the phosphatidate phosphatases lipin 1 and 2.** *J Biol Chem* 2008, **283:**29166-29174.
19. Han GS, Wu WI, Carman GM: **The Saccharomyces cerevisiae Lipin homolog is a Mg2+-dependent phosphatidate phosphatase enzyme.** *J Biol Chem* 2006, **281:**9210-9218.
20. Chung JH, Larsen AR, Chen E, Bunz F: **A PTCH1 homolog transcriptionally activated by p53 suppresses Hedgehog signaling.** *J Biol Chem* 2014, **289:**33020-33031.
21. Schneider FT, Schanzer A, Czupalla CJ, Thom S, Engels K, Schmidt MH, Plate KH, Liebner S: **Sonic hedgehog acts as a negative regulator of {beta}-catenin signaling in the adult tongue epithelium.** *Am J Pathol* 2010, **177:**404-414.
22. Camaj P, Seeliger H, Ischenko I, Krebs S, Blum H, De Toni EN, Faktorova D, Jauch KW, Bruns CJ: **EFEMP1 binds the EGF receptor and activates MAPK and Akt pathways in pancreatic carcinoma cells.** *Biol Chem* 2009, **390:**1293-1302.
23. Canela L, Fernandez-Duenas V, Albergaria C, Watanabe M, Lluis C, Mallol J, Canela EI, Franco R, Lujan R, Ciruela F: **The association of metabotropic glutamate receptor type 5 with the neuronal Ca2+-binding protein 2 modulates receptor function.** *J Neurochem* 2009, **111:**555-567.
24. Hata A, Seoane J, Lagna G, Montalvo E, Hemmati-Brivanlou A, Massague J: **OAZ uses distinct DNA- and protein-binding zinc fingers in separate BMP-Smad and Olf signaling pathways.** *Cell* 2000, **100:**229-240.
25. Witkowski A, Thweatt J, Smith S: **Mammalian ACSF3 protein is a malonyl-CoA synthetase that supplies the chain extender units for mitochondrial fatty acid synthesis.** *J Biol Chem* 2011, **286:**33729-33736.
26. Fernandez-Gomez FJ, Galindo MF, Gomez-Lazaro M, Yuste VJ, Comella JX, Aguirre N, Jordan J: **Malonate induces cell death via mitochondrial potential collapse and delayed swelling through an ROS-dependent pathway.** *Br J Pharmacol* 2005, **144:**528-537.
27. Funato Y, Michiue T, Asashima M, Miki H: **The thioredoxin-related redox-regulating protein nucleoredoxin inhibits Wnt-beta-catenin signalling through dishevelled.** *Nat Cell Biol* 2006, **8:**501-508.
28. Funato Y, Terabayashi T, Sakamoto R, Okuzaki D, Ichise H, Nojima H, Yoshida N, Miki H: **Nucleoredoxin sustains Wnt/beta-catenin signaling by retaining a pool of inactive dishevelled protein.** *Curr Biol* 2010, **20:**1945-1952.
29. Mack NA, Porter AP, Whalley HJ, Schwarz JP, Jones RC, Khaja AS, Bjartell A, Anderson KI, Malliri A: **beta2-syntrophin and Par-3 promote an apicobasal Rac activity gradient at cell-cell junctions by differentially regulating Tiam1 activity.** *Nat Cell Biol* 2012, **14:**1169-1180.
30. Malliri A, van Es S, Huveneers S, Collard JG: **The Rac exchange factor Tiam1 is required for the establishment and maintenance of cadherin-based adhesions.** *J Biol Chem* 2004, **279:**30092-30098.
31. Xiong Y, Zhou Y, Jarrett HW: **Dystrophin glycoprotein complex-associated Gbetagamma subunits activate phosphatidylinositol-3-kinase/Akt signaling in skeletal muscle in a laminin-dependent manner.** *J Cell Physiol* 2009, **219:**402-414.
32. Morgan R, El-Kadi AM, Theokli C: **Flamingo, a cadherin-type receptor involved in the Drosophila planar polarity pathway, can block signaling via the canonical wnt pathway in Xenopus laevis.** *Int J Dev Biol* 2003, **47:**245-252.

33. Chen W, Ren XR, Nelson CD, Barak LS, Chen JK, Beachy PA, de Sauvage F, Lefkowitz RJ: **Activity-dependent internalization of smoothened mediated by beta-arrestin 2 and GRK2.** *Science* 2004, **306:**2257-2260.
34. Geng Y, Dong Y, Yu M, Zhang L, Yan X, Sun J, Qiao L, Geng H, Nakajima M, Furuichi T, et al: **Follistatin-like 1 (Fstl1) is a bone morphogenetic protein (BMP) 4 signaling antagonist in controlling mouse lung development.** *Proc Natl Acad Sci U S A* 2011, **108:**7058-7063.
35. Ille F, Atanasoski S, Falk S, Ittner LM, Marki D, Buchmann-Moller S, Wurdak H, Suter U, Taketo MM, Sommer L: **Wnt/BMP signal integration regulates the balance between proliferation and differentiation of neuroepithelial cells in the dorsal spinal cord.** *Dev Biol* 2007, **304:**394-408.
36. Stehbens SJ, Paterson AD, Crampton MS, Shewan AM, Ferguson C, Akhmanova A, Parton RG, Yap AS: **Dynamic microtubules regulate the local concentration of E-cadherin at cell-cell contacts.** *J Cell Sci* 2006, **119:**1801-1811.
37. Sheppard DN, Welsh MJ: **Structure and function of the CFTR chloride channel.** *Physiol Rev* 1999, **79:**S23-45.
38. Hofmann F, Flockerzi V, Kahl S, Wegener JW: **L-type CaV1.2 calcium channels: from in vitro findings to in vivo function.** *Physiol Rev* 2014, **94:**303-326.
39. Yoo BH, Zagryazhskaya A, Li Y, Koomson A, Khan IA, Sasazuki T, Shirasawa S, Rosen KV: **Upregulation of ATG3 contributes to autophagy induced by the detachment of intestinal epithelial cells from the extracellular matrix, but promotes autophagy-independent apoptosis of the attached cells.** *Autophagy* 2015, **11:**1230-1246.
40. Gao C, Cao W, Bao L, Zuo W, Xie G, Cai T, Fu W, Zhang J, Wu W, Zhang X, Chen YG: **Autophagy negatively regulates Wnt signalling by promoting Dishevelled degradation.** *Nat Cell Biol* 2010, **12:**781-790.
41. Prakash SK, Paylor R, Jenna S, Lamarche-Vane N, Armstrong DL, Xu B, Mancini MA, Zoghbi HY: **Functional analysis of ARHGAP6, a novel GTPase-activating protein for RhoA.** *Hum Mol Genet* 2000, **9:**477-488.
42. Ochocka AM, Grden M, Sakowicz-Burkiewicz M, Szutowicz A, Pawelczyk T: **Regulation of phospholipase C-delta1 by ARGHAP6, a GTPase-activating protein for RhoA: possible role for enhanced activity of phospholipase C in hypertension.** *Int J Biochem Cell Biol* 2008, **40:**2264-2273.
43. Marbaix AY, Tyteca D, Niehaus TD, Hanson AD, Linster CL, Van Schaftingen E: **Occurrence and subcellular distribution of the NADPHX repair system in mammals.** *Biochem J* 2014, **460:**49-58.
44. Nedergaard J, Ricquier D, Kozak LP: **Uncoupling proteins: current status and therapeutic prospects.** *EMBO Rep* 2005, **6:**917-921.
45. Kose S, Furuta M, Imamoto N: **Hikeshi, a nuclear import carrier for Hsp70s, protects cells from heat shock-induced nuclear damage.** *Cell* 2012, **149:**578-589.
46. Yamaguchi S, Ishikawa T: **AHCYL2 (long-IRBIT) as a potential regulator of the electrogenic Na(+)-HCO3(-) cotransporter NBCe1-B.** *FEBS Lett* 2014, **588:**672-677.
47. Lyros O, Rafiee P, Nie L, Medda R, Jovanovic N, Schmidt J, Mackinnon A, Venu N, Shaker R: **Dickkopf-1, the Wnt antagonist, is induced by acidic pH and mediates epithelial cellular senescence in human reflux esophagitis.** *Am J Physiol Gastrointest Liver Physiol* 2014, **306:**G557-574.
48. Panvert M, Dubiez E, Arnold L, Perez J, Mechulam Y, Seufert W, Schmitt E: **Cdc123, a Cell Cycle Regulator Needed for eIF2 Assembly, Is an ATP-Grasp Protein with Unique Features.** *Structure* 2015, **23:**1596-1608.
49. B'Chir W, Maurin AC, Carraro V, Averous J, Jousse C, Muranishi Y, Parry L, Stepien G, Fafournoux P, Bruhat A: **The eIF2alpha/ATF4 pathway is essential for stress-induced autophagy gene expression.** *Nucleic Acids Res* 2013, **41:**7683-7699.
50. Antonicka H, Shoubridge EA: **Mitochondrial RNA Granules Are Centers for Posttranscriptional RNA Processing and Ribosome Biogenesis.** *Cell Rep* 2015.
51. Johnston IG, Williams BP: **Evolutionary Inference across Eukaryotes Identifies Specific Pressures Favoring Mitochondrial Gene Retention.** *Cell Syst* 2016, **2:**101-111.

52. Nosaka K, Onozuka M, Kakazu N, Hibi S, Nishimura H, Nishino H, Abe T: **Isolation and characterization of a human thiamine pyrophosphokinase cDNA.** *Biochim Biophys Acta* 2001, **1517:**293-297.
53. Hohmann S, Meacock PA: **Thiamin metabolism and thiamin diphosphate-dependent enzymes in the yeast Saccharomyces cerevisiae: genetic regulation.** *Biochim Biophys Acta* 1998, **1385:**201-219.
54. Hassel B, Schreff M, Stube EM, Blaich U, Schumacher S: **CALEB/NGC interacts with the Golgi-associated protein PIST.** *J Biol Chem* 2003, **278:**40136-40143.
55. Yue Z, Horton A, Bravin M, DeJager PL, Selimi F, Heintz N: **A novel protein complex linking the delta 2 glutamate receptor and autophagy: implications for neurodegeneration in lurcher mice.** *Neuron* 2002, **35:**921-933.
56. Yao R, Maeda T, Takada S, Noda T: **Identification of a PDZ domain containing Golgi protein, GOPC, as an interaction partner of frizzled.** *Biochem Biophys Res Commun* 2001, **286:**771-778.
57. Guo IC, Shih MC, Lan HC, Hsu NC, Hu MC, Chung BC: **Transcriptional regulation of human CYP11A1 in gonads and adrenals.** *J Biomed Sci* 2007, **14:**509-515.
58. Demonacos CV, Karayanni N, Hatzoglou E, Tsiriyiotis C, Spandidos DA, Sekeris CE: **Mitochondrial genes as sites of primary action of steroid hormones.** *Steroids* 1996, **61:**226-232.
59. Hino S, Tanji C, Nakayama KI, Kikuchi A: **Phosphorylation of beta-catenin by cyclic AMP-dependent protein kinase stabilizes beta-catenin through inhibition of its ubiquitination.** *Mol Cell Biol* 2005, **25:**9063-9072.
60. Pannekoek WJ, Linnemann JR, Brouwer PM, Bos JL, Rehmann H: **Rap1 and Rap2 antagonistically control endothelial barrier resistance.** *PLoS One* 2013, **8:**e57903.
61. Choi SC, Han JK: **Rap2 is required for Wnt/beta-catenin signaling pathway in Xenopus early development.** *EMBO J* 2005, **24:**985-996.
62. Zhang Y, Tu Y, Zhao J, Chen K, Wu C: **Reversion-induced LIM interaction with Src reveals a novel Src inactivation cycle.** *J Cell Biol* 2009, **184:**785-792.
63. Brouns MR, Matheson SF, Settleman J: **p190 RhoGAP is the principal Src substrate in brain and regulates axon outgrowth, guidance and fasciculation.** *Nat Cell Biol* 2001, **3:**361-367.
64. Nakamura T, Hamada F, Ishidate T, Anai K, Kawahara K, Toyoshima K, Akiyama T: **Axin, an inhibitor of the Wnt signalling pathway, interacts with beta-catenin, GSK-3beta and APC and reduces the beta-catenin level.** *Genes Cells* 1998, **3:**395-403.
65. Golan T, Yaniv A, Bafico A, Liu G, Gazit A: **The human Frizzled 6 (HFz6) acts as a negative regulator of the canonical Wnt. beta-catenin signaling cascade.** *J Biol Chem* 2004, **279:**14879-14888.
66. Chang H, Cahill H, Smallwood PM, Wang Y, Nathans J: **Identification of Astrotactin2 as a Genetic Modifier That Regulates the Global Orientation of Mammalian Hair Follicles.** *PLoS Genet* 2015, **11:**e1005532.
67. Schulze JO, Quedenau C, Roske Y, Adam T, Schuler H, Behlke J, Turnbull AP, Sievert V, Scheich C, Mueller U, et al: **Structural and functional characterization of human Iba proteins.** *FEBS J* 2008, **275:**4627-4640.
68. Xuan C, Wang Q, Han X, Duan Y, Li L, Shi L, Wang Y, Shan L, Yao Z, Shang Y: **RBB, a novel transcription repressor, represses the transcription of HDM2 oncogene.** *Oncogene* 2013, **32:**3711-3721.
69. Veeman MT, Slusarski DC, Kaykas A, Louie SH, Moon RT: **Zebrafish prickle, a modulator of noncanonical Wnt/Fz signaling, regulates gastrulation movements.** *Curr Biol* 2003, **13:**680-685.
70. Singh J, Wen X, Scales SJ: **The Orphan G Protein-coupled Receptor Gpr175 (Tpra40) Enhances Hedgehog Signaling by Modulating cAMP Levels.** *J Biol Chem* 2015, **290:**29663-29675.
71. Millan C, Lujan R, Shigemoto R, Sanchez-Prieto J: **The inhibition of glutamate release by metabotropic glutamate receptor 7 affects both [Ca2+]c and cAMP: evidence for a strong reduction of Ca2+ entry in single nerve terminals.** *J Biol Chem* 2002, **277:**14092-14101.

72. Meng K, Rodriguez-Pena A, Dimitrov T, Chen W, Yamin M, Noda M, Deuel TF: **Pleiotrophin signals increased tyrosine phosphorylation of beta beta-catenin through inactivation of the intrinsic catalytic activity of the receptor-type protein tyrosine phosphatase beta/zeta.** *Proc Natl Acad Sci U S A* 2000, **97:**2603-2608.
73. Serysheva E, Berhane H, Grumolato L, Demir K, Balmer S, Bodak M, Boutros M, Aaronson S, Mlodzik M, Jenny A: **Wnk kinases are positive regulators of canonical Wnt/beta-catenin signalling.** *EMBO Rep* 2013, **14:**718-725.
74. Walter SA, Cutler RE, Jr., Martinez R, Gishizky M, Hill RJ: **Stk10, a new member of the polo-like kinase kinase family highly expressed in hematopoietic tissue.** *J Biol Chem* 2003, **278:**18221-18228.
75. Arai T, Haze K, Iimura-Morita Y, Machida T, Iida M, Tanaka K, Komatani H: **Identification of beta-catenin as a novel substrate of Polo-like kinase 1.** *Cell Cycle* 2008, **7:**3556-3563.
76. Mbom BC, Siemers KA, Ostrowski MA, Nelson WJ, Barth AI: **Nek2 phosphorylates and stabilizes beta-catenin at mitotic centrosomes downstream of Plk1.** *Mol Biol Cell* 2014, **25:**977-991.
77. Jackson TR, Brown FD, Nie Z, Miura K, Foroni L, Sun J, Hsu VW, Donaldson JG, Randazzo PA: **ACAPs are arf6 GTPase-activating proteins that function in the cell periphery.** *J Cell Biol* 2000, **151:**627-638.
78. Grossmann AH, Yoo JH, Clancy J, Sorensen LK, Sedgwick A, Tong Z, Ostanin K, Rogers A, Grossmann KF, Tripp SR, et al: **The small GTPase ARF6 stimulates beta-catenin transcriptional activity during WNT5A-mediated melanoma invasion and metastasis.** *Sci Signal* 2013, **6:**ra14.
79. Hou R, Sibinga NE: **Atrophin proteins interact with the Fat1 cadherin and regulate migration and orientation in vascular smooth muscle cells.** *J Biol Chem* 2009, **284:**6955-6965.
80. Morris LG, Kaufman AM, Gong Y, Ramaswami D, Walsh LA, Turcan S, Eng S, Kannan K, Zou Y, Peng L, et al: **Recurrent somatic mutation of FAT1 in multiple human cancers leads to aberrant Wnt activation.** *Nat Genet* 2013, **45:**253-261.
81. Slater SC, Koutsouki E, Jackson CL, Bush RC, Angelini GD, Newby AC, George SJ: **R-cadherin:beta-catenin complex and its association with vascular smooth muscle cell proliferation.** *Arterioscler Thromb Vasc Biol* 2004, **24:**1204-1210.
82. Hermans E, Maloteaux JM, Octave JN: **Phospholipase C activation by neurotensin and neuromedin N in Chinese hamster ovary cells expressing the rat neurotensin receptor.** *Brain Res Mol Brain Res* 1992, **15:**332-338.
83. Backer JM, Myers MG, Jr., Shoelson SE, Chin DJ, Sun XJ, Miralpeix M, Hu P, Margolis B, Skolnik EY, Schlessinger J, et al.: **Phosphatidylinositol 3'-kinase is activated by association with IRS-1 during insulin stimulation.** *EMBO J* 1992, **11:**3469-3479.
84. Billuart P, Bienvenu T, Ronce N, des Portes V, Vinet MC, Zemni R, Roest Crollius H, Carrie A, Fauchereau F, Cherry M, et al: **Oligophrenin-1 encodes a rhoGAP protein involved in X-linked mental retardation.** *Nature* 1998, **392:**923-926.
85. Sahai E, Marshall CJ: **ROCK and Dia have opposing effects on adherens junctions downstream of Rho.** *Nat Cell Biol* 2002, **4:**408-415.
86. Marlin JJ, Carter AG: **GABA-A receptor inhibition of local calcium signaling in spines and dendrites.** *J Neurosci* 2014, **34:**15898-15911.
87. Gambino F, Pavlowsky A, Begle A, Dupont JL, Bahi N, Courjaret R, Gardette R, Hadjkacem H, Skala H, Poulain B, et al: **IL1-receptor accessory protein-like 1 (IL1RAPL1), a protein involved in cognitive functions, regulates N-type Ca2+-channel and neurite elongation.** *Proc Natl Acad Sci U S A* 2007, **104:**9063-9068.
88. Leonardo ED, Hinck L, Masu M, Keino-Masu K, Ackerman SL, Tessier-Lavigne M: **Vertebrate homologues of C. elegans UNC-5 are candidate netrin receptors.** *Nature* 1997, **386:**833-838.
89. Levy-Strumpf N, Culotti JG: **Netrins and Wnts function redundantly to regulate antero-posterior and dorso-ventral guidance in C. elegans.** *PLoS Genet* 2014, **10:**e1004381.

90. Banki K, Hutter E, Colombo E, Gonchoroff NJ, Perl A: **Glutathione levels and sensitivity to apoptosis are regulated by changes in transaldolase expression.** *J Biol Chem* 1996, **271:**32994-33001.
91. Salkoff L, Butler A, Ferreira G, Santi C, Wei A: **High-conductance potassium channels of the SLO family.** *Nat Rev Neurosci* 2006, **7:**921-931.
92. Hunt TW, Fields TA, Casey PJ, Peralta EG: **RGS10 is a selective activator of G alpha i GTPase activity.** *Nature* 1996, **383:**175-177.
93. Jeong W, Lee DY, Park S, Rhee SG: **ERp16, an endoplasmic reticulum-resident thiol-disulfide oxidoreductase: biochemical properties and role in apoptosis induced by endoplasmic reticulum stress.** *J Biol Chem* 2008, **283:**25557-25566.
94. Yorimitsu T, Nair U, Yang Z, Klionsky DJ: **Endoplasmic reticulum stress triggers autophagy.** *J Biol Chem* 2006, **281:**30299-30304.
95. Petherick KJ, Williams AC, Lane JD, Ordonez-Moran P, Huelsken J, Collard TJ, Smartt HJ, Batson J, Malik K, Paraskeva C, Greenhough A: **Autolysosomal beta-catenin degradation regulates Wnt-autophagy-p62 crosstalk.** *EMBO J* 2013, **32:**1903-1916.
96. Beard RS, Jr., Haines RJ, Wu KY, Reynolds JJ, Davis SM, Elliott JE, Malinin NL, Chatterjee V, Cha BJ, Wu MH, Yuan SY: **Non-muscle Mlck is required for beta-catenin- and FoxO1-dependent downregulation of Cldn5 in IL-1beta-mediated barrier dysfunction in brain endothelial cells.** *J Cell Sci* 2014, **127:**1840-1853.
97. Zhao X, Duester G: **Effect of retinoic acid signaling on Wnt/beta-catenin and FGF signaling during body axis extension.** *Gene Expr Patterns* 2009, **9:**430-435.
98. Piao H, Kim J, Noh SH, Kweon HS, Kim JY, Lee MG: **Sec16A is critical for both conventional and unconventional secretion of CFTR.** *Sci Rep* 2017, **7:**39887.
99. Rahman N, Buck J, Levin LR: **pH sensing via bicarbonate-regulated "soluble" adenylyl cyclase (sAC).** *Front Physiol* 2013, **4:**343.
100. Fang Y, Vilella-Bach M, Bachmann R, Flanigan A, Chen J: **Phosphatidic acid-mediated mitogenic activation of mTOR signaling.** *Science* 2001, **294:**1942-1945.
101. Murata T, Ohnishi H, Okazawa H, Murata Y, Kusakari S, Hayashi Y, Miyashita M, Itoh H, Oldenborg PA, Furuya N, Matozaki T: **CD47 promotes neuronal development through Src- and FRG/Vav2-mediated activation of Rac and Cdc42.** *J Neurosci* 2006, **26:**12397-12407.
102. Suire S, Coadwell J, Ferguson GJ, Davidson K, Hawkins P, Stephens L: **p84, a new Gbetagamma-activated regulatory subunit of the type IB phosphoinositide 3-kinase p110gamma.** *Curr Biol* 2005, **15:**566-570.
103. Lee MH, Koria P, Qu J, Andreadis ST: **JNK phosphorylates beta-catenin and regulates adherens junctions.** *FASEB J* 2009, **23:**3874-3883.
104. Pang T, Hisamitsu T, Mori H, Shigekawa M, Wakabayashi S: **Role of calcineurin B homologous protein in pH regulation by the Na+/H+ exchanger 1: tightly bound Ca2+ ions as important structural elements.** *Biochemistry* 2004, **43:**3628-3636.
105. Siyanov V, Baltz JM: **NHE1 is the sodium-hydrogen exchanger active in acute intracellular pH regulation in preimplantation mouse embryos.** *Biol Reprod* 2013, **88:**157.
106. Martin R, Torres M, Sanchez-Prieto J: **mGluR7 inhibits glutamate release through a PKC-independent decrease in the activity of P/Q-type Ca2+ channels and by diminishing cAMP in hippocampal nerve terminals.** *Eur J Neurosci* 2007, **26:**312-322.
107. Ratzinger S, Eble JA, Pasoldt A, Opolka A, Rogler G, Grifka J, Grassel S: **Collagen XVI induces formation of focal contacts on intestinal myofibroblasts isolated from the normal and inflamed intestinal tract.** *Matrix Biol* 2010, **29:**177-193.
108. Wu C, Dedhar S: **Integrin-linked kinase (ILK) and its interactors: a new paradigm for the coupling of extracellular matrix to actin cytoskeleton and signaling complexes.** *J Cell Biol* 2001, **155:**505-510.
109. Troussard AA, Mawji NM, Ong C, Mui A, St -Arnaud R, Dedhar S: **Conditional knock-out of integrin-linked kinase demonstrates an essential role in protein kinase B/Akt activation.** *J Biol Chem* 2003, **278:**22374-22378.
110. Ebert MP, Tanzer M, Balluff B, Burgermeister E, Kretzschmar AK, Hughes DJ, Tetzner R, Lofton-Day C, Rosenberg R, Reinacher-Schick AC, et al: **TFAP2E-DKK4 and chemoresistance in colorectal cancer.** *N Engl J Med* 2012, **366:**44-53.

111. Cribbs LL, Gomora JC, Daud AN, Lee JH, Perez-Reyes E: **Molecular cloning and functional expression of Ca(v)3.1c, a T-type calcium channel from human brain.** *FEBS Lett* 2000, **466:**54-58.

112. Gwak J, Cho M, Gong SJ, Won J, Kim DE, Kim EY, Lee SS, Kim M, Kim TK, Shin JG, Oh S: **Protein-kinase-C-mediated beta-catenin phosphorylation negatively regulates the Wnt/beta-catenin pathway.** *J Cell Sci* 2006, **119:**4702-4709.

113. Wang Z, Luo Z, Zhou L, Li X, Jiang T, Fu E: **DDX5 promotes proliferation and tumorigenesis of non-small-cell lung cancer cells by activating beta-catenin signaling pathway.** *Cancer Sci* 2015, **106:**1303-1312.

Supplementary Figure 3 | Thermodynamic profiles of hairpins formed by the miRNA interactome targets in human. Note that many of the miRNA targets are embedded in imperfect hairpins analogous to the secondary structure of miR-4673.

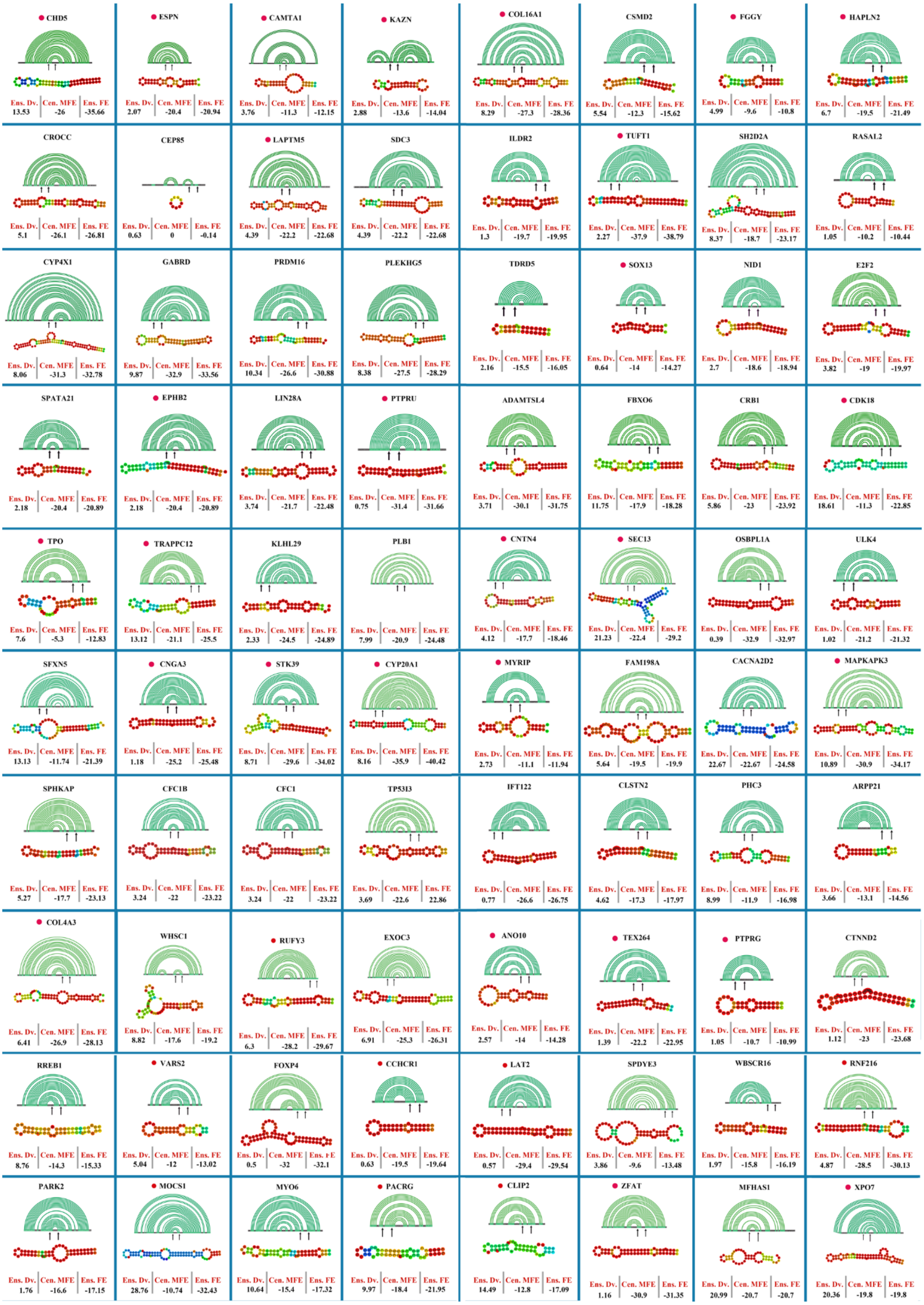

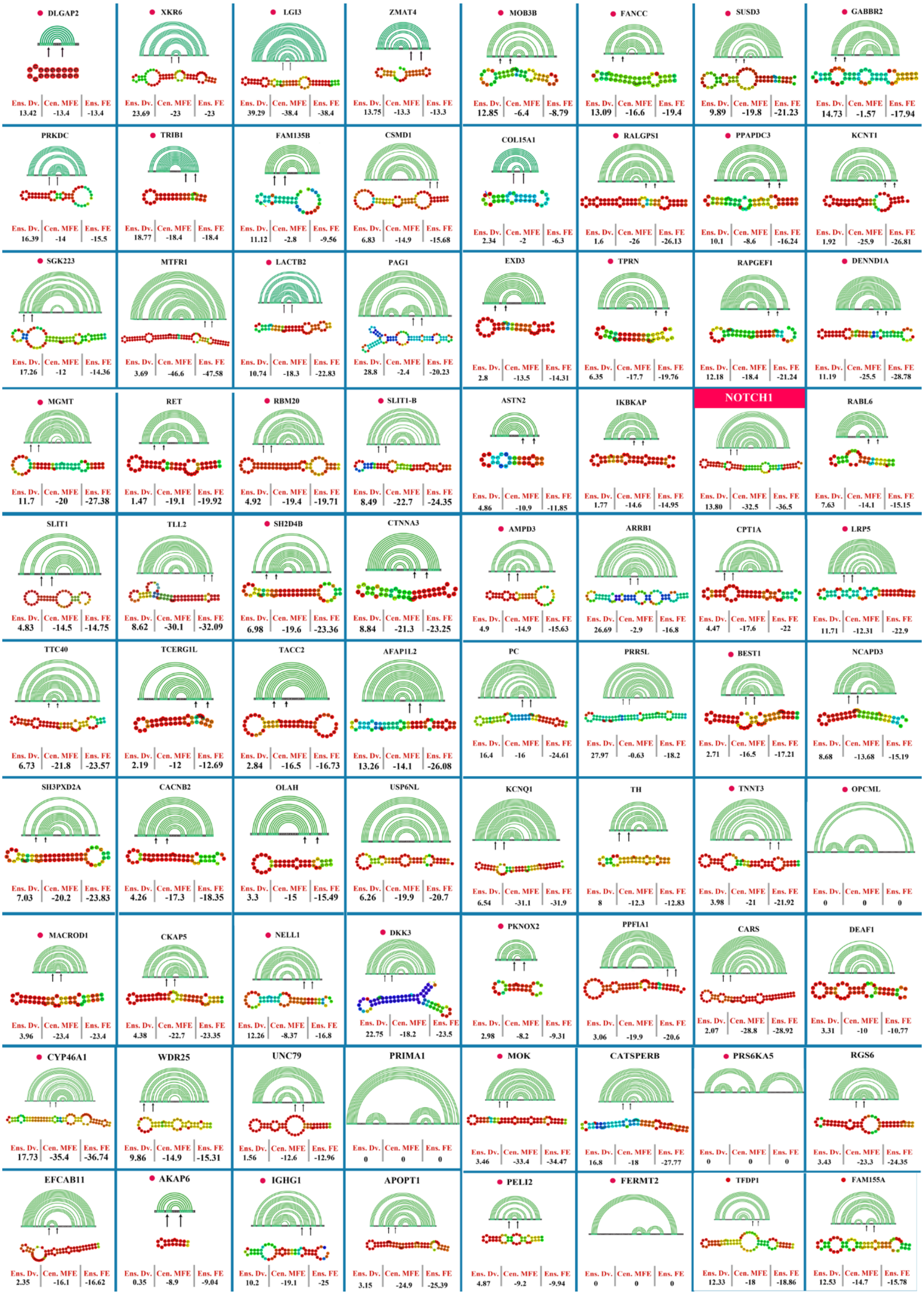
DLGAP2
Ens. Dv. 13.42 Cen. MFE -13.4 Ens. FE -13.4
XKR6
Ens. Dv. 23.69 Cen. MFE -23 Ens. FE -23
LGI3
Ens. Dv. 39.29 Cen. MFE -38.4 Ens. FE -38.4
ZMAT4
Ens. Dv. 13.75 Cen. MFE -13.3 Ens. FE -13.3
MOB3B
Ens. Dv. 12.85 Cen. MFE -6.4 Ens. FE -8.79
FANCC
Ens. Dv. 13.09 Cen. MFE -16.6 Ens. FE -19.4
SUSD3
Ens. Dv. 9.89 Cen. MFE -19.8 Ens. FE -21.23
GABBR2
Ens. Dv. 14.73 Cen. MFE -1.57 Ens. FE -17.94
PRKDC
Ens. Dv. 16.39 Cen. MFE -14 Ens. FE -15.5
TRIB1
Ens. Dv. 18.77 Cen. MFE -18.4 Ens. FE -18.4
FAM135B
Ens. Dv. 11.12 Cen. MFE -2.8 Ens. FE -9.56
CSMD1
Ens. Dv. 6.83 Cen. MFE -14.9 Ens. FE -15.68
COL15A1
Ens. Dv. 2.34 Cen. MFE -2 Ens. FE -6.3
RALGPS1
Ens. Dv. 1.6 Cen. MFE -26 Ens. FE -26.13
PPAPDC3
Ens. Dv. 10.1 Cen. MFE -8.6 Ens. FE -16.24
KCNT1
Ens. Dv. 1.92 Cen. MFE -25.9 Ens. FE -26.81
SGK223
Ens. Dv. 17.26 Cen. MFE -12 Ens. FE -14.36
MTFR1
Ens. Dv. 3.69 Cen. MFE -46.6 Ens. FE -47.58
LACTB2
Ens. Dv. 10.74 Cen. MFE -18.3 Ens. FE -22.83
PAG1
Ens. Dv. 28.8 Cen. MFE -2.4 Ens. FE -20.23
EXD3
Ens. Dv. 2.8 Cen. MFE -13.5 Ens. FE -14.31
TPRN
Ens. Dv. 6.35 Cen. MFE -17.7 Ens. FE -19.76
RAPGEF1
Ens. Dv. 12.18 Cen. MFE -18.4 Ens. FE -21.24
DENND1A
Ens. Dv. 11.19 Cen. MFE -25.5 Ens. FE -28.78
MGMT
Ens. Dv. 11.7 Cen. MFE -20 Ens. FE -27.38
RET
Ens. Dv. 1.47 Cen. MFE -19.1 Ens. FE -19.92
RBM20
Ens. Dv. 4.92 Cen. MFE -19.4 Ens. FE -19.71
SLIT1-B
Ens. Dv. 8.49 Cen. MFE -22.7 Ens. FE -24.35
ASTN2
Ens. Dv. 4.86 Cen. MFE -10.9 Ens. FE -11.85
IKBKAP
Ens. Dv. 1.77 Cen. MFE -14.6 Ens. FE -14.95
NOTCH1
Ens. Dv. 13.80 Cen. MFE -32.5 Ens. FE -36.5
RABL6
Ens. Dv. 7.63 Cen. MFE -14.1 Ens. FE -15.15
SLIT1
Ens. Dv. 4.83 Cen. MFE -14.5 Ens. FE -14.75
TLL2
Ens. Dv. 8.62 Cen. MFE -30.1 Ens. FE -32.09
SH2D4B
Ens. Dv. 6.98 Cen. MFE -19.6 Ens. FE -23.36
CTNNA3
Ens. Dv. 8.84 Cen. MFE -21.3 Ens. FE -23.25
AMPD3
Ens. Dv. 4.9 Cen. MFE -14.9 Ens. FE -15.63
ARRB1
Ens. Dv. 26.69 Cen. MFE -2.9 Ens. FE -16.8
CPT1A
Ens. Dv. 4.47 Cen. MFE -17.6 Ens. FE -22
LRP5
Ens. Dv. 11.71 Cen. MFE -12.31 Ens. FE -22.9
TTC40
Ens. Dv. 6.73 Cen. MFE -21.8 Ens. FE -23.57
TCERG1L
Ens. Dv. 2.19 Cen. MFE -12 Ens. FE -12.69
TACC2
Ens. Dv. 2.84 Cen. MFE -16.5 Ens. FE -16.73
AFAP1L2
Ens. Dv. 13.26 Cen. MFE -14.1 Ens. FE -26.08
PC
Ens. Dv. 16.4 Cen. MFE -16 Ens. FE -24.61
PRR5L
Ens. Dv. 27.97 Cen. MFE -0.63 Ens. FE -18.2
BEST1
Ens. Dv. 2.71 Cen. MFE -16.5 Ens. FE -17.21
NCAPD3
Ens. Dv. 8.68 Cen. MFE -13.68 Ens. FE -15.19
SH3PXD2A
Ens. Dv. 7.03 Cen. MFE -20.2 Ens. FE -23.83
CACNB2
Ens. Dv. 4.26 Cen. MFE -17.3 Ens. FE -18.35
OLAH
Ens. Dv. 3.3 Cen. MFE -15 Ens. FE -15.49
USP6NL
Ens. Dv. 6.26 Cen. MFE -19.9 Ens. FE -20.7
KCNQ1
Ens. Dv. 6.54 Cen. MFE -31.1 Ens. FE -31.9
TH
Ens. Dv. 8 Cen. MFE -12.3 Ens. FE -12.83
TNNT3
Ens. Dv. 3.98 Cen. MFE -21 Ens. FE -21.92
OPCML
Ens. Dv. 0 Cen. MFE 0 Ens. FE 0
MACROD1
Ens. Dv. 3.96 Cen. MFE -23.4 Ens. FE -23.4
CKAP5
Ens. Dv. 4.38 Cen. MFE -22.7 Ens. FE -23.35
NELL1
Ens. Dv. 12.26 Cen. MFE -8.37 Ens. FE -16.8
DKK3
Ens. Dv. 22.75 Cen. MFE -18.2 Ens. FE -23.5
PKNOX2
Ens. Dv. 2.98 Cen. MFE -8.2 Ens. FE -9.31
PPFIA1
Ens. Dv. 3.06 Cen. MFE -19.9 Ens. FE -20.6
CARS
Ens. Dv. 2.07 Cen. MFE -28.8 Ens. FE -28.92
DEAF1
Ens. Dv. 3.31 Cen. MFE -10 Ens. FE -10.77
CYP46A1
Ens. Dv. 17.73 Cen. MFE -35.4 Ens. FE -36.74
WDR25
Ens. Dv. 9.86 Cen. MFE -14.9 Ens. FE -15.31
UNC79
Ens. Dv. 1.56 Cen. MFE -12.6 Ens. FE -12.96
PRIMA1
Ens. Dv. 0 Cen. MFE 0 Ens. FE 0
MOK
Ens. Dv. 3.46 Cen. MFE -33.4 Ens. FE -34.47
CATSPERB
Ens. Dv. 16.8 Cen. MFE -18 Ens. FE -27.77
PRS6KA5
Ens. Dv. 0 Cen. MFE 0 Ens. FE 0
RGS6
Ens. Dv. 3.43 Cen. MFE -23.3 Ens. FE -24.35
EFCAB11
Ens. Dv. 2.35 Cen. MFE -16.1 Ens. FE -16.62
AKAP6
Ens. Dv. 0.35 Cen. MFE -8.9 Ens. FE -9.04
IGHG1
Ens. Dv. 10.2 Cen. MFE -19.1 Ens. FE -25
APOPT1
Ens. Dv. 3.15 Cen. MFE -24.9 Ens. FE -25.39
PELI2
Ens. Dv. 4.87 Cen. MFE -9.2 Ens. FE -9.94
FERMT2
Ens. Dv. 0 Cen. MFE 0 Ens. FE 0
TFDP1
Ens. Dv. 12.33 Cen. MFE -18 Ens. FE -18.86
FAM155A
Ens. Dv. 12.53 Cen. MFE -14.7 Ens. FE -15.78

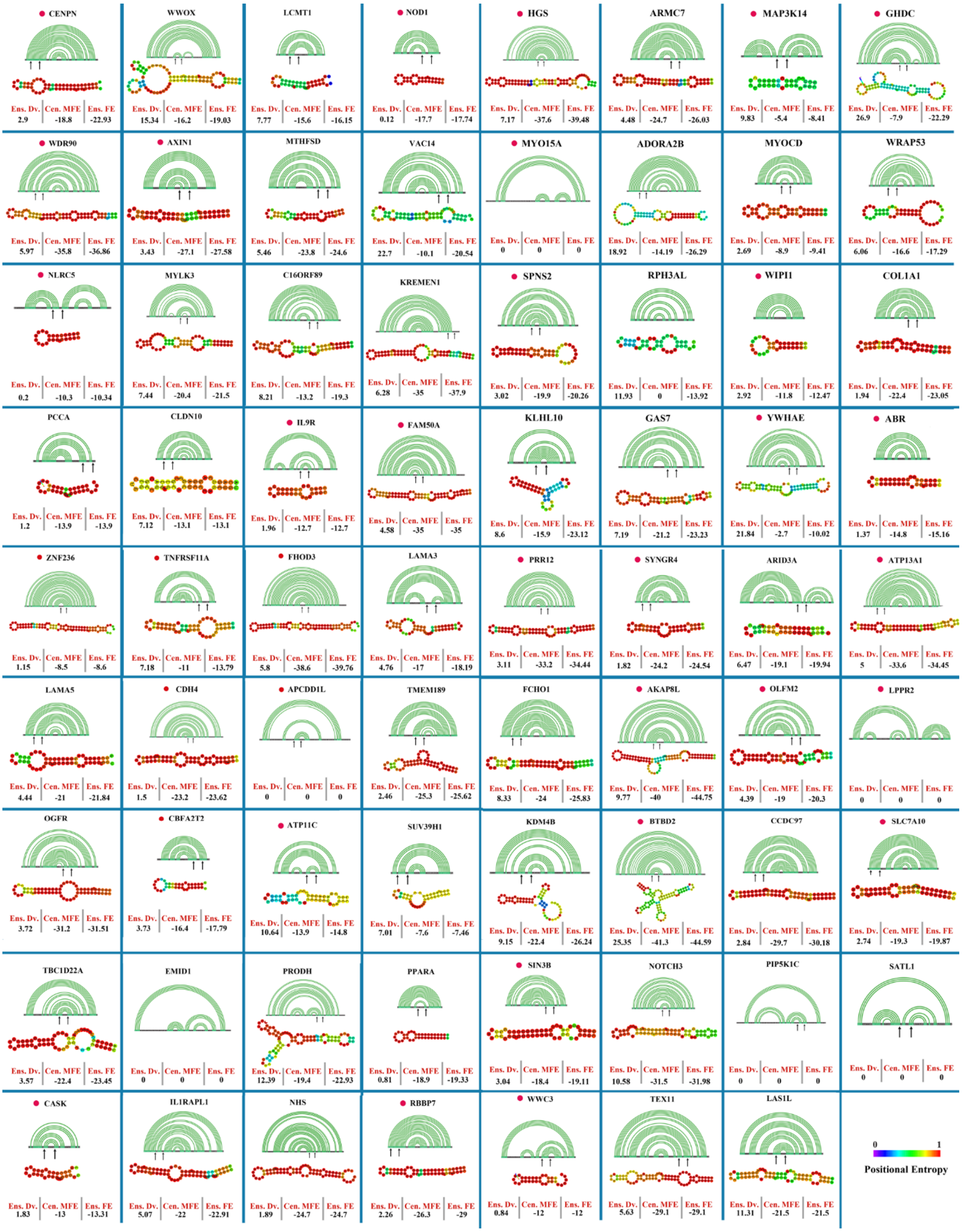

CENPN
Ens. Dv. 2.9 | Cen. MFE -18.8 | Ens. FE -22.93
WWOX
Ens. Dv. 15.34 | Cen. MFE -16.2 | Ens. FE -19.03
LCMT1
Ens. Dv. 7.77 | Cen. MFE -15.6 | Ens. FE -16.15
NOD1
Ens. Dv. 0.12 | Cen. MFE -17.7 | Ens. FE -17.74
HGS
Ens. Dv. 7.17 | Cen. MFE -37.6 | Ens. FE -39.48
ARMC7
Ens. Dv. 4.48 | Cen. MFE -24.7 | Ens. FE -26.03
MAP3K14
Ens. Dv. 9.83 | Cen. MFE -5.4 | Ens. FE -8.41
GHDC
Ens. Dv. 26.9 | Cen. MFE -7.9 | Ens. FE -22.29
WDR90
Ens. Dv. 5.97 | Cen. MFE -35.8 | Ens. FE -36.86
AXIN1
Ens. Dv. 3.43 | Cen. MFE -27.1 | Ens. FE -27.58
MTHFSD
Ens. Dv. 5.46 | Cen. MFE -23.8 | Ens. FE -24.6
VAC14
Ens. Dv. 22.7 | Cen. MFE -10.1 | Ens. FE -20.54
MYO15A
Ens. Dv. 0 | Cen. MFE 0 | Ens. FE 0
ADORA2B
Ens. Dv. 18.92 | Cen. MFE -14.19 | Ens. FE -26.29
MYOCD
Ens. Dv. 2.69 | Cen. MFE -8.9 | Ens. FE -9.41
WRAP53
Ens. Dv. 6.06 | Cen. MFE -16.6 | Ens. FE -17.29
NLRC5
Ens. Dv. 0.2 | Cen. MFE -10.3 | Ens. FE -10.34
MYLK3
Ens. Dv. 7.44 | Cen. MFE -20.4 | Ens. FE -21.5
C16ORF89
Ens. Dv. 8.21 | Cen. MFE -13.2 | Ens. FE -19.3
KREMEN1
Ens. Dv. 6.28 | Cen. MFE -35 | Ens. FE -37.9
SPNS2
Ens. Dv. 3.02 | Cen. MFE -19.9 | Ens. FE -20.26
RPH3AL
Ens. Dv. 11.93 | Cen. MFE 0 | Ens. FE -13.92
WIPI1
Ens. Dv. 2.92 | Cen. MFE -11.8 | Ens. FE -12.47
COL1A1
Ens. Dv. 1.94 | Cen. MFE -22.4 | Ens. FE -23.05
PCCA
Ens. Dv. 1.2 | Cen. MFE -13.9 | Ens. FE -13.9
CLDN10
Ens. Dv. 7.12 | Cen. MFE -13.1 | Ens. FE -13.1
IL9R
Ens. Dv. 1.96 | Cen. MFE -12.7 | Ens. FE -12.7
FAM50A
Ens. Dv. 4.58 | Cen. MFE -35 | Ens. FE -35
KLHL10
Ens. Dv. 8.6 | Cen. MFE -15.9 | Ens. FE -23.12
GAS7
Ens. Dv. 7.19 | Cen. MFE -21.2 | Ens. FE -23.23
YWHAE
Ens. Dv. 21.84 | Cen. MFE -2.7 | Ens. FE -10.02
ABR
Ens. Dv. 1.37 | Cen. MFE -14.8 | Ens. FE -15.16
ZNF236
Ens. Dv. 1.15 | Cen. MFE -8.5 | Ens. FE -8.6
TNFRSF11A
Ens. Dv. 7.18 | Cen. MFE -11 | Ens. FE -13.79
FHOD3
Ens. Dv. 5.8 | Cen. MFE -38.6 | Ens. FE -39.76
LAMA3
Ens. Dv. 4.76 | Cen. MFE -17 | Ens. FE -18.19
PRR12
Ens. Dv. 3.11 | Cen. MFE -33.2 | Ens. FE -34.44
SYNGR4
Ens. Dv. 1.82 | Cen. MFE -24.2 | Ens. FE -24.54
ARID3A
Ens. Dv. 6.47 | Cen. MFE -19.1 | Ens. FE -19.94
ATP13A1
Ens. Dv. 5 | Cen. MFE -33.6 | Ens. FE -34.45
LAMA5
Ens. Dv. 4.44 | Cen. MFE -21 | Ens. FE -21.84
CDH4
Ens. Dv. 1.5 | Cen. MFE -23.2 | Ens. FE -23.62
APCDD1L
Ens. Dv. 0 | Cen. MFE 0 | Ens. FE 0
TMEM189
Ens. Dv. 2.46 | Cen. MFE -25.3 | Ens. FE -25.62
FCHO1
Ens. Dv. 8.33 | Cen. MFE -24 | Ens. FE -25.83
AKAP8L
Ens. Dv. 9.77 | Cen. MFE -40 | Ens. FE -44.75
OLFM2
Ens. Dv. 4.39 | Cen. MFE -19 | Ens. FE -20.3
LPPR2
Ens. Dv. 0 | Cen. MFE 0 | Ens. FE 0
OGFR
Ens. Dv. 3.72 | Cen. MFE -31.2 | Ens. FE -31.51
CBFA2T2
Ens. Dv. 3.73 | Cen. MFE -16.4 | Ens. FE -17.79
ATP11C
Ens. Dv. 10.64 | Cen. MFE -13.9 | Ens. FE -14.8
SUV39H1
Ens. Dv. 7.01 | Cen. MFE -7.6 | Ens. FE -7.46
KDM4B
Ens. Dv. 9.15 | Cen. MFE -22.4 | Ens. FE -26.24
BTBD2
Ens. Dv. 25.35 | Cen. MFE -41.3 | Ens. FE -44.59
CCDC97
Ens. Dv. 2.84 | Cen. MFE -29.7 | Ens. FE -30.18
SLC7A10
Ens. Dv. 2.74 | Cen. MFE -19.3 | Ens. FE -19.87
TBC1D22A
Ens. Dv. 3.57 | Cen. MFE -22.4 | Ens. FE -23.45
EMID1
Ens. Dv. 0 | Cen. MFE 0 | Ens. FE 0
PRODH
Ens. Dv. 12.39 | Cen. MFE -19.4 | Ens. FE -22.93
PPARA
Ens. Dv. 0.81 | Cen. MFE -18.9 | Ens. FE -19.33
SIN3B
Ens. Dv. 3.04 | Cen. MFE -18.4 | Ens. FE -19.11
NOTCH3
Ens. Dv. 10.58 | Cen. MFE -31.5 | Ens. FE -31.98
PIP5K1C
Ens. Dv. 0 | Cen. MFE 0 | Ens. FE 0
SATL1
Ens. Dv. 0 | Cen. MFE 0 | Ens. FE 0
CASK
Ens. Dv. 1.83 | Cen. MFE -13 | Ens. FE -13.31
IL1RAPL1
Ens. Dv. 5.07 | Cen. MFE -22 | Ens. FE -22.91
NHS
Ens. Dv. 1.89 | Cen. MFE -24.7 | Ens. FE -24.7
RBBP7
Ens. Dv. 2.26 | Cen. MFE -26.3 | Ens. FE -29
WWC3
Ens. Dv. 0.84 | Cen. MFE -12 | Ens. FE -12
TEX11
Ens. Dv. 5.63 | Cen. MFE -29.1 | Ens. FE -29.1
LAS1L
Ens. Dv. 11.31 | Cen. MFE -21.5 | Ens. FE -21.5
0
1
Positional Entropy

Supplementary Figure 4. Full blots of eRNA fingerprint fron Figure 1d.

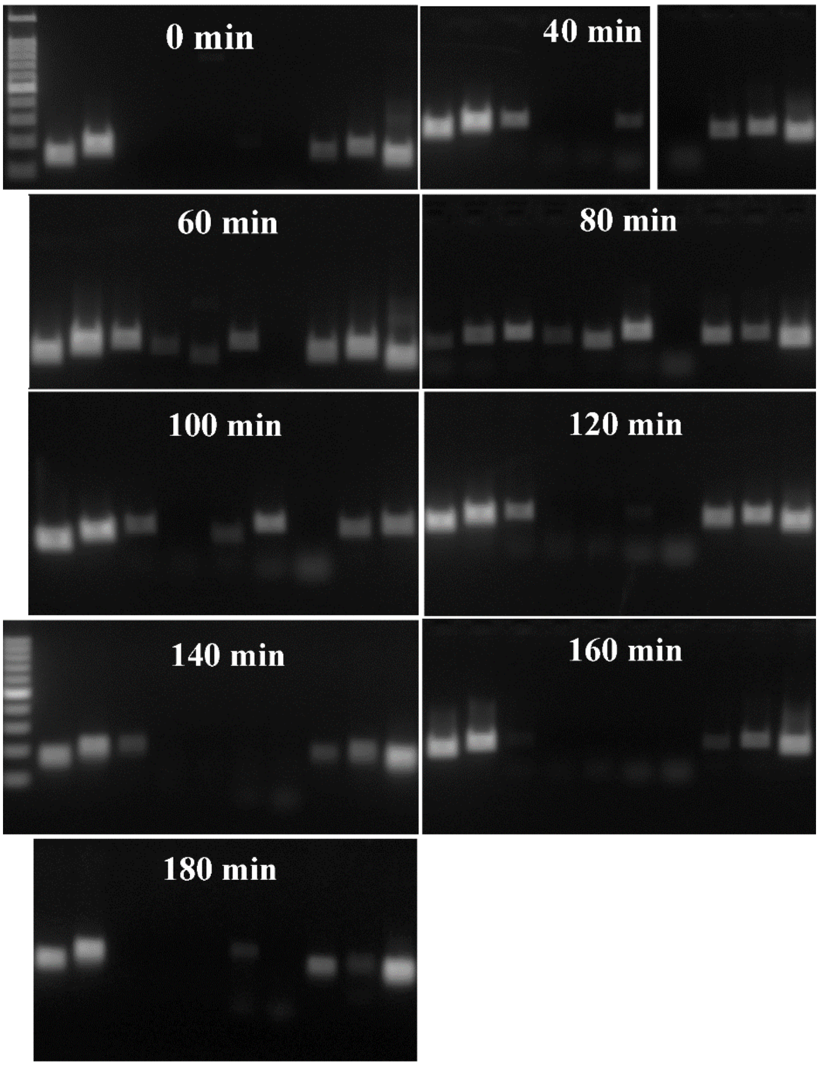

Supplementary Figure 5

Temporal variation of eRNA profile generated from intron 4 enhancer.

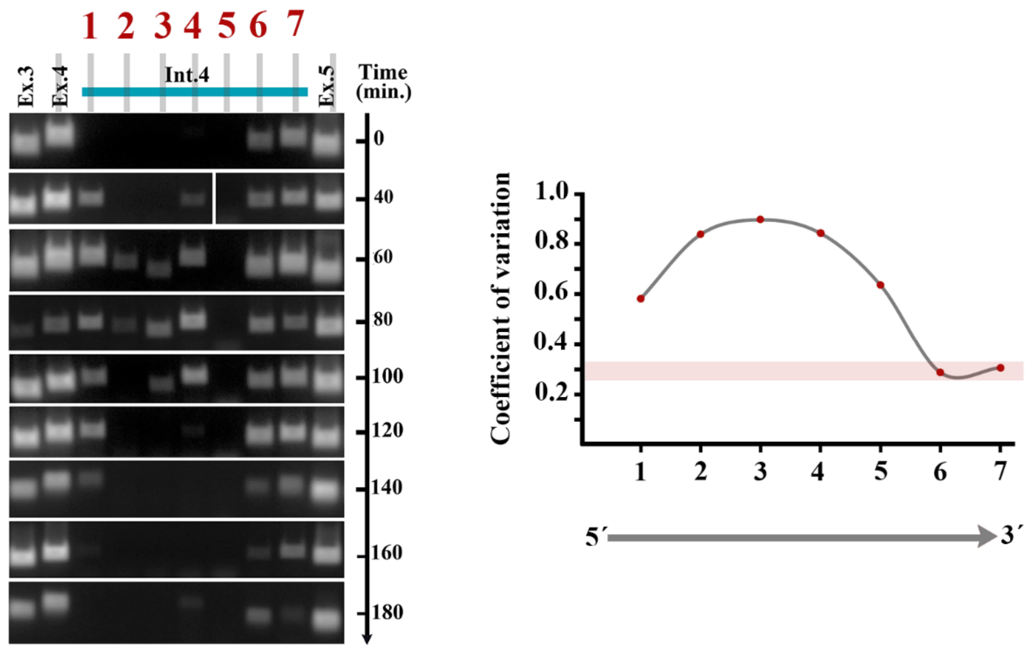

In order to generate the temporal variation of eRNA, pixel intensities of bands were measured and the coefficient of variation (standard deviation/mean intensity) in each region (bands 1-7, t=0-180 min) was generated based on the pixel intensities. Note that eRNA-7 and eRNA-6 (corresponding to 3´-enhancer) show the least temporal variability amongst the eRNAs.
